# The wooden artifacts from Schöningen’s Spear Horizon and their place in human evolution

**DOI:** 10.1073/pnas.2320484121

**Published:** 2024-04-01

**Authors:** Dirk Leder, Jens Lehmann, Annemieke Milks, Tim Koddenberg, Michael Sietz, Matthias Vogel, Utz Böhner, Thomas Terberger

**Affiliations:** ^a^Department of Archaeology, Lower Saxony State Office for Cultural Heritage, Hannover 30175, Germany; ^b^Department of Archaeology, University of Reading, Earley, Reading RG6 6AX, United Kingdom; ^c^Department of Wood Biology and Wood Products, Georg-August University Göttingen, Gottingen 37077, Germany; ^d^Archaeological Conservation Unit, Lower Saxony State Office for Cultural Heritage, Hannover 30175, Germany; ^e^Department of Prehistoric Archaeology, Georg-August University Göttingen, Gottingen 37073, Germany

**Keywords:** Schöningen, wooden artifacts, wood technology, group hunting, human evolution

## Abstract

Ethnographic records show that wooden tools played a pivotal role in the daily lives of hunter-gatherers including food procurement tools used in hunting (e.g., spears, throwing sticks) and gathering (e.g. digging sticks, bark peelers), as well as, domestic tools (e.g., handles, vessels). However, wood rarely survives in the archeological record, especially in Pleistocene contexts and knowledge of prehistoric hunter-gatherer lifeways is strongly biased by the survivorship of more resilient materials such as lithics and bones. Consequently, very few Paleolithic sites have produced wooden artifacts and among them, the site of Schöningen stands out due to its number and variety of wooden tools. The recovery of complete wooden spears and throwing sticks at this 300,000-y-old site (MIS 9) led to a paradigm shift in the hunter vs. scavenger debate. For the first time and almost 30 y after their discovery, this study introduces the complete wooden assemblage from Schöningen 13 II-4 known as the Spear Horizon. In total, 187 wooden artifacts could be identified from the Spear Horizon demonstrating a broad spectrum of wood-working techniques, including the splitting technique. A minimum of 20 hunting weapons is now recognized and two newly identified artifact types comprise 35 tools made on split woods, which were likely used in domestic activities. Schöningen 13 II-4 represents the largest Pleistocene wooden artifact assemblage worldwide and demonstrates the key role woodworking had in human evolution. Finally, our results considerably change the interpretation of the Pleistocene lakeshore site of Schöningen.

## Significance

Wooden tools rarely survive in the Paleolithic record limiting our understanding of Pleistocene hunter-gather lifeways. With 187 wooden artifacts, Schöningen 13 II-4 provides the largest assemblage worldwide introduced here for the first time in full. Wooden tools include at least 10 spears and seven throwing sticks used in hunting next to 35 newly recognized pointed and rounded split woods likely used in domestic activities. The study provides unique insights into Pleistocene woodworking techniques, tool design, use, re-working, and human behavior connected to wooden artifacts. Human evolution studies show increasing brain size and technological complexity that coincide with human group hunting over the last 2 Ma. Schöningen’s wooden hunting weapons exemplify the interplay of technological complexity, human behavior, and human evolution.

## Pleistocene Wooden Tools.

The earliest indirect evidence for human woodworking dates back 2 to 1.5 Ma ago based on use-wear on lithics (1, 2). Direct evidence of wood artifacts coming from Africa and the Middle East date back to 780 ka BP (3, 4). The discovery of early wooden hunting weapons, such as spears and throwing sticks, has revolutionized our understanding of early human hunting abilities, social interaction, and hominin cognition. The earliest wooden spears in Europe are 400 to 120 ka old, with an outstanding assemblage from Schöningen (5–9). The earliest throwing sticks are known from Schöningen (5, 10, 11), with later possible examples from Africa (12, 13). The oldest arrows from the German site Stellmoor are of Late Glacial origin dating c. 11.6 ka BP (14, 15). Digging sticks used in procuring underground storage orangs are preserved at few sites in Africa, Eurasia, and South America being 400 to 14.5 ka old (13, 16–20). Early domestic wooden tools have been reported from a few sites in Eurasia and South America (4, 18, 21, 22). Finally, the Late Glacial Shigir idol from Russia represents the earliest known monumental sculpture (23).

## Importance of Schöningen.

Schöningen is located in hilly terrain in the northern European Plains (*SI Appendix*, Fig. S1). Archeological excavations at this former opencast mine commenced in 1981 delivering multiple Middle Pleistocene sites. The oldest wooden tools come from sites Schöningen 12 II, 12 B, and 13 DB, and contain about 30 slotted handles (24). Most important are the ten spears and two double-pointed sticks (DPS) or throwing sticks from Schöningen 13 II-4 (7, 10, 11, 24, 25), which led to a paradigm shift in the hunter vs. scavenger debate (5, 9). Schöningen 13 II-4 is located at a former interglacial lakeshore, which formed atop an Elsterian glacial till during MIS 9 (337 to 300 ka ago) (26). Due to lake level fluctuations, five siltation cycles are distinguished, whereby most wooden artifacts were deposited at the lakeshore during cycle 4 under the influence of low-energy fluviatile sediments in a shallow-water delta plain (27). The major occupation occurred in an open woodland landscape with alder, birch, and willow near riverine and lacustrine locations, pine trees in lowland and hilly areas, and stands of pine, spruce, and larch at higher altitudes (26). Exceptional preservation led to the survival of hundreds of natural and worked wood remains making Schöningen 13 II-4 a prime location for the study of early wooden artifacts and human behavior connected to woodworking. We present results of a systematic study on all the worked wooden artifacts from Schöningen 13 II-4 excavated up until 2008 introducing formerly unrecognized tools, two tool types, and a woodworking technique that have not been reported from Paleolithic contexts thus far.

## Results

### Overview Wooden Assemblage.

Spruce (*Picea* sp.), willow (*Salix* sp.), and pine (*Pinus sylvestris*) dominate the wooden assemblage followed by smaller numbers of birch (*Betula* sp.), poplar (*Populus* sp.), and larch (*Larix* sp.), with few specimens of fir (*Abies alba*), alder (*Alnus* sp.), juniper (*Juniperus* sp.), and oak (*Quercus* sp.) (*SI Appendix*, Table S1). The assemblage contains 187 wood artifacts (category 1: 62; category 2: 19, category 3: 106) while 527 IDs of category 4 bear no traces of human manipulation. (*SI Appendix*, Table S2). Wooden artifacts (categories 1 to 3) are exclusively made from spruce (n = 124), spruce/larch (n = 18), and pine (n = 45) (*SI Appendix*, Table S3) while only 4.1% (n = 29) of the category 4 items without diagnostic tool marks are from those wood species. Given this exclusivity, it is very likely that all spruce, spruce/larch, and pine items in category 4 are indeed artifacts even when diagnostic traces are lacking. Contrastingly, specimens of other wood species show no signs of human manipulation or use and thus represent part of the natural background vegetation.

The ratio of spruce plus spruce/larch to pine items decreases from 3.8 in category 1 to 1.1 in category 4 meaning that the former more often provides clear artifacts. Paleoenvironmental studies show the raw material was not available at the lakeshore but must have been transported from the nearby Elm Mountain some 3 to 5 km away or locations farther afield like the Harz Mountains (26).

The majority of the spruce, spruce/larch, and pine woods (91.9%) comes from the organic mud sub-level 4b (n = 114), the calcareous mud 4c (n = 16), and the contact zone between them 4 b/c (n = 40) (*SI Appendix*, Table S4). Wood remains thus preserved under waterlogged conditions, mostly along the former lakeshore (sub-level 4b) while few items were lost/tossed into the lake (sub-unit 4c). The spears were deposited in the center of the excavated lakeshore in an area extending 25 m across (*SI Appendix*, Fig. S23 and *SI-Text*). Further tools and working debris occur here too, but also north of it. Few tools were found south of the spear area as well as in the former lake together with some working debris.

**Fig. 1.**
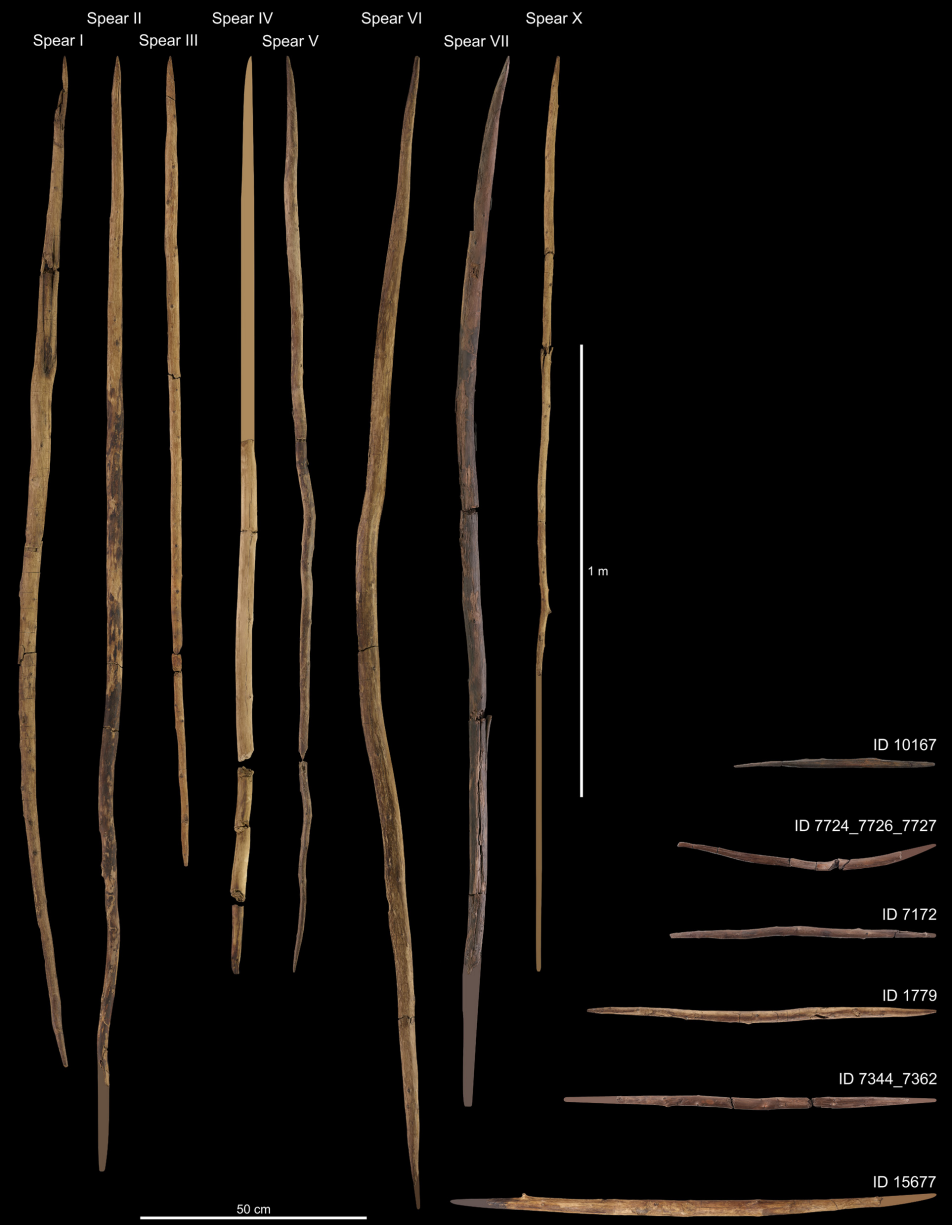
The securely identified eight spears and six DPSs from Schöningen 13 II-4 excavated until 2008. Note: Former spears VIII and IX (7) are now classified as point fragments. Spear fragments are supplemented by adding drawn elements to the figure following three steps: 1) by extending the outline of tapering ends into complete points, 2) by adding 40 cm for missing front points and 30 cm for back points in accordance with median values of all spears whenever broken ends do not taper, and 3) by extending spear outlines up to 202.7 cm in accordance with the mean length of all complete spears. Accordingly, missing points in DPSs were completed using the outlines of the tapering ends. Photos: Minkusimages; Matthias Vogel, NLD.

Our systematic study leads to the secure identification of eight spears, six DPSs (Fig. 1), and 17 point and 13 shaft fragments respectively, resulting in between ten and 18 reconstructed spears and between six and nine reconstructed DPSs (*SI Appendix*, *SI-Text* and Tables S5–S16 and S20–S22). An isolated DPS was discovered more than 80 m southeast of the spear concentration in more recent excavations (10) resulting in a total of 20 to 25 hunting weapons present at Schöningen 13 II-4. With the exception of two DPSs made on split woods (1× spruce/larch, 1× pine) and one made from a branch (1× spruce), all other hunting weapons are manufactured most likely from tree trunks (30× spruce, 1× spruce/larch, 12× pine). The wooden artifacts are mainly shaped by carving/planing and tips by splitting away small wood chips from the point toward the shaft. Chopping was not applied in tool shaping. Thereafter, the entire artifact was smoothed via scraping and abrasion.

**Fig. 2.**
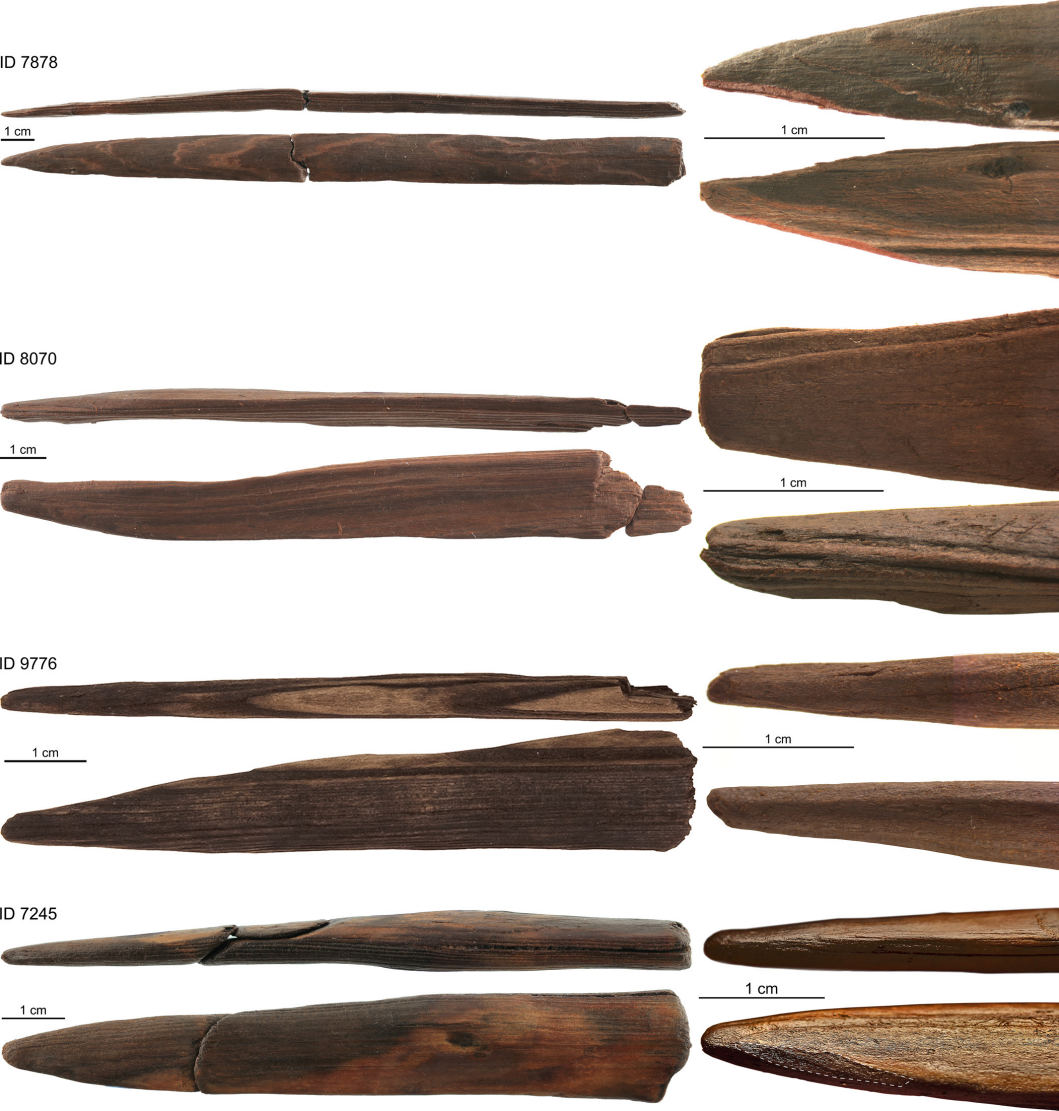
Examples of pointed split woods and close-up images of the worked point. Photos: Matthias Vogel, Jens Lehmann, Dirk Leder, NLD.

**Fig. 3.**
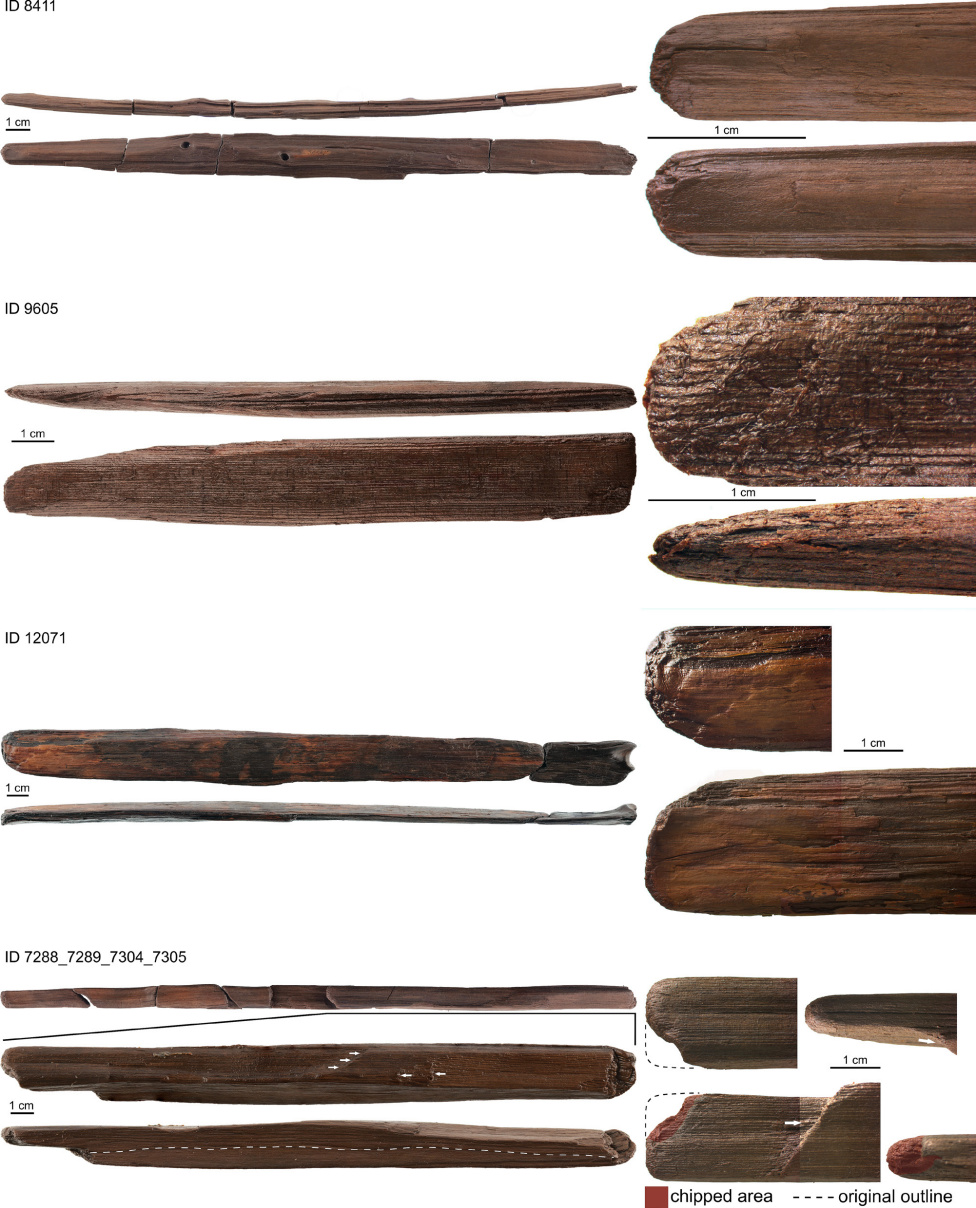
Examples of round-ended split woods and close-up images of the worked tool end. Photos: Matthias Vogel, Jens Lehmann, Dirk Leder, NLD.

Two formerly recognized tool types comprise 24 pointed split woods (Fig. 2) and 11 split woods with a rounded end (Fig. 3) that are worked mainly by splitting, scraping, and abrasion (*SI Appendix*, *SI-Text* and Tables S17 and S18). The splitting technique is recognized in a Pleistocene context, which was formerly known only from Holocene contexts (28). Besides tools and tool fragments, the wooden assemblage contains 109 split woods/splinters and fragments considered as working debris (*SI Appendix*, *SI-Text* and Table S5).

### Hunting Weapons.

Spears and DPSs comprise the spectrum of the 20 to 25 hunting weapons at Schöningen 13 II-4 (Fig. 1). Spears were both, thrusting and throwing weapons (5, 29–31) used in hunting medium-sized to large animals at Schöningen like horse, bovids, and cervids (32). DPSs are commonly interpreted as throwing sticks used in hunting small to medium-sized animals potentially including small–fast prey like birds and hare (11).

Schöningen’s complete spears (n = 3) are 1.8 to 2.5 m long (Fig. 1 and *SI Appendix*, *SI-Text* and Table S6) with a diameter of 2.3 to 4.5 cm (median = 3.5 cm). Compared to ethnographic spears, the Schöningen specimens are relatively short and thick (*SI Appendix*, *SI-Text* and Fig. S16). The location of the maximum diameter (LMD%) in Schöningen spears (median = 26.7) fits within the range of known ethnographic throwing spears (*SI Appendix*, *SI-Text* and Table S20; 33) suggesting a balance point at or in front of the midpoint. The eight spears have 18 to 60 very dense annual growth rings (median = 51) providing hardness and elasticity in these softwoods at the same time (7, 34, 35). Spears have a front point with the pith exiting along its side making them more durable (7, 36), a long straight shaft, and a back point with the pith in its center. Spears were debarked by scraping and/or cutting and tearing while branches were removed by cutting and tearing. Points were shaped by splitting the wood from the tip toward the shaft. Knots on the front point are flush with the surface while others can be flush or protruding. Surfaces are usually smoothed by scraping and/or abrading. The spears are thoroughly worked and combined with the technological design speak for a fine workmanship. Most points bear dark discolorations, which might be connected to heat treatment in an attempt to dry and harden them (37), contact with soil, blood, or grease. Longitudinal crushing on six points evidence usage as do six shaft breaks. Four broken front points are reworked by splitting and subsequent smoothing, which can be understood as a quick way to repair a broken spear point, possibly during a hunt (*SI Appendix*, Figs. S3*G*, S4, and S10).

The six DPSs were shaped essentially similar to the wooden spears with few noteworthy exceptions (Fig. 1 and *SI Appendix*, *SI-Text* and Table S7). Two DPSs were shaped on split woods rather than roundwoods, and one item was produced on a branch rather than a trunk (11). Unlike spears, both points are placed offset. Two DPSs are intact while the others have points missing. All DPSs are 45.0 to 87.7 cm long (median = 64.5 cm) with maximum diameters of 1.7 to 3.0 cm (median = 2.4 cm). The LMD of the two complete items is located at about around two-thirds of the total length (60.9% and 66.7% of length). Even when all DPSs are included, this is the most consistent variable in DPSs (coefficient of variation (CV) = 16%), suggesting that this is an important feature for flight (*SI Appendix*, *SI-Text* and Table S21). Use-wear on DPSs is present in the form of crushed points in all items and shaft breaks in three. Two points have been reworked by splitting and surface smoothing similar to the spears (*SI Appendix*, Figs. S7 and S8).

### Domestic Tools.

The two tool categories made on split woods likely represent domestic tools. These comprise 24 split woods with a single pointed tool end (Fig. 2 and *SI Appendix*, *SI-Text* and Table S17) and 11 split woods with a rounded tool end (Fig. 3 and *SI Appendix*, *SI-Text* and Table S18). The usage of split woods as blanks sets them apart from spears and point fragments while the single tapering/rounded tool end differentiates them from DPSs and shaft fragments.

The 24 pointed split woods are defined by their single tapering end (Fig. 2 and *SI Appendix*, *SI-Text* and Table S17). Seventeen are made from spruce, five from spruce/larch, and two from pine. Lengths vary between 4.6 cm and 36.0 cm (median = 14.5 cm). Maximum widths range from 0.8 to 2.7 cm (median = 1.4 cm) while maximum thickness varies between 0.4 and 1.9 cm (median = 0.7 cm) meaning particularly slender split woods were used. Knots are almost absent while 10 artifacts have debarked surfaces with tool marks that compare well to other tools speaking for the recycling of former roundwood artifacts (*SI Appendix, SI-Text*). All other items solely consist of split surfaces. Besides longitudinal splitting, the tools are further worked using localized splitting and smoothing. Smoothed areas cover complete surfaces on 16 objects while only the tip was abraded in eight. The production sequence is thus fairly standardized including splitting and smoothing to form small points on flawless split woods. Use-wear might be present in the form of use-polish on preserved tips. Crushing and micro-splintering have been observed in six tips providing clear evidence of use. Two items bear dark discoloration that might result from charring or residue.

The assemblage also contains 11 split woods with a round tool end (Fig. 3 and *SI Appendix*, *SI-Text* and Table S18). Lengths vary between 14.5 cm and 82.8 cm (median = 27.0 cm). The maximum width ranges from 1.7 cm to 3.8 cm (median = 2.6 cm) while maximum thickness ranges from 0.7 to 2.5 cm (median = 1.1 cm). In comparison to pointed split woods, sturdier blanks were selected (median lengths is 27.0: 14.5 cm, widths is 2.6: 1.4 cm, and thickness is 1.1: 0.7 cm). The *chaîne opératoire* compares well with that of the pointed split woods including similar working traces. Eight artifacts have debarked surfaces with tool marks comparable to those of artifacts made from roundwoods, which might have been recycled to shape these tools (*SI Appendix, SI-Text*). Natural flaws are absent speaking for a thorough selection of wood properties. Use-wear might be present in the form of use-polish at the rounded tool ends, micro-splintering has been detected on eight objects, in two cases combined with longitudinal crushing. Orange-brown coating and flecks on surfaces might be residue from tool-use (Fig. 3, ID 12071).

Considering comparably shaped bone and antler tools from archeological records (38), usage as awls used in working soft materials seems plausible for the pointed split woods. Other functions, for example, as pins for hair or clothes, for extracting insects from tree bark or as fishing spear points are possible too. Use-wear at the tips of pointed split woods such as polish, crushing, and micro-splintering might point to slightly abrasive tasks, whereas oblique striations typical for digging sticks are absent (16). The split woods with a round tool end morphologically compare well with hide smoothers made from bone and ivory (*SI Appendix*, Fig. S15; 39, 40). However, other functions comparable to those of bone spatulas, e.g., sewing reed mats, scaling fish, and folding bark containers (41), cannot be excluded. Use-wear in the form polish, micro-splintering, and crushing present on round-ended tools might be indicative of slightly abrasive tasks. Oblique striations typical for digging sticks are absent (16).

### Wooden Artifacts *Chaîne Opératoire*.

**Fig. 4.**
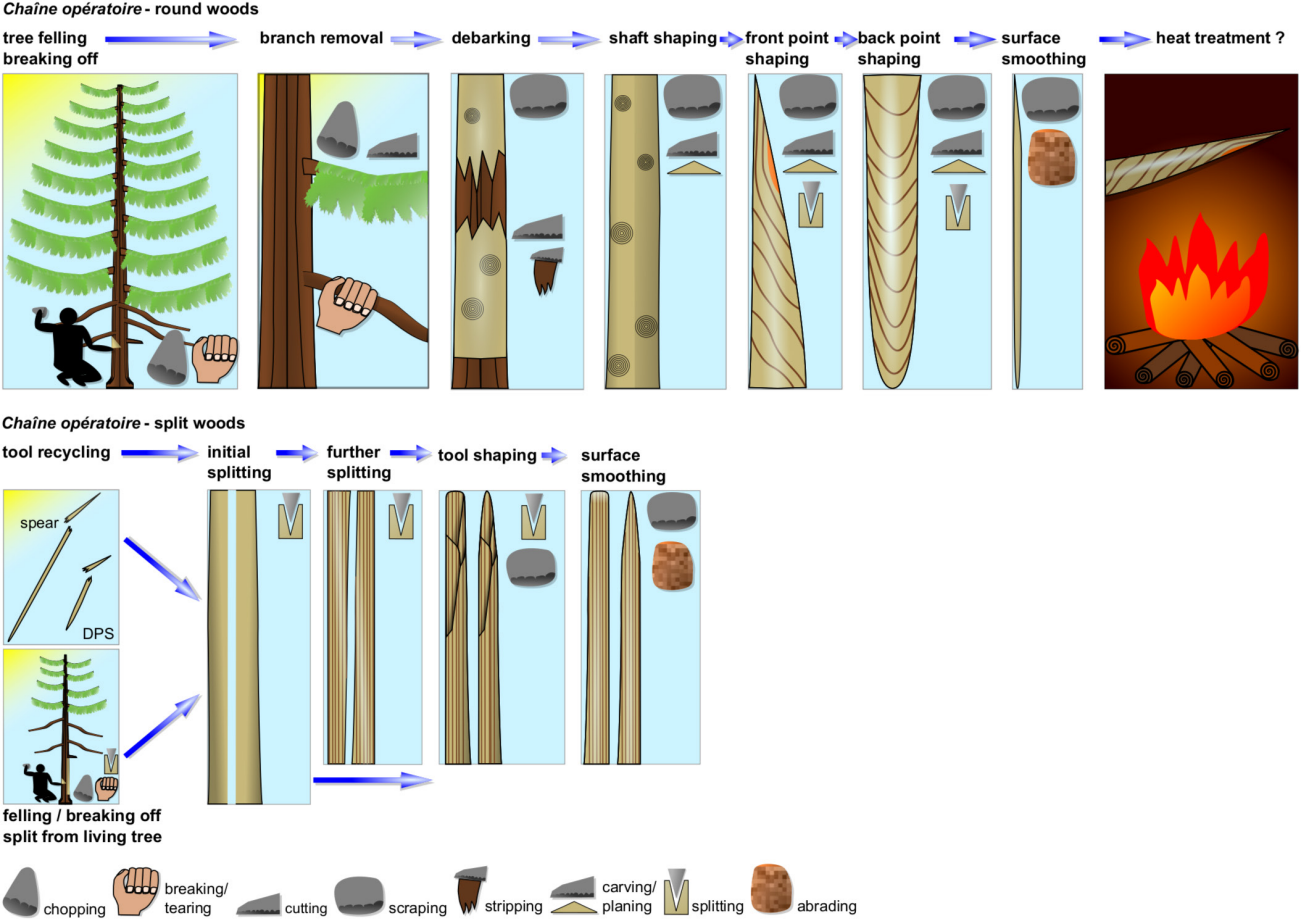
The two woodworking *chaîne opératoires* of Schöningen 13 II-4. (*Top*) ChO1—roundwood artifacts. (*Bottom*) ChO2—Split wood artifacts. Illustration: Dirk Leder, NLD.

Two distinct *chaîne opératoires* have been identified, one for the production of tools on roundwoods (ChO 1), usually hunting weapons, and one for the production of tools on split woods (ChO 2), that is domestic tools (Fig. 4).

ChO 1: A tree was selected to be fashioned into a spear or DPS (the DPS made on a branch is a singularity), it was broken off or chopped down, and branches were removed by chopping, breaking, and/or cutting. The bark was scraped off and/or peeled off assisted by incisions. The shaft was completely debarked and partially straightened by cutting deeper into the wood. The front point was placed offset (both points in DPSs), so that the pith exits along the side of the tip, while the pith remained in the center of the back point. Surfaces were smoothened probably for easier handling. The spears and DPSs display fine workmanship and intelligent tool design. They likely were introduced as finished tools to the site since working debris of the initial production stage is absent (bark/branches), while point fragments and traces of reworking demonstrate spears and DPSs were used and repaired on-site. They can be considered curated tools of personal gear used and maintained for as long as possible.

ChO 2: Split woods were almost exclusively made from spruce, however, using two different raw material sources. On the one hand, split woods frequently bear debarked surfaces with tool marks on them that are comparable to spears and DPSs suggesting that after a fatal break, spears and DPSs were recycled, deliberately splitting the wood in order to fashion tools (*SI Appendix, SI-Text*). Shaft fragments probably were recycled in such instances as they provide more volume than points. Some shaft fragments evidently have been further split longitudinally whereby one side might have been used to produce a split wood tool. On the other hand, DPSs on split woods, most pointed split woods, and few round-ended ones were shaped on knot-free, flawless wood. In such cases, the wood portion closest to the root of an old tree must have been selected and split laterally. Branches in this section of old trees are usually shed and overgrown while annual rings are more numerous herein. This wood material splits particularly well and in a controlled fashion. Trees might have been chopped down or split woods were directly split from the standing tree. Selected split woods were then longitudinally split and shaped into DPSs, pointed tools, and round-ended ones, using splitting, scraping and abrasion as preferred working techniques.

Working debris, mostly comprised of split woods, demonstrates that former wooden artifacts were recycled on-site and transformed into secondary tools (*SI Appendix, SI-Text*). Tools on recycled split woods were thus produced, used, repaired, and discarded on-site. They can be viewed as ad hoc or expedient tools, manufactured as needed and discarded shortly thereafter. In addition, flawless split woods made from old trees were introduced to the site for a premediated purpose that might be connected to the particular setting at the lakeshore.

**Fig. 5.**
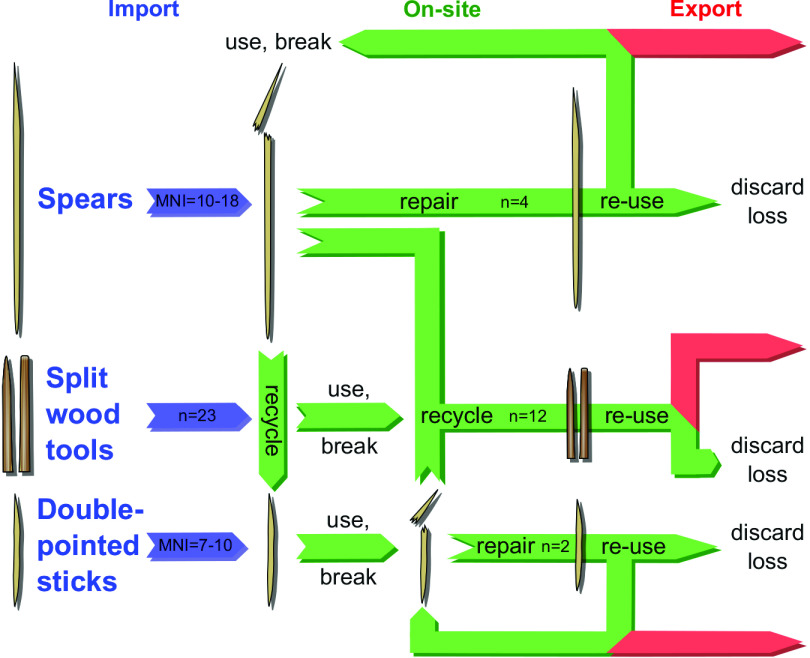
Lifecycle of wood artifacts at Schöningen 13 II-4. Import—use—repair—re-use—recycle—discard—export. Illustration Dirk Leder, NLD.

The evidence shows wood recycling played a pivotal role in the formation of the wooden artifact assemblage from Schöningen 13 II-4 (Fig. 5). Spears, DPSs, and selected split woods were imported as finished tools to the site from afar. Upon use and breakage on-site, they were repaired and/or recycled. Spears might have been recycled to shape DPSs; however, direct evidence for this is missing. Spears and/or DPSs were then split into multiple split blanks that were then shaped into pointed and round-ended tools. It is conceivable that some spears and DPSs were then taken away from the site, potentially after a stage of repair.

## Discussion

### Tool and Site Function.

Spears at Schöningen 13 II-4 were likely used as thrusting and throwing weapons in hunting small to large prey (11). However, one spear and one shaft fragment, each with a natural kink, would have been more suitable for thrusting rather than throwing. Although in theory, spears could have been used for fishing, the large size of most of the spears makes use as fishing implements less likely and evidence of spear fishing or fish consumption is generally sparse at sites predating the Upper Paleolithic (42, 43).

The DPSs likely functioned as throwing sticks in hunting medium-sized and potentially small-fast terrestrial prey as well as avian fauna, and according to ethnographic records, are tools that could be used by various members of the group including children (11).

Single-pointed split woods could have been used in either vegetal working or processing of hides. In support of this is the evidence that skinning was one of the major features of the butchery sequence at the site (32). Ethnographic comparisons are sparse, but among Selkup people in Siberia, 44 to 56 cm long and 1.7 to 6.2 cm wide single-pointed and double-pointed split woods were used to fry fish (44). Typical charring traces present on such items are however sparse on split wood tools from Schöningen 13 II-4 (n = 3). The split woods with a rounded tool end might have functioned as hide smoothers yet other functions are possible (41).

Although other wood resources would have been available near Schöningen’s former lakeshore in abundance (26), it is striking that spruce, spruce/larch and pine woods were deliberately selected to fashion tools on roundwoods and split woods from them even though they had to be brought to the site from afar. Such behavior evidences a clear raw material selection strategy likely connected to the physical properties (hardness, elasticity, weight) of these coniferous woods. When considering artifact types according to wood type, further patterns emerge. Spruce and spruce/larch artifacts clearly predominate over pine artifacts among (near-)complete tools such as spears, DPSs (ratio of 6:1), and split wood tools (ratio of 10.7:1) (*SI Appendix*, Table S5). Point and shaft fragments (ratio of 1.7:1) and working debris (ratio of 2.9:1) on the other hand comprise proportionately more pine artifacts meaning spruce and spruce/larch wood were preferred in shaping/recycling tools that were left on-site while recycled pine artifacts were more often exported from the site. The majority of the wooden artifacts thus evidences activities that revolve around the recycling of former tools while split woods and working debris demonstrate on-site production. Tools made on split woods were likely used in domestic rather than hunting activities. Spears, including spear fragments, and DPSs display tool maintenance activities carried out on-site.

Contrary to previous interpretations, the total number of 20 to 25 hunting weapons and 35 domestic tools demonstrates that Schöningen 13 II-4 functioned not only as a hunting/butchering site by a lakeshore (5, 9), but equally as a site for domestic activities. Such activities comprised wood tool curation, artifact recycling, on-site production of expedient wood tools, and use of these tools for varying purposes, including potentially hide preparation (*SI Appendix, SI-Text*). Differing weathering intensities observed on the wooden artifacts speak for a repeated use of the site by early hominins. The presence of 20 to 25 butchered herbivore carcasses equally support the notion of repeated site occupations and hunting events (32). Spatially overlapping and diverse on-site activities again point to repeated human occupations (*SI Appendix*, Figs. S18–S24) mostly during the summer/autumn season (45). In this context, it is fitting that trunks used to shape spears were felled in summer (7).

### Wood Artifacts and Human Evolution.

Schöningen is pivotal in understanding early hunting strategies, hominin range expansion, technical and social skills, and human cognition. Human brain size has increased over the past 2 Ma and combinations of ecological, social, and cultural factors have been proposed to account for it (46–51). The first phase of brain size increase between 2 and 1.5 Ma parallels the appearance of *Homo erectus* and the Acheulean technocomplex bringing forth more complex tool manufacturing concepts materialized in bifacial tools like handaxes. Early indirect evidence of hunting might be just as old (52). The second phase begins with the Middle Pleistocene at 780 ka and ends around 200 ka after the first appearance of *Homo sapiens* and *Homo neanderthalensis*. Hominin expansion into colder parts of Europe and the earliest evidence for cooperative hunting fall into this time slice (5, 9, 53, 54), which is paralleled by the appearance of organic tools (wood, bone, and antler) and the introduction of multi-component tools, i.e., hafting and production of adhesive materials.

With an age of c. 300,000 ka BP, Schöningen stands at the brink of the Lower and Middle Paleolithic and in the midst of the transitional phase from *H. heidelbergensis/H. erectus* to Neanderthals in Eurasia (55, 56). The deliberate raw material selection strategy and standardized tool production sequences observed in Schöningen 13 II-4 foreshadow trends commonly associated with the Middle Paleolithic when standardized lithic production concepts define this very period. The small and non-standardized flint tools from Schöningen are typical of the Central European Lower Paleolithic and many of them might have been hafted in wooden handles similar to those found at Schöningen 12 II, 12 B, and 13 DB (24, 57, 58). The wooden tools from Schöningen thus evidence technological complexity and standardization already during the Middle Pleistocene.

**Fig. 6.**
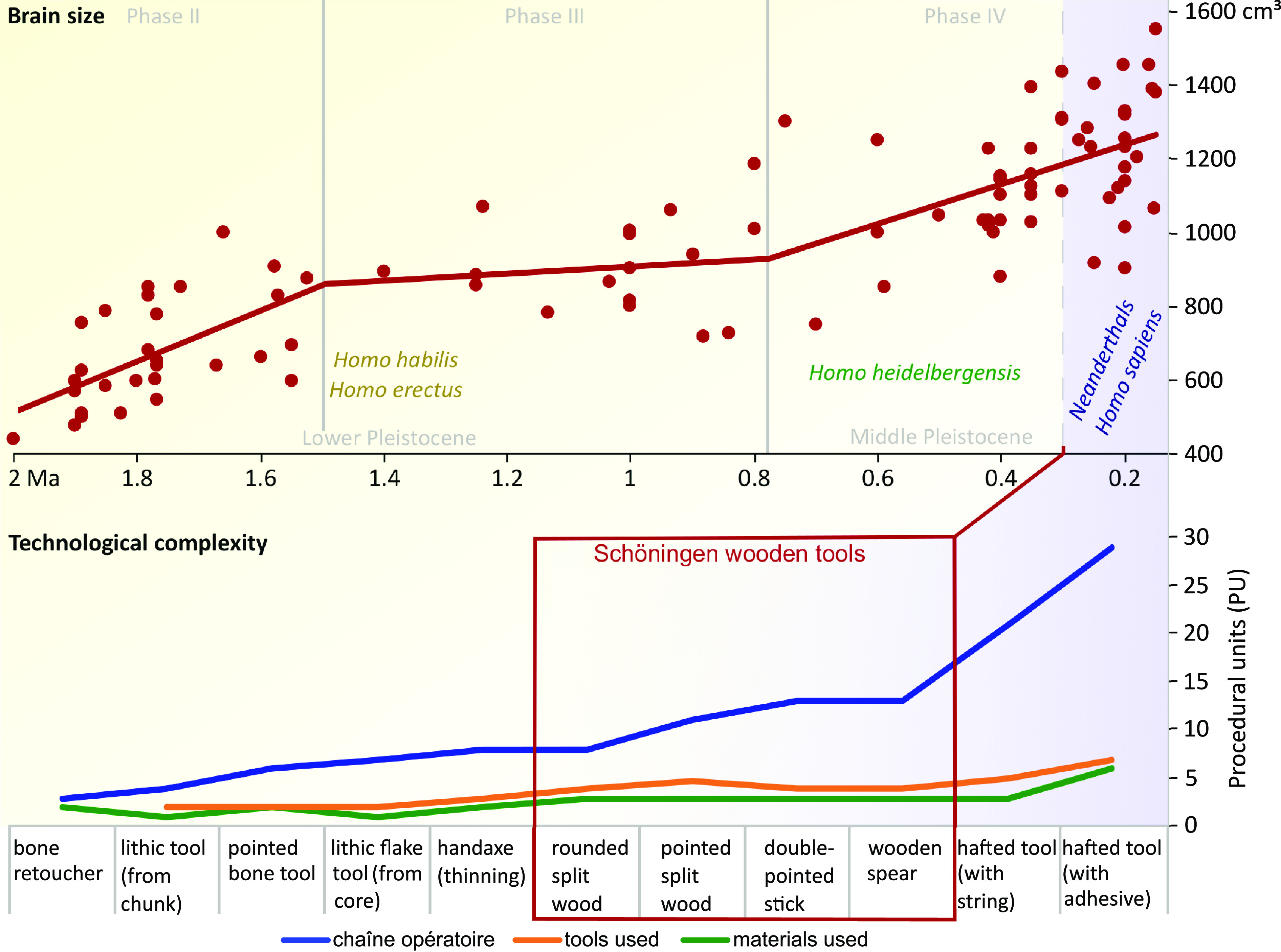
Human brain size evolution and technological complexity development during the Early and Middle Pleistocene. Brain size data after Gingerich (46). Technological complexity according to multiple sources (*SI Appendix*, *SI-Text* and Table S23). Procedural units after Perreault et al. (61) (*SI Appendix, SI-Text*). Illustration Dirk Leder, NLD.

According to a predictive model, hominin brain size evolution is best explained when individuals face a combination of 60% ecological, 30% cooperative, and 10% between-group competitive challenges (47). Ecological challenges are often met with technological improvement as part of a risk buffering strategy (59–61). The transfer of technological knowledge from one individual to another requires learning and memorizing successive steps in tool production (61–64). The more complex a tool, the more steps and quality controls to memorize. The Early and Middle Pleistocene archeological record shows an evolutionary trend of increasing technological complexity beginning with simple flake tools followed by handaxes, then sophisticated wooden hunting weapons, and finally hafted tools (Fig. 6 and *SI Appendix*, *SI-Text* and Table S23). Increasing technological complexity has been interpreted as a proxy of cognitive abilities (62, 65, 66) and increasing reliance on social learning in *Homo* (61, 63, 67).

Schöningen’s wooden artifacts play a key role in understanding early organic technologies. Hunting weapons were not simple sticks with points, but technologically advanced tools. The selected raw material was particularly suitable and the best option at the end of the interglacial due to its growth conditions providing hardness and elasticity at the same time. It was not available at the site but had to be procured elsewhere, which requires anticipation of an arising need and thus planning depth. The design of the spears (e.g., offset front point, point of balance) made them durable thrusting and throwing weapons. DPSs on the other hand served as long-distance weapons for smaller prey-classes that could have been successfully used for hunting by nearly all but the youngest members of the group. Consequently, spears and DPSs can be viewed as two elements of a complementary hunting tool kit. Earlier and later wooden spears from Clacton-on-Sea and Lehringen bear similar technological features (6, 8, 29) and despite the time gaps, these wooden spears may elucidate the successful knowledge transfer over many generations during the Pleistocene. Assuming that woodworking technologies have been present for as long as lithic and bone technologies have, the preservation of wooden tools clearly affects our understanding of technological complexity at any given time, meaning where wooden artifacts are not preserved, we might underestimate the cognitive abilities of prehistoric societies.

The cooperative dimension in the aforementioned model includes human group hunting, imposing the challenge of strategic planning, coordination, and situational adaption (68, 69). Brain growth significantly increased during the Middle Pleistocene (46) connected to food diversification and reliable access to animal food sources (70, 71). Hunting in this context ensures sustainable access to high-energy, high-quality food with proteins and fatty acids being essential building blocks of the human brain. Hunting thus plays an essential role in the physical and cognitive evolution of hominins. Changing hominin subsistence strategies during the Pleistocene then favor frequent and systematic hunting in open environments with access to large animals (48). The complex interplay of planning depth, social interaction, technological complexity, and secure food supply over many generations thus made a decisive contribution to the successful expansion of *Homo* from Africa to Eurasia and its persistence throughout the Pleistocene.

In this context, Schöningen evidences successful hunting by the presence of 20 to 25 butchered animal carcases, mostly horse, and the presence of 20 to 25 wooden hunting weapons. Hominins at the site were thus able to ensure primary access to high-quality food sources already 300,000 y ago. Hunting is probably much older and primary access to high-quality food sources over generations would have benefited brain growth and human socio-cognitive abilities. Likewise, it would have ensured sustainable populations even in less favorable parts of Europe during the Pleistocene and contributed to human range expansion across the globe. Schöningen’s wooden hunting weapons are thus an excellent ambassador of these important milestones in human evolution.

## Materials and Methods

### Wood Assemblage.

The wooden assemblage analyzed in this study involves all items from Schöningen 13 II-4, the Spear Horizon that was completely excavated until 2008. It consists of natural and worked woods, i.e., 744 find IDs comprising 365 single finds and 369 bulk finds (*SI Appendix*, Table S1). Forty-six artifact IDs could be refitted to 16 objects including eight formerly published spears (spears I–VII, and X) (*SI Appendix*, Table S2).

### Species Identification and Artifact Categories.

Wood specialists Werner Schoch (*Langnau, Switzerland*) and one of the authors (M.S.) identified the wood species following standard botanical methods (*SI Appendix, SI-Text*). Wood objects are subdivided into four artifact categories according to the frequency and clarity of tool marks. Category 1 artifacts bear definitive working traces. Different tool mark types are present on a single item and often more than once per type. Category 2 artifacts bear multiple probable working traces, sometimes less clear due to preservation, which occur in various combinations. Category 3 artifacts bear possible working traces in combinations of at least two different tool mark types. Category 4 items lack diagnostic tool marks often due to an advanced taphonomic deterioration of surfaces.

### Terminology and *Chaîne Opératoire*.

The terminology used in this study follows a recently developed glossary on wooden artifacts of stone tool using cultures (72). Accordingly, our study follows the *chaîne opératoire* concept. Phase 0 describes the selected raw material. Phase 1 describes the shaping process. Phase 2 describes the use, re-working, and discard of the artifact. In phase 3, taphonomic impacts are evaluated. Phase 4 deals with traces caused during the excavation and after conservation. Based on descriptions in the literature, we calculated procedural units, i.e., manufacturing steps that contribute the finished form of a technology, after Perreault et al. (61) for relevant Early and Middle Pleistocene technologies (*SI Appendix, SI-Text*). The concept follows the logic of the *chaîne opératoire* in line with our approach concerning woodworking technology at Schöningen. We expanded the concept including raw material acquisition and transport before the tool manufacturing stage as well as tool use and tool discard after the manufacturing stage.

### Minimum Number of individual (MNI) Hunting Weapons.

Spears and DPSs are preserved as complete tools as well as in fragments. In addition, point and shaft fragments are present too, leading us to calculate a minimum number of individual hunting weapons proposing two different scenarios. One model assumes all point and shaft fragments belong to spears (SpearMax), while the other one assumes all point and shaft fragments belong to DPSs (DPSMax) and various limiting factors are considered (*SI Appendix, SI-Text*).

### Morphometrics.

Morphometric analysis serves two purposes, first to quantitatively discriminate between different artifact types and second to evaluate the degree of standardization of each artifact type. Sediment compression had affected wooden items resulting in oval cross-sections. Consequently, the diameter of all roundwoods, i.e., spears, throwing sticks, point, and shaft fragments, respectively, was calculated by adding the width and thickness measures at any given location and averaging the result. Morphometric data include the degree of tapering, e.g., in spear points. Tapering (Tp) is the change in diameter (d) per distance length (l). The diameter at location 2 (d_2_) is subtracted from the diameter at location 1 (d_1_), the difference between (d_1_) and (d_2_) is divided by the length (l) of the distance between d_1_ and d_2_, $Tp=(d_{1}-d_{2})/l$. Measurement techniques followed those described previously (11). Statistics were calculated in PAST v. 4.14 (73).

### Visualization Techniques.

All objects were photographed with a Nikon D850 (45.7 MP) and stitched with Adobe Photoshop. A professional photographer (MINKUSIMAGES) made overview photographs of selected objects using a Nikon Z7 II (45.7 MP) with stacking function and stitched the images using Capture One 21. Besides macroscopic observations with the naked eye, macro-photographs of traces were made with a Nikon D7000 (16 MP). Detailed images were captured using a stereomicroscope (Leica S9D with a Flexacam C3 camera). Selected traces have been recorded with the 3D structured light microscope Keyence VHX5000. All traces have been recorded according to their position on the item (compare 11) and a complete catalog of the worked items is currently in preparation (74). µCT scanning was performed by Waygate Technologies using a Phoenix Vǀtomeǀx M300 and served to evaluate annual ring sequences (*SI Appendix, SI-Text*).

## Supplementary Material

Appendix 01 (PDF)

## Data Availability

All study data are included in the article and/or *SI Appendix*.
