# Supplementary material for: The wooden artifacts from Schöningen’s Spear Horizon and their place in human evolution
Source: Proc Natl Acad Sci U S A. 2024 Apr 1;121(15):e2320484121. doi: 10.1073/pnas.2320484121 (PMC11009636; doi:10.1073/pnas.2320484121)
Supplement: Supplementary file 1 — Appendix 01 (PDF) [file pnas.2320484121.sapp.pdf]

Supporting Information for

The wooden artefacts from Schöningen's Spear Horizon, and their place in human evolution

Dirk Leder, Jens Lehmann, Annemieke Milks, Tim Koddenberg, Michael Sietz, Matthias Vogel, Utz Böhner, Thomas Terberger

Corresponding authors
Dirk Leder, Email: dirk.leder@nld.niedersachsen.de
Thomas Terberger, Email: thomas.terberger@nld.niedersachsen.de

This PDF file includes:

- Supporting text
- SI References
- Figures S1 to S24
- Tables S1 to S24

Supporting Information Text

Introduction

Palaeolithic wooden tools

The earliest indirect evidence for human woodworking dates back 2–1.5 Ma and is based on use-wear analyses on lithics (1, 2). The earliest evidence of worked wood comes from the Acheulian site of Gesher Benot Ya'aqov in Israel entailing a possibly worked log and a plank fragment. Based on radiometric dating this site is about 780 ka old (3). A possible wooden building structure has been reported from Kalambo Falls in Zambia and is c. 500 ka old (4).

The discovery of early wooden hunting weapons, such as spears and throwing sticks, have revolutionised our understanding of early human hunting abilities, social interaction, and hominin cognition (5, 6). The earliest evidence for the usage of wooden hunting spears probably comes from the English site Clacton-on-Sea that yielded a single spear point fragment made from yew (7). The find has been discovered at a coastal cliff more than 100 years ago in layers that based on geological correlations supposedly are about 400 ka old. Bilzingsleben (400-300 ka) and Bad Cannstatt (300-125 ka) in Germany also delivered wooden remains, whereof some were interpreted as spears (8, 9). However, due to their poor preservation, their artificial character could not be assessed with certainty. The well-known wooden spears from Schöningen, Germany, are c. 300,000 years old (5). Another spear made from yew wood was discovered at the German site Lehringen where it was found associated with the carcass of a straight-tusked elephant (10). Based on pollen data this complete spear dates to the last interglacial and is thus 130-115 ka old. From the river site of Ljubljanica River in Slovenia an alleged wooden point fragment made from yew has been reported and was directly dated by radiocarbon yielding two dates around 40 ka cal BP (11).

Further possible wooden spear fragments, about 13 ka old, come from Monte Verde in Chile (12). Other Palaeolithic hunting weapons come from the Kalambo Falls where a wooden club and digging sticks were discovered (13). Based on techno-typological features of accompanying lithics and radiometric dating, these are among the oldest known wooden tools dating to c. 500-250 ka. Two throwing sticks made from spruce have been reported from Schöningen (14, 15). At Florisbad in South Africa, a fragment of a throwing stick made from kundanyoka knobwood was discovered and radiometric dating places the find between 260 and 125 ka (16).

The earliest known wooden arrows come from Stellmoor in Germany (17). The majority of the more than 100 composite arrows, consisting of a wooden shaft, a wooden fore shaft, and a stone tip, were however destroyed in WWII. The accompanying lithic finds and radiocarbon dates on faunal remains securely place them within an Ahrensburgian context, i.e. 13-12 ka cal BP.

Digging sticks that were probably used to retrieve underground storage organs (e.g. tubers, bulbs, roots) are prominently represented at sites in Africa and Eurasia. The Chinese site Gantangqing yielded 35 wooden items, most of them interpreted as digging sticks, while there also are five items with a 'hook' that presumably served in slicing tubers, and a single item that might have been used

as an awl (18). The site has been radiometrically dated to about 300 ka. Poggetti Vecchi in Italy has produced another large assemblage of wood remains (19). Of the 58 wooden items 39 have been identified as digging sticks and fire was used to improve the workability of these very hard boxwood branches. The site has been radiometrically dated to 170 ka. A single digging stick and another wooden object have been recovered from the site of Aranbaltza III in Spain (20). The digging stick wad made from yew and the associated layer is radiometrically dated to 70 ka. Altogether 14 wooden remains including a digging stick and poison applicator come from multiple layers at Border Cave in South Africa (21). The items were most likely made from white-berry bush. The various layers containing the wooden objects date between 40 and 24 ka based on radiometric dating. Finally, 13 ka old digging sticks have been discovered at Monte Verde, Chile (12).

Domestic wooden tools are comparatively rare in the Palaeolithic record. About 30 socketed handles, so-called clamp shafts have been reported from various Middle Pleistocene sites at Schöningen (22). Further domestic tools might be present in the form of vessels at Abric Romani in Spain dating 49-45 ka BP (23). Ohalo II in Israel has delivered eight wooden objects, including a bark plank, pencil-shaped items, and incised objects that might bear symbolic meaning dating 22.5-23.5 ka cal BP (24). Wooden mortars and tent posts have been reported from Monte Verde (12). Finally, the Late Glacial Shigir idol in Russia was made from larch and directly dated by radiocarbon sculpture (25). The artefact represents the earliest known monumental sculpture.

Materials and Methods

Wood species identification

The identification of wood species follows established botanical methods. Tree ring sequences and analysis were reconstructed with the use of micro-cuts and micro-CT scans. The microcuts were documented with a digital microscope (LEICA M125c reflected light microscope, planapochromatic objectives 0.63x and 1.6x), MC170 HD CAMERA, CLS 150 LED ring light and LEICA-LAS software v4. 12.0. These approaches enable an estimation of the minimum age of the branch and thus tree, the identification of growth characteristics, and other features such as compression wood. In addition, natural material and standard reference texts (e.g. 26) were consulted for interpreting natural features. Distinguishing between *Picea sp*. and *Larix sp.* can be challenging while two attribute are commonly used.

1) Sharp break or continuous transition from early to late wood. This feature is difficult to determine, especially in the densely grown woods at Schöningen and can thus hardly be used for making reliable distinctions.
2) Arrangement of the bordered pits in the tracheid walls. Double rows of bordered pits are rare in *Picea* but common in *Larix*. Numerous double rows of bordered pits speak for the presence of *Larix*, which is rare in Schöningen. When double rows are rare or not present at all, everything speaks in favour of *Picea* and a distinction can be made with high probability.

μCt scans

Micro-Computed Tomography (micro-CT) provides a non-invasive means of evaluating the raw material features, alongside a clear way of analysing the manufacturing techniques and natural and taphonomic alterations. X-ray micro-CT scans of the entirety of a double-pointed stick (ID 1779) were performed by Waygate Technologies GmbH with the micro-CT scanner phoenix V|tome|xm. The object was scanned in sections every 5 cm, as well as, at 1 cm from each tip. The acquired micro-CT data were processed and imaged using VGSTUDIO MAX 3.3.4 software at the NLD and with the Avizo software (FEI, Thermo Fisher Scientific, Hillsboro, Oregon, USA) at the Department of Wood Biology and Wood Products, University of Göttingen.

ATR-FTIR analysis

ATR-FTIR analysis was performed on a single point of a DPS (ID 1779). We selected View A2 on Point 1, concentrating on seven darker areas near the point, and three visually lighter coloured areas near the centre of the artefact. Apart from structural imaging, chemical analysis of a selected dark patch were performed using non-destructive Attenuated total reflection Fourier-transform infrared spectroscopy (ATR-FTIR) to understand whether these areas are residues could have been formed by mould or by deliberate charring. FTIR was selected based on previous application of this wood and wood components (27–31) and charring (32–34).

Spatial distribution (GIS)

Spatial distribution pattern of the analysed wood objects were undertaken using QGIS 3.28. Kernel density analyses was used to achieve density raster files. In point distribution maps, the centroid of IDs was used.

Results

Hunting weapons

Spears

Eight artefacts are securely identified as spears, three are complete and five fragmented. All spears belong to artefact category 1 (Table S6). Complete spears reach lengths between 1.8 and 2.5 m (35), while all spears have diameters between 2.3 and 4.5 cm (median= 3.5 cm).

The *chaîne opératoire* can be described as follows. Seven spears were manufactured from spruce trunks, while a single spear was manufactured from a pine trunk. The spears have 18–60 very dense annual growth rings (median= 51) providing hardness and elasticity in these softwoods at the same time (26, 25). The pine spear has the fewest annual rings. All spears show moderate growth ring widths at the beginning while after 6-15 years, annual rings become increasingly thinner speaking

for deteriorating growth conditions. While five of them evidence frostbite during spring (Spear I, II, III, and V) a single spear bears indications of a summer draught (Spear VII). Natural flaws like callus tissue, insect feeding traces, or even natural kinks, which can be expected to weaken the wood or at least to affect the handling of the items, did not hinder humans to select such specimens anyway (Table S6). This might point to the high value these raw materials might have had and natural flaws were thus acceptable.

Trees were broken off or perhaps chopped down during the summer months (35) likely with simple chopping tools given the absence of handaxes from the Central European archaeological record at this time (36, 37). So far, however, no reliable chopping marks have been identified in the wood collection of the spear horizon. Branch knots were usually removed by cutting or tearing or a combination of both, while in rare incidences knots were removed by chopping. Knots at the front point are flush with the surface while along the shaft and the back point they can be either flush or protruding. Spears are usually completely debarked, while a thin layer of cambium partially covers the shaft of two spears (Table S6). With cambium attached, the underlying wood retains moisture making the artefacts more durable and preventing it from warping (26). Inner bark was scraped off with a retouched stone tool as long-parallel striations on the cambium suggest (4, 38). Debarked wood surfaces bear oblique cut marks connected to the stripping off of the bark (39). Further tool marks like scraping marks, surface facets and tool facets evidence a range of techniques employed in the debarking and shaping of the spears including scraping and carving/planing (Table S6; Figs. S2-S3; 40).

The tree's growth direction is determined by the growth direction of the branches that point upwards in living trees. Accordingly, front points are oriented toward the base of the tree, whereas back points are oriented toward the treetop. As front points are oriented toward the base of the tree, i.e. toward its roots where more tree rings are present, this makes them more robust (26). The pith imposes a weak point, so placing the point off-centre further contributes to the durability of the point (Biermann 35, 41). Tapering was achieved by working deeper into the wood from the point to the shaft as stop marks indicate and annual ring surfaces were created this way. Front points taper at similar rates (0.06–0.09 Tp) as back points (0.05–0.11 Tp), however, they taper over a longer distance (40.0 cm vs 20.0 cm) and thus appear more slender or "pointier". The front points are very smooth compared to the shaft and were scraped and probably abraded using stone, grass, or other abrasive materials. Longitudinal crushing as a result of impact is present on the front point, the back point, or both (n= 6; Table S6). At the front point this likely results from impact on the target or on the ground when a thrown spear missed its target, whereas in back points, crushing more likely results from spears being used as walking sticks or when hunters leaned on them. Splinter negatives with rounded edges on back points (Spear I, III, IV, and VI) indicate such behaviour. Dark discoloration on the front and back points respectively might be connected to heating in an attempt to dry and harden them (42), while residues like blood or grease may have caused the discoloration instead (see FTIR analysis below). Other possibilities include contact with soil when a thrown spear missed its target (front point)

or the spear was rested on the ground (back point). A clear identification of the cause of discoloration has not yet been achieved.

Shaft breaks present in six spears evidence use-damage by bending stress likely resulting from high velocity impact (40, 43). Long fibres and uneven break surfaces show these breaks must have occurred when the wood was still relatively fresh and wood cells have not yet deteriorated. However, a shaft break soon after the deposition cannot be excluded and is quite possible in cases where such fragments were excavated close to each other (Spear I, V, and X). In other cases fragments that would attach to shaft breaks are missing and these spears are incomplete (Spear II and IV, VII, X).

Two items show evidence of reworking. In both cases, front points were reworked by removing a series of splinters from the point toward the shaft, followed by smoothing (Table S6; Fig. S4). The point was thus roughly reshaped probably after surface splintering or breakage of the former point occurred.

Taphonomic traces such as surface weathering and sediment erosion have been observed on all items and bacterial decay likely lead to the softening of the wood (44, 45). More invasive surface alterations occur in the form of surface damages on all specimens, longitudinal cracks in some, while delamination, and trampling marks are common too (Table S6). Superimposed sediments compressed all items evidenced by their oval cross-sections and longitudinal compression cracks. Taphonomic transverse breaks indicate the lateral movement of spear fragments after deposition and of sediment pressure (Fig. S5). Excavation and post-excavation alterations have been observed on all items in the form of cuts and compressions caused by the excavation while taphonomically induced transverse cracks deepened after the dry-conservation.

Double-pointed sticks (DPS)

The first wooden tool ever discovered at Schöningen 13 II-4 was indeed a double-pointed stick (ID 1779) interpreted as a throwing stick (5) and recently analysed in detail by our research group (15). Another specimen (ID 28108) was excavated some 116 m southeast of it in 2016 and was not part of this study (14). However, this incomplete item matches the typical features of a DPS. Five further DPSs have been identified in this study including a re-worked artefact with a charred point, the co-called *Bratspieß* or skewer (5, 35), and two long and slender DPSs worked on split woods. All items belong to artefact category 1 (Table S7).

The six DPSs/ DPS-fragments analysed in this study range in length from 45 to 88 cm (median= 64.5 cm) while maximum diameters are between 1.7 and 3.0 cm (median= 2.4 cm). The longest item is incomplete and might have been about a metre long. Two DPSs are intact while the others have points missing. The greatest diameter of the two complete items is located at about 2/3 of length (60.9% and 66.7% of length).

Four DPSs are made from spruce, one from spruce/larch, and one from pine. Tree trunks were used in three specimens, a branch in another, while two long split woods were shaped into DPSs too.

Annual rings sequences have been analysed on a single item thus far (ID 1779). This DPS has 63 annual rings preserved and thus more than any of the spears (Fig. S6; 15). However, the item shows a similar growth pattern. After the first 8 years of moderate growth conditions fluctuate until about 17 years whereupon growth stabilises on a much lower level speaking for deteriorating conditions. A dry summer season was detected at 10 years. While the DPS worked from a branch has a slight natural curvature, other natural flaws are absent from the DPSs speaking for a thorough selection of the raw material.

The *chaîne opératoire* of the DPSs made on roundwoods is essentially the same as that of the spears while also bearing comparable tool marks (see Spears above; Figs. S2-S3). Contrastingly, DPSs on split woods were shaped by splitting with subsequent smoothing by scraping and abrasion (Table S7). Unlike the spears, the DPSs are shaped with two offset points with the pith exiting on the side. Points taper with 0.03–0.13 Tp (median= 0.6 Tp) and tapering extends over 15–50 cm.

Two incomplete DPSs have one reworked point each shaped either by splitting followed by smoothing or by carving/planing and splitting (Table S7; Figs. S7-S8). This rather crude treatment in comparison to the fine workmanship observed on spears and the DPSs might be explained by the need for a quick repair during the hunting. Use-wear on the DPSs is present on all preserved points in the form of longitudinal crushing. Two objects bear shaft breaks that lead to the loss of a point while one DPS has a bevelled fracture resulting from acute bending stress (Table S7).

Traces of fire exposure have been detected on the reworked point of the “skewer” (Figs. S7-S8). The discoloration is not very deep as later tip damage shows, the surface lacks cracks while working traces are still well-visible. This probably speaks for a short exposure to fire as would be expected if the aim was to dry or harden the point (42) and given the absence of actual charring, usage e.g. as a torch or roasting skewer can be excluded. Dark grey discolorations are present on the points of two items that might be heating traces, residue like blood or grease, or result from contact with sediment, e.g. when the DPS was thrown and missed the target. An FTIR analysis on one DPS (ID 1779) was inconclusive (15). The ATR-FTIR measurements of the throwing stick show wide variation of the spectra obtained and therefore all measurements of light and dark spots respectively were averaged (Fig. S9). The embedding agent, Kauramin 800 (differentiating absorption at 909 cm-1), influences all spectra of the throwing stick. The results show that IR-spectra of light and dark measurement spots are very similar. The very few differences in absorptions between dark and light areas, (e.g. 1718 cm-1, 1644 cm-1, and 1213 cm-1), are within the width of standard deviation of measurement points and hence are not significant. It was not possible to detect changes on the wood surfaces that resulted from exposure in fire. This is likely due to a number of confounding factors including that wood spectra are a mixture of the main components lignin and (hemi-)cellulose, and growth rings result in varying proportions of these components and in a resulting modulation of the spectra. Furthermore, the embedded resin varies in thickness, which superimposed a background on all spectra of the wood. The unexposed wood component lignin is aromatic, and the embedded Kauramin resin adds aromaticity via its triazine ring (46). Thus the charring is not

identifiable qualitatively by a new aromaticity peak from graphene (graphene aromaticity peak at for instance 1610 cm; 47) but instead only can be identified quantitatively. This also applies to oxidation-induced carbonyl functions, as both lignin (27) and Kauramin (46) introduce carbonyl functions. As the changes in IR spectra for the dark zones compared to light ones are quantitative rather than qualitative, the broad local variation of IR spectra on the double-pointed stick did not allow us to differentiate chemically between visually light and dark regions.

Further dark discoloration has been observed along the shaft of a 60 cm long item in the form of five faint rings, each ca. 1-3 cm wide (ID 7172). This feature is unique so far and currently difficult to interpret. Taphonomic traces are present on all DPSs (Table S7). Surface weathering and sediment erosion have been observed on all items and again bacterial decay likely lead to the softening of the wood. More invasive surface alterations occur in the form of surface damages, cracks and flaked surfaces as well as and trampling marks. All items are compressed by superimposed sediments as evidenced by their oval cross-sections and longitudinal compression cracks. Transverse surface breaks evidence the lateral movement of DPSs after deposition.

Finally, excavation and post-excavation alterations have been observed on all items, mostly in the form of transverse break surfaces, i.e. items fragmented during excavation, and transverse cracks that expanded after conservation. Localised compression and puncture were observed in one case.

Point fragments

The assemblage contains 17 point fragments, 14 belong to wood artefact category 1, two to category 2 and a single item to category 3 (Table S8). The point fragments are made of spruce in 13 cases and of pine in four. Lengths vary between 3.1 cm and 56.7 cm with a median value of 15.7 cm while the maximum diameter lies between 1.0 and 2.8 cm (median= 1.8 cm). Eleven items have a preserved tip and given the incomplete state of preservation, tapering varies considerably between point fragments with a taper between 0.04 and 0.13 cm (median = 0.07 cm) and over 4.5 to 30 cm length (median = 12.9 cm).

Six objects were clearly worked from trunks as branch whorls and seams indicate, while in ten specimens, the blank type might have been either a branch or a trunk (Table S8). Assuming all point fragments may potentially be spear points, the distinction between front point and back point rests upon observations made on the Schöningen spears and therefore on two criteria. Firstly, the location of the pith and secondly, the growth direction of the tree (see Spears above). Accordingly, the assemblage contains ten front points, four back points and three front or back points. Front points and front/back points might belong to either spears or DPSs, while back points can only be parts of spears (see MNI hunting weapons below).

The *chaîne opératoire* in point fragments is comparable to that of spears and DPSs (see Spears and DPSs above). Natural flaws like callus tissue, insect feeding traces or kinks are absent from the points, which compares well to the spear and DPS points. After chopping down or breaking off the tree, branch knots are removed either by cutting or breaking them off or by a combination of both. In

rare instances, knots were chopped off. Knots at the front points are usually flush with the surface for at least 10 cm from the very tip. Contrastingly, knots further down the shaft and at the back point can be flush or (slightly) protruding.

As seen in the Schöningen spears that are usually completely debarked, cut marks are absent from areas where wood material is more intently removed and annual ring surfaces show. As expected, cut marks have been observed on the point fragments only beyond 10 cm from the tip among eight longer items. Besides these oblique cut marks, numerous other tool marks like scraping marks, surfaces facets and tool facets evidence a range of techniques employed in the debarking and shaping the items including scraping and carving/planing (Figs. S2-S3).

Use-wear on point fragments is affirmed in a number of ways including longitudinal crushing that is the compression of wood surfaces upon impact. Longitudinal crushing has been confirmed in six spruce points, while two further spruce and pine points respectively potentially bear such impact marks (Table S8). These include four front points, four back points and two front or back points. Crushing in back points likely originates from the spear resting on the ground or being used as a walking stick, whereas crushing at the front point should result from impact on the prey's bones or impact on the ground when a thrown spear missed its target.

Tips that likely broke off during use are relatively rare with only four confirmed specimens, twice made on spruce and pine respectively (Table S8). The others might either have been removed by taphonomic processes. Contrastingly, shaft breaks have been observed among 11 items. Confirmed shaft breaks are present in eight spruce points while potential breaks are present on a further spruce and two pine artefacts. Long fibres and uneven break surfaces show these breaks must have occurred when the wood was still relatively fresh and had not deteriorated yet. While a taphonomic shaft break soon after the deposition cannot be excluded, one would assume the shaft would be found nearby and could be refitted. This was not the case among any of the point fragments, and therefore, the breaks are most likely from use. Finally, dark discoloration has been observed along the shaft of a 60 cm long back point (ID 7172). Discoloration herein takes the form of five faint zones, each ca. 1-3 cm wide, that go around the entire circumference of the point. This feature is unique so far and currently difficult to explain.

Reworking of point fragments is clearly visible in only two cases (Table S8). It is particularly evident in a front point (ID 14852) where the point was essentially split in half by a single bevel cut that stopped at a protruding knot, followed by intent smoothing (Fig. S10). Unlike in other spear points, discoloration seems absent though. In the second front point (ID 4236) the originally worked and smoothened surface is truncated by multiple carving/planing marks altering the taper of this point (Fig. S3g). This rather crude re-working technique when compared to the fine workmanship observed in spears and DPSs might imply a stage of repair carried out in a rush, possibly the hunt.

Taphonomic traces are present on all point fragments (Table S8). Surface weathering and sediment erosion have been observed on all items and bacterial decay again likely lead to the softening of the wood. More invasive surface alterations occur in the form of surface damages on 12 artefacts, cracks

and flaked surfaces are frequent, whereas trampling marks occur in only two fragments. A single item bears traces of rot and fungal infestation (ID 8674). All items are compressed by superimposed sediments as evidenced by their oval cross-sections and longitudinal compression cracks. Transverse surface breaks seem present on all items apart from two (ID 4236 and 8689).

Finally, excavation damage has been observed among six items in the form of transverse break surfaces, i.e. items fragmented during excavation. In two cases oblique striations are also the result of the excavation, as is localised compression on one item.

Shaft fragments

The assemblage contains 13 shaft fragments, eight belong to wood artefact category 1, one to category 2 and four to category 3 (Table S9). The shaft fragments are made from spruce in five cases, from spruce/larch in one, and from pine in seven cases. Lengths vary between 12.0 cm and 82.8 cm with a median value of 19.3 cm and a maximum diameter between 1.8 and 3.2 cm (median= 2.4 cm). The maximum diameter was calculated from six items with a completely preserved circumference. The seven other fragments are split in half, so the maximum widths was calculated instead yielding a range of 2.3 to 3.5 cm (median= 2.5 cm), which is comparable to the items with a complete circumference. Split fragments are between 0.8 and 1.8 cm thick (median= 1.3 cm). It thus appears that roundwoods with a diameter greater 2.3 cm are preferred in splitting. Width and diameter values of shaft fragments correspond to slender spears (diameter= 2.3–3.5 cm; Table S6) and to double pointed sticks made on roundwood alike (diameter= 2.1–3.0 cm; Table S7).

The *chaîne opératoire* of the shaft fragments compares well to that observed in spears and DPSs (see chapters above). Tree trunks have been selected in eight cases (5x pine, 3x spruce), whereas in the five remaining cases either a branch or a trunk was used (2x pine, 2x spruce, 1x spruce/larch) meaning clear usage of branches cannot be ascertained (Table S9). A single thin cut has been analysed thus far (ID 11383). Seventeen annual rings have been detected beginning beneath the cambium with ca. 3-4 rings missing to the pith. After the 5th year in this sequence, annual rings become thinner speaking for slower growth conditions over a period of more than ten years. The presence of natural flaws like callus tissue, insect feeding traces, or even natural kinks on shaft fragments, which might be expected to weaken the wood or at least to impact the handling of the item, did not hinder humans to select such specimens anyway.

After chopping down or breaking off the tree, branch knots are removed either by cutting or breaking them off, or by a combination of both. Chopping marks perpendicular to the knots' surfaces are potentially present on a single item. Knots along the shaft can be flush or (slightly) protruding.

The majority of the shaft fragments are completely debarked (n= 9; Table S9) while two items have retained smaller portions of cambium, whereas two others bear substantial cambium coverage (IDs 7107_7108 and 11383_12886). Where cambium is substantial, it is covered in long-parallel striations that might be indicative of the scraping off of the inner bark using retouched stone tools (4, 38). Debarked areas also bear numerous oblique cut marks that might be connected to the stripping off

inner bark (39). Besides these oblique striations, numerous other tool marks like scraping marks, surfaces facets and tool facets evidence a range of techniques employed in the debarking and shaping of the items including scraping and carving/planing (Figs. S2-S3).

Use-wear is evidenced in the form of shaft breaks that are clearly present in three cases and potentially so in another five (Table S9). These include three spruce fragments, four pine fragments and one spruce/larch fragment. Shaft breaks on these items confirm their use while other break surfaces are taphonomic in nature. A single item might have a partially charred surface (ID 11979).

Reworking of shaft fragments is evidenced in a number of ways but mostly connected to intentional splitting (Table S9). Eight items were longitudinally split in half whereby only one half has remained on-site. Shafts split this way include three spruce and five pine fragments. In three cases splitting occurred right along the pith, in two cases parallel to the pith, and in a single case the item was split tangentially and down to the pith. In a single instance, both split halves are present on-site (ID 8988). The artefact was first chopped in two as a diagonal bevel cut at one shaft end shows (Fig. S11). One part has been removed from the site while the remaining part (ID 8988) was then split in half along the pith.

Beyond the splitting of shaft fragments, evidence of more complex reworking has been noticed in three spruce artefacts. One shaft fragment (ID 7107_7108) with a complete diameter and covered in cambium bears a 20 cm long tangential splitting mark that was later scraped/abraded resulting in a smooth tangential surface (Fig. S12). This tangential surface is truncated by splinter negatives from a later shaft break. Small but slender splinters have been removed from the split surface another split wood (ID 11125). The length of these splinters was determined by deep perpendicular incisions prior to the splitting, which is comparable to a stop notch in Upper Palaeolithic burins (Fig. S13). Five slender splinters were thus produced from this wood core resulting in splinters of up to 8 cm length. All produced splinters have been removed from the site. Finally, the longest shaft fragment (ID 11383_12886) shows six splitting marks with lengths between 13 and 33 cm (Fig. S14). All splinters produced from this core have been removed from the site. Their extraction lead to the tapering of the shaft fragment over 54 cm becoming more acute over the last 16 cm. Although the resulting tool end is partially eroded and deformed, it bears use wear in the form of small splinter negatives and rounded edges likely caused during usage in abrasive tasks (see below). Shaft fragments have thus been frequently reworked by splitting while fewer served as cores for producing split woods. The resulting products were removed from the site, while the remaining artefacts evidence on-site recycling of shaft fragments.

Taphonomic traces are present on all shaft fragments. Surface weathering and sediment erosion have been observed on all items and bacterial decay likely lead to the softening of the wood. More invasive surface alterations occur in the form of surface damages in seven artefacts, cracks and flaked surfaces are frequent, whereas trampling marks occur in four. All items are compressed by superimposed sediments as evidenced either by oval cross-sections and/or longitudinal compression cracks. Transverse surface breaks are present on all items.

Finally, excavation damage has been observed among three items, twice in the form of transverse break surfaces, i.e. items fragmented during excavation and some localised compression and striations. In a single case delamination might partially be due to post-excavation drying out (ID 11979) while one item was sampled for a thin cut (ID 7296).

MNI of hunting weapons

Given the fragmented character of many spears and DPSs and the abundance of point and shaft fragments, the question arises, how many hunting weapons were indeed present at Schöningen 13 II-4? In order to reach at an approximate number, characteristic elements of each tool type (spear vs DPS) have to be considered in a logic order (Table S10). Firstly, complete spears and DPSs are considered whereupon inferences on fragments are made. Complete spears (n= 3) show a front point with the pith exiting along the tip's side, a long shaft and a back point with the pith in its centre. Contrastingly, complete DPSs (n= 2) taper into two equally shaped points and are less than 0.9 m long while ethnographic data entail DPSs of up to 1.03 m length (15). Spears and DPSs are commonly made on roundwods and therefore, we categorised roundwood fragments according to point manufacture and metric values. Roundwood fragments that taper toward one end and measure >1.1 m in length qualify as spears (n= 8), whereas shorter fragments that taper toward both ends qualify as DPSs (n= 4). Roundwoods shorter 1.1 m and tapering only toward one end are categorised as point fragments (n= 17), while roundwoods without taper are categorised as shaft fragments (n= 13). Both fragment types could have been parts of either spears or DPSs.

In the next step, point and shaft fragments are considered. Besides the clearly identified hunting weapons (3 complete and 5 fragmented spears, 2 complete and 4 fragmented DPSs), 10 front points, 4 back points, 3 front or back points, as well as, 13 shaft fragments probably were parts of former spears or DPSs too (Table S10). In order to reach an approximation of the actual number of hunting tools present at the site, the minimum number of individuals (MNI) has been calculated for spears and DPSs following two scenarios. (1) All point and shaft fragments are from spears (SpearMax). (2) All points and shaft fragments are from DPSs (DPSMax). Both scenarios are subject to limiting factors:

1. only roundwoods are included as there are no point or shaft fragments made on split woods
2. spears were made from spruce/larch and pine whereas DPSs are made from spruce only => spruce/larch fragments can be from spears or DPS, pine fragments are from spears only
3. all back points (pith in central position, growth direction) must come from spears
4. shaft fragments covered in cambium must come from spears as all DPSs are debarked
5. the combined length of shaft fragments must exceed the greatest length of complete spears (2.5 m) for it to create an MNI >1 in spears
6. MNIs of shaft fragments are combined with point fragments meaning that e.g. a front point + a back point + a shaft MNI= 1 result in a spear MNI= 1

Spruce/ larch: Artefacts made from spruce/larch comprise three complete spears, four fragmented spears with the back point missing, eight front points, three back points, and three front or back points resulting in an MNI of 15 spears, if all the fragments belonged to spears (SpearMax scenario; Table S11). In this scenario, DPSs made on roundwoods would retain an MNI of 4. If however all relevant fragments are calculated as DPSs (DPSMax scenario), spruce/larch spears and DPSs would both reach an MNI of 7 each (Tables S12-S13). Shaft fragments covered in cambium that should belong to spears have a total length of 1.5 m (Table S14). Assuming 2.5 m length for a complete spear, they would provide an MNI of 1. Debarked shaft fragments have a total length of 0.8 m and could be from either spears or DPSs, equally providing an MNI of 1 in either case. Combined with nine front and front/back point fragments, the MNIs of spears and DPSs do not change.

Pine: Artefacts made from pine comprise a single fragmented spear with a missing front point, two front and two back point fragments, resulting in an MNI of three pine spears (MNI= 3; Table S15). Pine shaft fragments have a total length of 1.4 m (Table S14). Assuming 2.5 m length for a complete spear shaft fragments would provide an MNI of 1. Combined with three remaining front and back point fragments, the MNI does not change.

Total: When considering all complete and fragmented artefacts found until 2008 this way, and depending on the scenario, this would result in 7–15 spears made from spruce/larch, three spears made from pine, 4–7 DPSs made from spruce/larch roundwoods, and two DPSs made on split woods (Table S16). Altogether, these 44 artefacts represent a minimum of between 19 (all possible fragments calculated as DPS) and 24 hunting weapons (all possible fragments calculated as spears). Adding the recently found DPS (14) we end up with 20-25 hunting weapons for Schöningen 13 II-4.

Domestic tools

Pointed split woods

The assemblage contains 24 pointed split woods with a single tapering end (Table S17). Theoretically, these pieces might be broken DPSs on split woods. However, they are considerably shorter than DPSs on split woods (max. length of 36 cm in pointed split woods vs min length of 45 cm in split DPSs), have a smaller median diameter (1.1 cm) than the two DPSs (1.8 cm), and taper more acutely (0.1 Tp compared to 0.06 Tp) (Tables S7, S17). Also, 14 items lack debarked surfaces consisting only of split ones. Therefore, we decided to separate them from the DPSs.

Eleven items belong to wood artefact category 1, seven to category 2 and six to category 3 (Table S17). Seventeen artefacts are made from spruce, five from spruce/larch and two from pine. Complete tips are preserved on 20 items while the others clearly taper. Lengths vary between 4.6 cm and 36.0 cm (median= 14.5 cm). Maximum widths range from 0.8 to 2.7 cm (median= 1.4 cm) while maximum thickness varies between 0.4 and 1.9 cm (median= 0.7 cm) meaning that particularly slender split woods were used to shape points on them. Tapering varies considerably with a change in width per centimetre length of 0.04–0.17 cm (median = 0.1 cm) and a thickness change of 0.00–

0.13 cm (median = 0.04 cm) over 4.5 to 28.5 cm length (median = 10.0 cm).

The *chaîne opératoire* differs from artefacts made on roundwoods. Three split woods were made from trunks while this is unclear in all others. Ten artefacts have debarked surfaces with tool marks that compare well to other artefact categories speaking for the recycling of roundwood artefacts. All other items solely consist of split surfaces (Table S17). Knots are present only on four items, so that wood portions without knots and natural flaws have been selected to produce split woods. Such flawless wood was most likely produced from the base of old trees where former branches had been shed off already and were overgrown (26). Such wood portions are particularly easy to split in a controlled manner.

Besides longitudinal splitting, the tools were worked via localised splitting, scraping and abrasion, forming splitting marks and smooth surfaces. Smoothing covers complete surfaces in 16 items while only the first 3-4 cm of the tip have been smoothed in the remaining eight ones. In five items surface facets aided in shaping the point next to splitting and smoothing (Figure 2a). The production sequence is thus fairly standardised including splitting and smoothing to shape small points on flawless split woods.

Use-wear might be present in the form of use-polish on preserved tips but this may also result from abrasion after the shaping phase. In addition, longitudinal crushing and micro-splintering have been observed in six tips providing clear evidence of use. Two items bear dark discolouration, which might result from charring or residue.

Taphonomic traces are abundantly present on all pointed split woods (Table S17). Surface weathering and sediment erosion have been observed on all items and bacterial decay likely lead to the softening of the wood. Although it cannot be ruled out that the shaft ends have been cut off deliberately, the transverse surfaces are too eroded to be certain. Invasive surface alterations occur in the form of surface damages in nine artefacts, while cracks and flaked surfaces are rare as are trampling marks. All items are compressed by superimposed sediments evidenced either by longitudinal compression cracks. Rot, fungal and insect infestation have been observed in two items. Finally, excavation damage has been observed among seven items, twice in the form of transverse break surfaces, i.e. items fragmented during excavation and some localised compression and striations in two cases (Table S17). A single item was sampled to study bacterial decay (ID 9424).

Split woods with round tool ends

The assemblage contains 11 split woods with round tool ends, eight belong to wood artefact category 1 and three to category 2 (Table S18). Nine artefacts are made from spruce, one from spruce/larch and pine respectively. Complete tool ends are preserved on eight items. Lengths vary between 14.5 cm and 82.8 cm (median= 27.0 cm). The maximum width ranges from 1.7 cm to 3.8 cm (median= 2.6 cm) while maximum thickness ranges from 0.7 to 2.5 cm (median= 1.1 cm). In comparison to pointed split woods, sturdier blanks were selected (median lengths is 27.0: 14.5 cm, widths is 2.6: 1.4 cm, thickness is 1.1: 0.7 cm).

The *chaîne opératoire* compares well to that of the pointed split woods including similar working traces (Table S18). Five split woods have been identified as coming from trunks and another one potentially so. Eight artefacts have debarked surfaces with tool marks comparable to those of other artefacts made from roundwoods speaking for the recycling of former tools. Knots and/or knot holes are present on the majority of them. Natural flaws are absent speaking for a thorough selection of wood properties comparable to those of pointed split woods.

Besides splitting, the tools were shaped via localised splitting followed by scraping and abrasion. Smoothing usually covers entire surfaces and the tool end. Six items have been worked more intently including the shaping by surface facets, scraping or long splitting marks (Table S18). The production sequence is thus fairly standardised including splitting to form a round tool end on flawless split woods followed by smoothing.

A single tool has been reworked multiple times (Figure 3; ID 7288_7289_7304_7305). The length was reduced by inserting diagonal cuts followed by snapping off the resulting fragment, abrading the tool, and using the tool end. At least two rounded tool ends are preserved on that item.

Use-wear might be present in the form of use-polish on the preserved tool ends but this may also result from abrasion. Contrastingly, micro-splintering has been detected on eight rounded tool ends and combined with longitudinal crushing in two cases, this provides clear evidence of use (Table S18; Figure 3). Four items bear orange flecks on their surfaces and while this is connected to iron-mineralisation in general, in one case this feature concentrates at the tool end and might be residue from tool-use (Figure 3).

Taphonomic traces are present on all rounded split woods (Table S18). Surface weathering and sediment erosion have been observed on all items and bacterial decay likely lead to the softening of the wood. Although it cannot be ruled out that the shaft ends have been cut off deliberately, the transverse surfaces are too eroded to be certain. Invasive surface alterations occur in the form of surface damages in six artefacts, while cracks and flaked surfaces are equally common, but trampling marks are rare. All items are compressed by superimposed sediments evidenced by longitudinal compression cracks.

Finally, excavation damage has been observed among three items in the form of transverse break surfaces, i.e. items fragmented during excavation and some localised delamination due to drying out. A single item was sampled to investigate its annual ring sequence (ID 11383_12886).

Working debris

The assemblage contains 109 split woods/splinters and fragments considered as working debris. Nine artefacts belong to wood artefact category 1, six to category 2, and 94 to category 3 (Table S19). The majority of them belongs to the split woods or splinters (n= 103), followed by branch fragments (n= 4) and flaked off surfaces (n= 2). The objects are made from spruce in 70 cases, spruce or larch in 10 cases and from pine in 29 instances. Lengths vary between 2.1 cm and 31.0 cm with a median value of 7.7 cm. The maximum width ranges from 0.5 cm to 2.5 cm (median= 1.4

cm) while maximum thickness ranges from 0.1 to 2.2 cm (median= 0.5 cm). The split woods/splinters are of particular interest as they probably represent working debris from the shaping and reworking of wooden tools and their metric values are closest to those of the pointed split woods.

The *chaîne opératoire* of the working debris is comparable to that of other artefact categories, however, clear working traces are less frequent (Table S19). Knots and/or knot holes are present on 22 items including the four branch fragments. One branch fragment has callus tissue preserved. Knots were either torn off or abraded in most cases while two branch fragments also have cut knots. Debarked surfaces are preserved on 52 items whereof 19 show clear working traces in the form of annual ring surfaces, scraping marks, tool facets, or surface facets (Figs. S2-S3). Contrastingly, 57 items consist only of split surfaces. Natural flaws are absent from the debris speaking for a thorough selection of blanks (trunks, branches, former artefacts) in the process of splitting.

Besides splitting, 13 artefacts were re-worked via splitting and further smoothing leaving behind splitting marks and smoothed surfaces (Table S19). One branch was diagonally cut in two whereof one fragment (ID 12113) was further split in half leaving behind a branch fragment and a split wood on-site while the other parts were removed. Smoothing on split woods covers either entire surfaces or just parts of them. Other working traces such as scraping marks, surface facets and bevel cuts could be identified in three split woods. Eleven split woods taper and those might have been pointed split woods once too (IDs 1904, 4474, 6849, 6850, 7909, 8369, 9200, 9724, 11584, 11714, and 15151). The points, if ever present, must have broken off in antiquity making an assessment difficult. The tapering items belong to category 3 and are made from spruce/larch like most of the pointed split woods. Also the median width (1.3 cm) and median thickness (0.5 cm) compare well to pointed split woods (Tables S17, S19).

Use-wear on debris is present in the form of potential use-polish and transverse break surfaces. Eleven items including four tapering ones seem to have use polish on them while the same number of items displays transverse break surfaces. Finally, dark discoloration was observed on three split woods and its origin remains unclear but might be connected to fire exposure and heat treatment.

Taphonomic traces are present on all items in the form of surface weathering and surface erosion, while bacterial decay likely lead to the softening of the wood (Table S19). Invasive surface alterations on the other hand are comparatively sparse and occur in the form of surface damages in 11 artefacts, cracks in eight items and flaked surfaces in 11 objects. A single split wood has a trampling mark on it. All items are compressed by superimposed sediments evidenced by longitudinal compression cracks.

Excavation damage has been observed among 39 artefacts, mostly in the form of transverse break surfaces, i.e. items fragmented during excavation (n= 34), and some localised compression and striations in five objects. Two artefacts show delamination and drying cracks that might be due to them drying out after the excavation.

Curated vs expedient technologies

Building on the works of Lewis R. Binford, Bamforth (48) states, "Technologies based on curation comprise tools that are effective for a variety of tasks, are manufactured in anticipation of use, maintained throughout a number of uses, transported from locality to locality for these uses, and recycled to other tasks when no longer useful for their primary purposes".

In this sense, spears and DPSs at Schöningen 13 II-4 fall into the definition of curated tools. As the raw materials (spruce, spruce/larch, and pine) used to produce them were not available at the site (49), hunting weapons were transported to the lakeshore probably in anticipation of hunting taking place here and likely as finished tools. While it is clear that tool maintenance did not take place at Schöningen 13 II-4 exclusively, some artefacts likely represent on-site tool curation. A point fragment, Spear V and Spear X have crudely re-worked front points substantially altering their original outline (Figs. S3g, S4; see above). The same is true for two DPSs' points (IDs 15677, 7344_73612; Figure 1; Fig. S7; see above). Such treatment stands in contrast to the commonly finely worked points and tool surfaces that characterise Schöningen's wooden hunting weapons. The aforementioned tools thus must have been curated/maintained, i.e. their point was re-sharpened. Given the character of Schöningen's lakeshore as a hunting place, not much time was probably spend on smoothing these points as they were likely to be used immediately after the repair stage. These tools very likely had been repaired on-site before re-use and discard. Tool maintenance activities further are reflected in the abundance of working debris such as wood chips on-site that in part can be understood as waste products of the tool repair procedures (50).

Among the split wood tools, tools that were produced from wood selected from particularly old trees and then were purposefully transported to the site, fall within the definition of curated tools (n= 23; see above). A round-ended split wood (ID 7288_7289_7304_7305; Figure 3) provides direct evidence of on-site tool curation. It has been shortened multiple times as refitted fragments evidence and at least two of them have a tool end preserved that show signs of use.

With respect to expedient technologies, Bamforth (48) states, "Technologies based on expediency comprise tools that are manufactured, used, and discarded according to the needs of the moment".

In this sense, on-site production of expedient tools at Schöningen 13 II-4 is closely connected to the recycling of former hunting weapons and the manufacturing of split wood tools. Twelve split wood tools with abundant knots and working traces mirroring those seen in hunting weapons indicate the recycling of former spears and/or DPSs (Tables S17-S18). These are mostly split woods with a rounded tool end (n= 8), but also a few pointed specimens (n= 4) are present. Split wood tools were probably used in processing hides and vegetal material on-site. Split wood tools that were split along the pith and thus preserve the complete width of the former tool, bear widths comparable to shafts (2.1–3.8 cm; Tables S6-S7, S9, S17-S18). Shafts were most likely used in tool recycling as they provide more volume to work with than points do. Incomplete spears such as Spear IV and Spear X missing substantial parts of the shaft then speak for such on-site recycling of these elements (Figure 1; Table S6). This is further reflected by the incomplete preservation of shaft fragments, whereby the

length is substantially reduced in comparison to complete spears and DPSs (Tables S6-S7, S9). Further recycling of shaft fragments was done by longitudinal splitting and in all but one case, only one half was recovered on-site (Figs. S11, S13; see above). Two further shaft fragments were recycled showing the recurrent removal of split woods, which likely occurred on-site too (IDs 11125 and 11383_12886; Fig. S14; see above). Finally, the small amount of only 12 recycled split wood tools in comparison to the abundance of woodworking debris including 103 split woods (ratio= 0.1:1), speaks for wood recycling and production of expedient tools on-site.

Morphometric analysis

Keeping in mind the small sample size, we outline here some results of simple univariate statistics. The descriptive statistics of the sample of spears (Table S20) suggests that for the complete and nearly-complete examples, the length is the least variable measure (CV = 12%). This suggests that length is a design feature that is relatively tightly constrained. The rate of taper of the front points of spears also have low variability (CV = 16%), in comparison to the back points (CV = 26%), showing that the front points are more standardised than back points. Front tapers are also on average twice as long as back tapers. The annual ring count is the most variable (CV = 34%), suggesting that while the hominins selected spruce and pine, there was variability in the number of annual rings, and hence density. However, the median value of 51 rings suggests a tendency towards a higher number of annual rings, with the lowest value of 18 annual rings relating to the only spear made from pine (Spear IV).

In a recent review of the use of morphometrics of ethnographic spears to determine function as thrust and thrown weapons, the only measurement that was found to reliably correlate with mode of delivery was the location of the maximum diameter in relation to its overall length (LMD %) (51). In that study there was a moderate correlation to the point of balance (POB %). In comparison to ethnographic spears, the range here for the Schöningen spears' LMD % (ca. 18.9 % to 40.8 %) fits within the range of known ethnographic throwing spears (n=14; 4% to 61%). In the case of the Schöningen spear sample, all of the LMD % values suggest a balance point at or in front of the midpoint (Table S20). Therefore, even if the largest Spear VI is not suitable for flight due to a natural kink, its morphometrics alone do not rule out functionality as either a thrown or thrust weapon (51). However, fully accurate replicas of each spear would need to be made to test this further. The maximum diameters of the Schöningen sample (2.3 cm – 4.5 cm; mean = 3.3 cm) is larger than ethnographic spears of all delivery methods (1.3 cm – 3.2 cm; mean = 2.1; n = 58) while length values are at the shorter end (Fig. S16). Although maximum diameter does not correlate with mode of delivery (51), it is interesting to note that these early spears are more robust than later examples associated to our own species, which could relate to physiological differences between contemporary *H. sapiens* and Middle Pleistocene hominins (e.g. 52, 53).

The double-pointed weapons designated as DPS were assigned on the basis of their shorter length and their two equally constructed points. In comparison to the spear sample, the maximum diameter

has a similar minimum value, but DPSs are generally thinner than spears (median = 2.4 vs 3.3) (Tables S20-S21). In contrast with rates of tapers of the spears, the tapers of DPS suggest a more complex picture. This may be because designating a back vs. front point of throwing sticks is not relevant, if they are not designed as penetrating weapons. Yet, the tapers of 'Point 2' are one of the least variable measures (CV = 18%). The meaning of this will require further investigation. The least variable measure is the LMD % (CV = 16%), further suggesting that this is an important feature for flight.

For the relevant variables, the pointed split wood tools are overall more variable than those for spears and DPS (Tables S20-S22). This likely, at least partly, relates to the high variability of their length, which likely signifies different tool functions, and/or the presence of some woodworking debris. The tapering rate is especially highly variable (CV = 84%), and with a higher maximum taper than spears and DPS.

A scatterplot compares the lengths and maximum diameters of DPS, Schöningen spears, and ethnographic wooden spears. The plot demonstrates a clear differentiation between the complete and nearly complete examples of Schöningen DPS and spears. In comparison with ethnographic spears, the Schöningen spears are overall more robust in diameter, but fit well within the sample in terms of length (Fig. S16).

The morphometric data was calculated in violin plots facilitating a means of cross-artefact comparisons (Fig. S17). In terms of taper length, points in spears and DPS show considerable overlap (Fig. S17a). Point fragments' taper lengths of front points (Point 1) are a better match to DPS, although back points (Point 2) overlap with both spears and DPS, so that these fragments could fit into either spears or DPS depending on the individual artefact. Pointed split woods minimally overlap with spears and are quite different from DPS points too, so that this distinct category looks relevant. Pointed split woods differ both in terms of the tool size, blank selection (split wood), and the taper length. Taper rates have significant overlap, similar means across all types, and are not particularly useful in differentiating between artefact types (Fig. S17b). The quarrel for the belonging of point fragments to either spears or DPSs thus remains inconclusive, while pointed split woods are quite distinct from spears and DPSs.

<u>Spatial distribution patterns</u>

Material classes

The site of Schöningen 13 II-4 can be subdivided into three broad zones, the lake zone in the east, the lakeshore in the west and a human activities zone sandwiched between them (Fig. S18). The general distribution including all finds shows they are mostly located in the human activities zone, while becoming fewer and more dispersed in the lake zone, whereas at the lakeshore they are comparatively sparse. Two finds concentrations are present in the central to northern parts of the human activities zone with the major finds concentration located north of Spears I to III and a second, more dispersed one, located south of the northernmost DPS. Besides that, smaller finds

concentrations are noticed throughout the excavation area but mostly in the human activities zone. Faunal remains and lithics show distribution patterns that are similar to one another and well agree with the general distribution pattern of finds (Figs. S19-S20). Natural and worked wooden remains on the other hand display a different distribution pattern (Fig. S21). The majority is found in the human activities zone while a considerable number is also located in the lake zone, whereas those at the lakeshore represent the smallest group. Wood is relatively light (lower density) compared to bone and flint and has the ability to float above water until water saturation is reached. Wood remains discovered in the lake zone might have drifted away from the lakeshore while others might have entirely escaped the archaeological record by floating away even farther. Wooden remains form no spatial clusters in the human activities zone where they are quite dispersed and only loose agglomerations of local character can be observed. Moreover, the southern portion of the human activities zone is almost bare of any wood remains. Contrastingly, woods form three small clusters in a northern part of the lake zone and these contain only natural wood fragments.

Wooden artefacts

Unlike, the general distribution of wood remains, the distribution of wooden artefacts forms clusters inside the human activities zone and in accordance with the density patterns, the human activities zone is subdivided into the four sectors A to D (Figs. S22-S23).

Sector A is bare of any spears but contains two DPSs and four front point fragments. A single point fragment (ID 3858) is made from pine while all other tools are made from spruce. The front points and DPSs might be regarded as parts of hunting equipment lost and/or broken during the hunting of prey. Tools on split woods are sparse and located along the southern edge of this sector. All of them were worked from spruce. The working debris found in this sector is made from spruce and pine, but seems unrelated to the hunting activities, although most of it was found just west of a reworked DPS ('skewer').

Sector B constitutes the major accumulation area of wooden artefacts and encompasses half of all spears. Besides two complete spears (I, III), there also are three fragmented ones (II, IV, X) and two DPS fragments (ID 7344_7363, 7724_7726_7727). Point and shaft fragments are comparatively abundant in zone B as are pointed split woods, whereas there are only two round-ended split woods. Tools made from spruce or pine are both frequent with 15 and 11 items respectively while there are seven spruce/larch objects too. Working debris on the other hand overwhelmingly comes from spruce while pine and spruce/larch items remain exceptions thus standing in stark contrast to the aforementioned tool groups. Furthermore, their distribution pattern differs from that of tools. While tools are found clustering in an area around Spears IV and X, debris accumulates in two areas west and south of these tools. It thus seems that the depositions of spears, other tools, and debris are largely unrelated. The amassment of finds in sector B reflects different activities carried out on-site and possibly in sector B itself including woodworking. However, it also seems possible that this sector served as a dumping zone for wood rubbish. The (near-)complete intact Spears I and II do not adhere

to this pattern and might be equipment lost during hunting activities.
Sector C is essentially similar to sector B in respect to wooden artefacts, although find numbers are lower and the area is smaller. Sector C is characterized by pointed tools and debris, it contains one complete spear (V) and two fragmented ones (VI and VII). There is a single DPS (ID 10167), elegantly worked from a split wood, and some point fragments, while shaft fragments are near absent (ID 9921). Pointed split woods are again frequent, while there is only one rounded split wood (ID 9349). A single pointed split wood (ID 9920_9922) and the shaft fragment (ID 9921) found next to it were made from pine while all other tools are made from spruce or spruce/larch. Contrastingly, debris from spruce and pine occur in almost even numbers in sector C, plus few spruce/larch items. The distribution of both categories, tools and debris differ too. While tools accumulate in the eastern part of section C, debris is found more frequently around Spear V. The tool composition of sector C is thus comparable to that of sector B while it contrasts in terms of wood species representation meaning that tools made from pine are abundant in sector B while being sparse in sector C, whereas this aspect is reversed in respect to working debris.
Sector D contains only three artefacts that are located about 10 m south of sector C. These are two shaft fragments made from spruce and spruce/larch respectively that are partially split. The most intriguing item in sector D is a broken shaft fragment that was re-used to produce split woods from it and to shape a round tool end on it (ID 11383_12886; Fig. S14). These items speak for the recycling and re-use of shaft fragments as the main task in the remote sector D.
Two further observation are worth noting. Firstly, at the lakeshore west of sector D, a point fragment with a longitudinally split and abraded tip (ID 14852), and a pointed split wood whose tip has similar dimensions has been noticed (ID 14903) and this might be a particular working area of unknown function. Secondly, in the south-eastern part of the lake zone, three split woods with a rounded tool end (IDs 12071, 12072, and 12073) have been noted next to a point fragment (ID 12074). The reason for this accumulation outside the human activities zone is unclear, yet natural causes seem unlikely.
The spatial pattern of the various artefact classes at Schöningen 13 II-4 thus show dynamic distributions varying with wood species, artefact category, and tool type (Fig. S23). Wooden artefacts are mainly found in two central sectors (B and C) reflecting similar tasks carried out on-site (hunting, reworking of tools, dumping of wood debris). Peripheral sectors (A and D) display more specialised activities (hunting and reworking; Fig. S24). Such distribution pattern seems thus best explained by repeated site occupations over a longer period encompassing similar activities performed repeatedly.

Discussion

Simple stone tools vs sophisticated organic technologies

Human occupations in Central Europe commence later than in other parts of Europe, i.e. during the Middle Pleistocene around 600 ka (MIS 15) when the European Lower Palaeolithic can be subdivided into two geographic regions. The Lower Palaeolithic in north-western, western and

southern Europe is characterized by assemblages with handaxes next to those solely relying on flake production, whereas central and eastern European assemblages generally lack handaxes but produce numerous small tools on flakes (36, 37, 54, 55). Few bifacial tools are however present at later Central European sites like at Bilzingsleben (n> 9) and Vértesszőlős (n= 3) that date to MIS 11 or 9 (36, 56).

Pre-Holsteinian (MIS 12) assemblages like Korolevo VI and Kärlich-Seeufer mostly produced backed flakes with lightly retouched edges. Contrastingly, at Holsteinian (MIS 11) and later sites like Vértesszőlős, Bilzingsleben, and Schöningen thinner flakes were either produced from cores or natural chunks were directly retouched into tools often bearing heavily retouched edges (22, 37, 55, 57). Lithic industries in Central Europe can thus be described as simple yet efficient, making use of natural chunks and small nodules speaking for a minimal investment in lithic production. This stands in contrast to another novelty characterizing Middle Pleistocene assemblages, i.e. hafting of lithic implements.

Producing multicomponent tools, such as hafted tools requires knowledge of the materials employed, technological know-how, crafting skills, as well as, planning abilities (58, 59). The *chaîne opératoire* of hafted tools might be rather complex and multiple interconnected steps need to be followed to achieve that goal (60). Beginning with an arising or expected need, a mental tool concept must be envisioned or drawn from the culturally transmitted tool-repertoire (58–63). Thereupon, raw materials need to be acquired (selection, procurement, and transport) and components shaped, which in itself requires multiple steps (e.g. stone tool vs wooden handle). At least two tool components, often made of different materials, are then combined according to the intended tool function. As string or adhesives are employed as supporting elements, their production also requires a tool concept and further production steps must be followed (59, 60, 64, 65). Adhesive production is a chemical procedure, e.g. when birch bark under usage of fire is turned into birch tar, so that knowledge of that procedure is a prerequisite as much as is know-how of the various steps and conditions involved. Naturally, not every time those tools and procedures need to be invented anew and cultural transmission would be key to warrant the transfer of knowledge over generations, across space and social groups creating tool making traditions (61–63).

Hafted tools in Africa might be as old as 500 ka as use-wear on lithic spear implements from Kathu Pan in Southern Africa and on later core-axes from Site 8-B-11 in Sudan suggest (66, 67). In Europe, Schöningen provides evidence of wooden socketed handles (so-called clamp shafts) made from silver fir (n= 33) and coming from sites 12 II, 12 B, and 13 DB (22). Schöningen also provides evidence of hafted tool technology in the form of lithics with hafting use-wear and resin traces coming from Schöningen 12 II (68). Adhesives are more widely known from Middle Palaeolithic contexts beginning with MIS 8 in Europe (e.g. 64, 69, 70) and later evidence coming from Africa (e.g. 71) and the Levant (e.g.72, 73).

Bone technology is another aspect of organic technologies, although during the early and Middle Pleistocene, bone tools were mostly used as knapping tools to retouch lithics. The oldest such

evidence might come from Beds I and II at Olduvai Gorge in Tanzania where 125 bones with knapping marks were found (74). The site is between 2 and 1 Ma old. Another 86 bone tools from Swartkrans in South Africa showing evidence of tip shaping and were possibly used for extracting termites (75). The site is about 1 Ma old too. From beds III-IV at Olduvai Gorge come at least six bone tools and one of them might be a preform of a barbed harpoon dating to 700 ka (76). Three pointy/rounded bone tools from Broken Hill in Zambia probably date to 300 ka and were intentionally shaped (77).

European evidence of Middle Pleistocene bone technology is largely restricted to bone handaxes made form elephant bones and dating from 500 to 260 ka (56). At Gran Dolina TD10-1 and Bilzingsleben, two large bone sidescrapers were found respectively and both sites date to MIS 9 or MIS 11 (78, 79). Bilzingsleben also produced four flat bones with parallel and/or radial engravings on them (79). Beyond such exceptions, antler and bone was mostly used in retouching lithics from at least 500 ka onwards (80–85). 88 bone tools used as retouchers, anvils, and hammers for breaking up bones have been reported from Schöningen 13 II-4 (84). Another nine bone tools come from Schöningen 12 II (83). Three of them show polished and chipped tool ends and likely were used in butchering activities while four further and more elongated ones show rounded and polished tool ends and that have been used as hide smoothers. A single and elongated ivory object equally shows polishing at the distal end. Two other bone tools have been used in percussive tasks. Combined with the wooden artefacts, organic technologies from Schöningen thus illustrate a diversity of tasks that included hunting and butchering, but also domestic activities like lithic tool retouching and hide smoothing. Finally, the wooden handles from Schöningen suggest that some of the lithic tools might have been part of composite tools.

Woodworking techniques during the Early and Middle Pleistocene

While woodworking technology is essential for our understanding of past human cognition and behaviour during the Pleistocene, currently information on woodworking techniques are sparse in the literature and often do not go beyond general accounts if at all. However, some data could be extracted from a few publications (4, 18–20, 86).

Five wooden artefacts have been reported from multiple layers at Kalambo Falls in Zambia dating from 480 to 320 ka BP (4). Working traces have been studied and compared to experimental data. Functional interpretations rest upon ethnographic comparisons and regional crafting traditions. A 143 cm long and 25 cm wide wood log was shaped from the trunk of the large-fruited bushwillow (*Combretum zeyheri*). The item is mostly debarked, both ends taper, while a central notch (13 x 11 cm) was created by chopping. It remains unclear which technique was used to create the taper at both ends, but scraping marks are present on the surfaces and along the notch's shoulders speaking for intentional edge and surface smoothing. The notch was connected to a larger log underlying it, equally shows signs of working and equally tapers. Both logs are interpreted as part of a wooden structure of unknown function. The artefact evidences the application of chopping as a manufacturing

technique to shape surfaces.

Further tools include a digging stick, a wedge possibly used to split wood, a board of unknown function, and another pointy tool of unknown function. They are made from bushwillow (*Combretum zeyheri*) and the sausage tree (*Kigelia africana*). Tree trunks and branches were used as raw materials. The small pointy tool might have been manufactured on a split wood. Two objects are completely debarked while two others partially retain outer bark. Striations and facets speak for the tool surfaces being scraped and possibly carved/planed. The tool end of the wedge is shaped by a bevelled cut, the tool end of the digging stick possibly by carving/planing. The handles of the digging stick and the small pointy tool are formed by side branches. Both ends of the wooden board bear chopping marks meaning the artefact was trimmed to its intended length.

The 35 wooden implements from multiple layers of the 360-250 ka old site of Gantangqing in China include digging sticks and complete, small, hand-held pointed tools (18). 23 items retain a tapering bevelled or rounded end while five further pieces have a hook-like tool end. The latter appear to have been shaped from the base of a trunk and the top of a root. The largest item measures 87.5 cm in length with a 8.5 cm diameter. The dominant wood species identified is pine (*Pinus sp.*), while few items had been worked on deciduous wood. Most items are made on branches while nine might have been made on split woods or natural splinters. Heartwood was used in four cases. Many of the items had starch grains preserved on them speaking for a usage as digging tools. All objects are debarked and the branches carefully removed, some by cutting. Shaping was done in alternating directions and bevelled cuts applied to form the chisel-like tool ends. Stop marks indicate the working from the point toward the shaft in some instances and the tool surfaces probably were smoothed. While the authors make theoretical accounts on how the wooden artefacts might have been manufactured (18), these have not been systematically checked against the manufacturing traces, e.g. usage of an axe or adze has been suggested for the initial shaping while corresponding shaping facets do not seem present on the artefacts (40).

The 58 digging sticks from the c. 170 ka old site of Poggetti Vecchi in Italy were manufactured from straight boxwood (*Buxus sempervirens*) branches (19) Boxwood is a slow-growing evergreen that is particularly dense, hard, and thus does not break easily. Complete items were about 1m long while diameters range from 2.5 to 4 cm. The branch was chopped off the tree and the twigs carefully removed and abraded. Before scraping off the bark, the artefact was burnt to allow for easier removal of the bark. The natural taper of the branches was used to fashion a blunt point at the thinner end. Sometimes the end was sharpened by an oblique bevelled cut. The thicker end was used to shape a blunt, rounded handle on them. Shaping of both ends was assisted by burning of the wood surface to permit easier working of this hard wood.

A 2.4 m long wooden spear made from yew wood allegedly was discovered between the ribs of a straight-tusked elephant at Lehringen in Germany (86). According to palynological results, the spear can be dated to the last interglacial, c. 130-110 ka BP. Macroscopic traces on the spear have been compared with experimental data. The spear was made from a yew trunk that was likely chopped

down. The spear is completely debarked, branches were cut off and the surfaces were smoothed by scraping. The front point is oriented toward the base of the tree comparable to the Schöningen spears. The front point was shaped by carving/planing as surface facets show. The point is offset with the pith emerging on its side. No information on the back point is provided other than it being truncated by long splinter scars similar to the Schöningen spears. The documented traces are comparable to those observed on wooden hunting weapons from Schöningen.

A single digging stick and another wooden object have been recovered at the site of Aranbaltza III in Spain (20). The digging stick wad made from yew (*Taxus baccata*) and the associated layer is radiometrically dated to 130-70 ka. The 15.2 cm long branch fragment has a diameter of 2.9 cm and is tangentially split. The item is completely debarked and side branches were torn off and abraded. The surface is smooth with dark discoloration that might be linked to heat treatment. One end tapers into a round tool end. Micro-splintering at this otherwise round tool end speak for usage in an abrasive task such as digging. The shaft might have been intentionally split in half as a bevelled cut shows.

The gathered data shows a broad spectrum of wood species employed in producing wooden artefacts during the Pleistocene. Raw material selection was likely guided by regional resource availability and the material properties' performance in the intended task. Chopping as a woodworking technique must be assumed wherever trees were felled or larger branches were cut from a tree, although such traces are usually masked by later manufacturing steps. Wooden tools consistently have been debarked with the exception of a few artefacts from Schöningen and Kalambo Falls. Branches were carefully removed, mostly by cutting and/or tearing followed by smoothing. Surfaces have been worked by scraping and carving/planing. Chopping as a manufacturing technique is evidenced only at Kalambo Falls where a large log and a board are worked in that technique, whereas tools with smaller diameters were worked in other techniques. This compares well to findings from other sites where tools usually have diameters <5 cm and thus are not sufficient for chopping in a controlled manner. Not much data is available on the shaping of tool ends, but tool ends in digging sticks routinely seem to have been manufactured by bevelled cuts, but also by carving/planing. Spear and DPS points were shaped by the removal of small splinters from point to shaft and by carving/planing. Beyond Schöningen, the splitting technique might be present at the contemporaneous sites Gantangqing and Kalambo Falls BLB 4, although we cannot exclude natural splinters had been used in the latter cases. Intentional burning of wood surfaces is evidenced only at Poggetti Vecchi thus far and connected to the working of the particularly hard boxwood. Spear and DPS points at Schöningen might have been dried by heat over an open fire as the observed dark stains might suggest. However, AT-FTIR analyses remained inconclusive as were IR analyses conducted at Kalambo Falls. Possible heat retreatment was suggested for Aranbaltza III. The spectrum of known woodworking techniques during the Early and Middle Pleistocene thus comprise chopping, carving/planing, cutting, tearing (branches), scraping, abrading, splitting, and use of fire, while drilling has not been observed yet.

Measuring technological complexity

In a recent paper on technological complexity of modern societies, Broekel (87) states, “Technological systems have become increasingly complex over time because of knowledge and technologies’ cumulative nature, with each generation building upon the technological environment established by its predecessors…”. This is in agreement with observations that technological complexity had begun increasing already early in human evolution (61, 62, 88–91).

Measuring technological complexity has been performed in a number of studies applying different methods including techno-units (91), cognigrams (62), petri networks (92), or procedural units (88). Based on descriptions of relevant Early and Middle Pleistocene technologies in the literature, we calculated procedural units as a means of comparison through time including technologies made from different materials. Procedural units are, “mutually exclusive manufacturing steps that make a distinct contribution to the finished form of a technology” (88). The concept follows the logic of the *chaîne opératoire* thus being in line with our approach concerning woodworking technology at Schöningen. Here, we expand the concept of procedural units by including raw material acquisition and transport before the tool manufacturing stage as well as tool use and tool discard after the manufacturing stage.

The graph comparing brain size evolution and technological complexity involves organic and inorganic technologies as well as composite tools arranged in two time slots, i.e. Lower Pleistocene and Middle Pleistocene (Figure 6, Table S23). Technologies are ordered by the number of procedural units (primary order) and their first appearance in the archaeological record (secondary order). Bone retouchers, pointed bone tools, and hide smoothers comprise the spectrum of bone technologies. Simple lithic tools, flaked lithic tools and shaped handaxes comprise the spectrum of inorganic/mineralic technologies. Spears, DPSs, and split wood tools (pointed and round-ended ones) represent woodworking technologies. Finally, composite technologies consisting of organic and inorganic components, include hafted tools with a string and with adhesive. For the sake of completeness, the materials and tools likely used in the tool’s production sequence have been documented too (Figure 6, Table S23).

Bone retouchers, pointed awls, and fur smoothers are reportedly produced on bone fragments that are by-products of the bone marrow extraction process for which fresh bones were intentionally fractured (83, 93, 94). Fragments of long bones were selected likely for their sturdiness and thick walls. Occasionally, relatively intact bones resulting from butchered carcasses had been used instead. In the Early and Middle Pleistocene, such bone tools are typical ad-hoc tools making use of materials that are readily available at the site. Tool shaping often was minimal, focussing on regulating edges and shaping the tool end. Due to the hardness of bone, stones must have been used in fracturing and shaping bone tools (83, 95) while sturdy bones like horse and bison metapodials likely were used in bone fracturing too (84).

Simple lithic tools in the Central European Middle Pleistocene were manufactured directly on natural

chunks like frost sherds (37). However, this does not imply the raw material was readily available at the archaeological site and likely had to be transported to it. Tool retouching was performed with small hammer stones. Flaked lithics were extracted from little modified/prepared cores with hammer stones (37), but the raw material had to be brought to the site as well. Handaxes are more complex tools that involve more manufacturing steps (62), with the raw material rarely available at the site of use. In the graph, we considered a relatively simple handaxe with four manufacturing steps. The roughing out from an amorphous block was performed using a hammer stone and so was the overall shaping process while the thinning stage was performed using an antler or bone hammer. Finally, the tool retouch was achieved using a small hammer stone. Handaxes were transported over longer distances and used over extended time spans (54, 55, 62).

The various woodworking technologies have been described in the main text and above.

Concerning hafted tools, we have considered a relatively simple technology like a hafted knife or scraper that consist of only three components – a wooden handle, a lithic tool end, and binding material, i.e. string in one case and adhesive material in the other. For all components, we assume they were manufactured in the simplest way possible, including the condensation method used in producing birch tar adhesive (65). Raw materials used to manufacture a hafted tool include a wooden handle e.g. fir (96), a string made from plant fibres, e.g. inner bark (97) or sinew, and a lithic implement e.g. flint. In the adhesive scenario, an open fireplace was considered as part of the manufacturing process together with a single stone used as condensation core (65). We assume all raw materials were not available at the site and had to be transported. We further assume individual components were purpose-made and not by-products of former activities so they were purposefully selected and gathered/harvested. As hafted tools have the advantage of making re-tooling easier, meaning the lithic component was discarded once exhausted and was then replaced with a new lithic element while other tool components would continue to be used, retooling was considered as the final step rather than discard of the complete tool.

Figure 6 shows technological complexity broadly increasing with time. Woodworking technologies tend to be more complex (more procedural units) than bone and lithic lithic technologies, and are surpassed only in complexity by composite tools. Woodworking technologies as present at Schöningen, which is chronologically placed at the boundary of the Lower Palaeolithic and Middle Palaeolithic, exemplify technological complexity that is unknown from earlier sites, but foreshadow standardised production sequences (e.g. Levallois concept in lithics) that are typical of the following Middle Palaeolithic. Assuming woodworking technologies have been present for as long as lithic and bone technologies have, preservation of wooden tools clearly affects our understanding of technological complexity at any given time, meaning where wooden artefacts are not preserved, we might underestimate the cognitive abilities of prehistoric societies.

SI References

1. M. Dominguez-Rodrigo, J. Serrallonga, J. Juan-Tresserras, L. Alcala, L. Luque, Woodworking activities by early humans: A plant residue analysis on Acheulian stone tools from Peninj (Tanzania). *J Hum Evol* **40**(4), 289–299 (2001).

2. C. Lemorini *et al.*, Old stones' song: Use-wear experiments and analysis of the Oldowan quartz and quartzite assemblage from Kanjera South (Kenya). *J Hum Evol* **72**, 10–25 (2014).

3. S. Belitzky, N. Goren-Inbar, E. Werker, A Middle Pleistocene wooden plank with man-made polish. *J Hum Evol* **20**(4), 349–353 (1991).

4. L. Barham *et al.*, Evidence for the earliest structural use of wood at least 476,000 years ago. *Nature* **622**, 107–111 (2023).

5. H. Thieme, Lower Palaeolithic hunting spears from Germany. *Nature* **385**, 807–810 (1997).

6. N. J. Conard et al., Excavations at Schöningen and paradigm shifts in human evolution. *J Hum Evol* **89**, 1–17 (2015).

7. L. Allington-Jones, The Clacton spear: The last one hundred years. *Archaeol J* **172**, 273–296 (2015).

8. D. Mania, U. Mania, Geräte aus Holz von der altpaläolithischen Fundstelle bei Bilzingsleben. *Praehistoria Thuringica* **2**, 32–72 (1998).

9. Wagner, E. *Cannstatt I. Großwildjäger im Travertingebiet.* Forschungen und Berichte zur Vor-und Frühgeschichte in Baden-Württemberg 61 (Theiss, 1995).

10. W. H. Schoch, Holzanatomische Nachuntersuchungen an der eemzeitlichen Holzlanze von Lehringen, Ldkr. Verden. *Nachrichten aus Niedersachsens Urgeschichte* **83**, 19–29 (2014).

11. E. G. Puhar, L. Korat, M. Erič, A. Jaklič, F. Solina, Microtomographic Analysis of a Palaeolithic Wooden Point from the Ljubljanica River. *Sensors* **22**, 2369 (2022). https://doi.org/10.3390/s22062369

12. T. D. Dillehay, "*Monte Verde: A Late Pleistocene settlement in Chile. Volume 2: The archaeological context and interpretation*" (Smithsonian Institution Press, 1997).

13. J. D. Clark, "Modified, used and other wood specimens from Acheulean horizons" in *Kalambo Falls Prehistoric Site, Volume 3: The Earlier Cultures: Middle and Earlier Stone Age,* J. D. Clark, Ed. (Cambridge University Press, 2001), pp. 481–491.

14. N. J. Conard, J. Serangeli, G., Bigga, V. Rots, A 300,000-year-old throwing stick from Schöningen, northern Germany, documents the evolution of human hunting. *Nat Ecol Evol* **4**, 690–693 (2020).

15. A. Milks, A double-pointed wooden throwing stick from Schöningen, Germany: Results and new insights from a multianalytical study. *PLoS One* **18**(7), e0287719 (2023). https://doi.org/10.1371/journal.pone.0287719

16. M. K. Bamford, Z. L. Henderson, A reassessment of the wooden fragment from Florisbad, South Africa. *J Archaeol Sci* **30**, 637–650 (2003).

17. J., Meadows, C. Heron, M. Hüls, B. Philippsen, M. J. Weber, Dating the lost arrow shafts from Stellmoor (Schleswig-Holstein, Germany). *Quartär* **65**, 105–114 (2018).

18. X. Gao, *et al.*, 300,000-year-old wooden tools from Gantangqing, southwest China. Research Square [Preprint] (2021). https://doi.org/10.21203/rs.3.rs-226285/v1 (accessed 23 January 2024).

19. B. Aranguren, *et al.*, Wooden tools and fire technology in the early Neanderthal site of Poggetti Vecchi (Italy). *Proc Natl Acad Sci U S A* **115**, 2054–2059 (2018).

20. J. Rios-Garaizar, *et al.*, A Middle Palaeolithic wooden digging stick from Aranbaltza III, Spain. *PLoS One* **13**, e0195044 (2018). https://doi.org/10.1371/journal.pone.0195044

21. F. d'Errico *et al.*, Early evidence of San material culture represented by organic artifacts from Border Cave, South Africa. *Proc Natl Acad Sci U S A* **109**, 13214–13219 (2012).

22. H. Thieme, *Die Schöninger Speere. Mensch und Jagd vor 400 000 Jahren* (Theiss, 2007).

23. E. Carbonell, Z. Castro-Curel, Palaeolithic wooden artefacts from the Abric Romani (Capellades, Barcelona, Spain). *J Archaeol Sci* **19**, 707–719 (1992).

24. D. Nadel, U. Grinberg, E. Boaretto, E. Werker, Wooden objects from Ohalo II (23,000 cal BP), Jordan Valley, Israel. *J Hum Evol* **50**, 644–662 (2006).

25. T. Terberger, M. Zhilin, S. Savchenko, The Shigir idol in the context of early art in Eurasia. *Quat Int*, **573**, 14–29 (2021).

26. C. Richter, *Wood Characteristics. Description, Causes, Prevention, Impact on Use and Technological Adaptation* (Springer International Publishing, 2015).

27. O. Faix, Classification of lignins from different botanical origins by FT-IR spectroscopy. *Holzforschung* **45**, 21–28 (1991).

28. D. Fengel, Influence of water on the OH valency range in deconvoluted FTIR spectra of cellulose. *Holzforschung* **47**, 103–108 (1993).

29. K. K. Pandey, A study of chemical structure of soft and hardwood and wood polymers by FTIR spectroscopy. *J Appl Polym Sci* **71**(12), 1969–1975 (1999).

30. E. Smidt, J. Tintner, S. Klemm, U. Scholz, FT-IR spectral and thermal characterization of ancient charcoals: A tool to support archeological and historical data interpretation. *Quat Int* **457**, 43–49 (2017).

31. B. Smith, *Infrared spectral interpretation: A systematic approach* (CRC press, 1999).

32. S. M. Kwon, J. H. Jang, S. H. Lee, S. B. Park, N. H. Kim, Change of heating value, pH and FT-IR spectra of charcoal at different carbonization temperatures. *Journal of the Korean Wood Science and Technology* **41**(5), 440–446 (2013).

33. R. K. Sharma *et al.*, Characterization of chars from pyrolysis of lignin. Fuel **83**(11–12), 1469–1482 (2004).

34. Y. Yang *et al.*, Wood-burning processes in variable oxygen atmospheres: Thermolysis, fire, and smoke release behavior. *Polym Degrad Stab* **205**, 110158 (2022). https://doi.org/10.1016/j.polymdegradstab.2022.110158

35. W. H. Schoch, G. Bigga, U. Böhner, P. Richter, T. Terberger, New insights on the wooden weapons from the Paleolithic site of Schöningen, *J Hum Evol* **89**, 214–225 (2015).

36. M. Kot *et al.*, Frontiers of the Lower Palaeolithic expansion in Europe: Tunel Wielki Cave (Poland). *Sci Rep* **12**, 16355 (2022). https://doi.org/10.1038/s41598-022-20582-0

37. R. Rocca, First settlements in Central Europe: Between originality and banality, *Quat Int* **409**(B), 213–221 (2016).

38. O. V. Lozovskaya, V. M. Lozovski, "Mode de fabrication des outils en bois dans le Mésolithique d'Europe Orientale: Approche expérimentale-tracéologique" in *Experimentación en arqueología: Estudio y difusión del pasado. Serie Monografica del MAC-Girona 25.1*, A. Palomo, R. Pique, X. Terradas, Eds. (Museu d'Arqueologia de Catalunya, 2013), pp. 73–83.

39. M. M. Airaksinen *et al.*, Toxicity of plant material used as emergency food during famines in Finland. *J Ethnopharmacol* **18**(3), 273–296 (1986).

40. A. Milks et al., Wood technology: A glossary and code for the analysis of archaeological wood from stone tool cultures. OSF Preprints [Preprint] (2022). https://osf.io/x8m4j/ (accessed 23 January 2024).

41. R. E. Biermann Gürbüz, S. J. Lycett, Asymmetrical Paleolithic wooden spear tips: Expediency or design? *J Archaeol Sci Rep* **30**, 102209 (2020). https://doi.org/10.1016/j.jasrep.2020.102209

42. A. R. Ennos, T. L. Chan, 'Fire hardening' spear wood does slightly harden it, but makes it much weaker and more brittle. *Biol Lett* **12**, 20160174 (2016). http://dx.doi.org/10.1098/rsbl.2016.0174

43. A. Milks, *Lethal Threshold: The Evolutionary Implications of Middle Pleistocene Wooden Spears* (PhD Thesis, University College of London, 2018). Available from: https://discovery.ucl.ac.uk/id/eprint/10045809/1/MILKS%20PhD%20Final.compressed%20no%203%20party.pdf (accessed 23 January 2024).

44. N. Macchioni, C. Capretti, L. Sozzi, B. Pizzo, Grading the decay of waterlogged archaeological wood according to anatomical characterisation: The case of the Fiavé site (N-E Italy). *Int Biodeterior Biodegradation* **84**, 54–64 (2013).

45. U. Schmitt, A. P. Singh, H. Thieme, P. Friedrich, P. Hoffmann, Electron microscopic characterization of cell wall degradation of the 400,000-year-old wooden Schöningen spears. *Holz Roh Werkst* **63**, 118–122 (2005).

46. S. Weiss, K. Urdl, H. A. Mayer, E. M. Zikulnig-Rusch, A. Kandelbauer, IR spectroscopy: Suitable method for determination of curing degree and crosslinking type in melamine–formaldehyde resins. *J Appl Polym Sci* **136**(25), 47691 (2019).

47. A. Factor, "Char formation in aromatic engineering polymers" in *Fire and Polymers. Hazards, Identification and Prevention, ACS Symposium Series 425*, G.L. Nelson, Ed. (American Chemical Society ,1990), pp. 274-287.

48. D. B. Bamforth, Technological efficiency and tool curation. *Am Antiq* **51**(1), 38-50 (1986).

49. B. Urban *et al.*, Landscape dynamics and chronological refinement of the Middle Pleistocene Reinsdorf Sequence of Schöningen, NW Germany. *Quat Res* **114**, 148–177 (2023).

50. L. R. Binford, Organization and formation processes: Looking at curated technologies. *J Anthropol Res* **35**(3), 255–273 (1979).

51. A. Milks, C., Hoggard, M. Pope, Reassessing the interpretative potential of ethnographic collections for early hunting technologies. *J Archaeol Method Theory* (2023). https://doi.org/10.1007/s10816-023-09635-4

52. M. R. Bebber, B. Buchanan, M. I. Eren, R. S. Walker, D. Zirkle, Atlatl use equalizes female and male projectile weapon velocity. *Sci Rep* **13**(1), 13349 (2023). https://doi.org/10.1038/s41598-023-40451-8

53. A. L. Lockey, et al., Comparing the Boxgrove and Atapuerca (Sima de los Huesos) human fossils: Do they represent distinct paleodemes? *J Hum Evol* **172**, 103253 (2022). https://doi.org/10.1016/j.jhevol.2022.103253

54. R. Davis, N. Ashton, Landscapes, environments and societies: The development of culture in Lower Palaeolithic Europe. *J Anthropol Archaeol* **56**, 101107 (2019). https://doi.org/10.1016/j.jaa.2019.101107

55. J.A. Burdukiewicz, The Lower Palaeolithic assemblages in Central Europe in stratigraphic and palaeogeographic background. *Anthropologie* **125**(4), 102937 (2021). https://doi.org/10.1016/j.anthro.2021.102937

56. K. Zutovski, R. Barkai, The use of elephant bones for making Acheulian handaxes: A fresh look at old bones, *Quat Int* **406**(B), 227-238 (2016).

57. J. Serangeli, U. Böhner, „Die Steinartefakte von Schöningen und deren zeitliche Einordnung“ in *Die chronologische Einordnung der paläolithischen Fundstellen von Schöningen*, K.-H. Behre, Eds. Forschungen zur Urgeschichte aus dem Tagebau Schöningen, Volume 1 (Verlag des RGZM, 2012), pp. 23 –38.

58. J. E. Fairlie, L. S. Barham, From *Chaîne Opératoire* to observational analysis: A pilot study of a new methodology for analysing changes in cognitive task-structuring strategies across different hominin tool-making events. *Cambridge Archaeological Journal* **26**(4), 643–664 (2016).

59. R. Wragg Sykes, “To see a world in a hafted tool: Birch pitch composite technology, cognition and memory in Neanderthals” in *Settlement, society and cognition in human evolution,* F. Coward, R. Hosfield, M. Pope, F. Wenban-Smith, Eds. (Cambridge University Press, 2015), pp. 117–137.

60. M. Lombard, M. N. Haidle, 2012. Thinking a bow-and-arrow set: Cognitive implications of Middle Stone Age bow and stone-tipped arrow technology. *Cambridge Archaeological Journal* **22**(2), 237–264 (2012).

61. P. J. Richerson, R. Boyd, *Not by genes alone: How culture transformed human evolution* (University of Chicago Press, 2005).

62. M. Haidle, *How to think tools? A comparison of cognitive aspects in tool behavior of animals and during human evolution* (Universitätsbibliothek Tübingen, 2012).

63. G. B. Tostevin, *Seeing lithics: A middle-range theory for testing for cultural transmission in the Pleistocene.* American School of Prehistoric Research Monograph Series, Peabody Museum, Harvard University (Oxbow Books, 2012).

64. M. J. L. T. Niekus et al., Middle Paleolithic complex technology and a Neandertal tar-backed tool from the Dutch North Sea. *Proc Natl Acad Sci U S A* **116**(44), 201907828 (2019).

65. P. Schmidt *et al.*, Production method of the Königsaue birch tar documents cumulative culture in Neanderthals. *Archaeol Anthropol Sci* **15**, 84 (2023). https://doi.org/10.1007/s12520-023-01789-2

66. V. Rots, P. Van Peer, Early evidence of complexity in lithic economy: core-axe production, hafting and use at Late Middle Pleistocene site 8-B-11, Sai Island (Sudan). *J Archaeol Sci* **33**(3), 360–371. (2006).

67. J. Wilkins, B. J. Schoville, K. S. Brown, M. Chazan, Evidence for early hafted hunting technology. *Science* **338**, 942–946 (2012).

68. V. Rots, B. L. Hardy, J. Serangeli, N. J. Conard, Residue and microwear analyses of the stone artifacts from Schöningen. *J Hum Evol* **89**, 298–308 (2015). https://doi.org/10.1016/j.jhevol.2015.07.005

69. P. P. A. Mazza *et al.*, A new Palaeolithic discovery: Tar-hafted stone tools in a European Mid-Pleistocene bone-bearing bed. *J Archaeol Sci* **33**(9), 1310–1318 (2006).

70. V. Rots, Insights into early Middle Palaeolithic tool use and hafting in Western Europe: The functional analysis of level IIa of the early Middle Palaeolithic site of Biache-Saint-Vaast (France). *J Archaeol Sci* **40**, 497–506 (2013).

71. L. Wadley, T. Hodgskiss, M. Grant, Implications for complex cognition from the hafting of tools with compound adhesives in the Middle Stone Age, South Africa. *Proc Natl Acad Sci U S A* **106**(24), 9590–9594 (2009).

72. E. Boëda, *et al.*, Middle Palaeolithic bitumen use at Umm el Tlel around 70 000 BP. *Antiquity* **82**(318), 853–861 (2008).

73. J. J. Shea, Lithic use-wear evidence for hunting by Neanderthals and early modern humans from the Levantine Mousterian. *Archeological Papers of the American Anthropological Association* **4**(1), 189–197 (1993).

74. L. R. Backwell, F. d'Errico, The first use of bone tools: a reappraisal of the evidence from Olduvai Gorge, Tanzania. *Palaeontologia africana* **40**(9), 95–158 (2004).

75. L. R. Backwell, F. d'Errico, Evidence of termite foraging by Swartkrans early hominids. *Proc Natl Acad Sci U S A* **98**(4), 1358–1363 (2001).

76. M. Pante, I. de la Torre, F. d'Errico, J. Njau, R. Blumenschine, Bone tools from Beds II–IV, Olduvai Gorge, Tanzania, and implications for the origins and evolution of bone technology. *J Hum Evol* **148**, 102885 (2020). https://doi.org/10.1016/j.jhevol.2020.102885

77. L. S. Barham, A. C. Pinto Llona, C. B. Stringer, Bone tools from Broken Hill (Kabwe) cave, Zambia, and their evolutionary significance. *Before farming* **2**(3), 1-12 (2002).

78. J. Rosell, *et al.*, Bone as a technological raw material at the Gran Dolina site (Sierra de Atapuerca, Burgos, Spain), *J Hum Evol* **61**(1), 125-131 (2011).

79. L. Steguweit, *Gebrauchsspuren an Artefakten der Hominidenfundstelle Bilzingsleben (Thüringen). Tübinger Arbeiten zur Urgeschichte 2* (Verlag Marie Leidorf, Rahden, 2003).

80. S. A. Parfitt, S. M. Bello, Bone tools, carnivore chewing and heavy percussion: Assessing conflicting interpretations of Lower and Upper Palaeolithic bone assemblages. *R Soc Open Sci* **11**(1), 231163 (2024). https://doi.org/10.1098/rsos.231163

81. R. Blasco, J. Rosell, A. Gopher, R. Barkai, Subsistence economy and social life: A zooarcheological view from the 300 kya central hearth at Qesem Cave, Israel. *J Anthropol Archaeol* **35**, 248–268 (2014).

82. N. Goren-Inbar, "Behavioral and cultural origins of Neanderthals: A Levantine perspective" in *Continuity and Discontinuity in the Peopling of Europe. One Hundred Fifty Years of Neanderthal Study* S. Condemi, G.-C. Weniger, Eds. (Springer, 2011), pp. 89–100.

83. M.-A. Julien *et al.*, Characterizing the Lower Paleolithic bone industry from Schöningen 12 II: A multi-proxy study, *J Hum Evol* **89,** 264-286 (2015).

84. T. van Kolfschoten, S. A. Parfitt, J. Serangeli, S. M. Bello, Lower Paleolithic bone tools from the 'Spear Horizon' at Schöningen (Germany). *J Hum Evol* **89**, 226–263 (2015).

85. A.-M. Moigne *et al.*, Bone retouchers from Lower Palaeolithic sites: Terra Amata, Orgnac 3, Cagny-l'Epinette and Cueva del Angel. *Quat Int* **409**(B), 195–212 (2016).

86. S. Veil, Die Nachbildung der Lanze von Lehringen: Experimente zur Holzbearbeitung im Mittelpaläolithikum. *Die Kunde* **41/42**, 9–22 (1991).

87. T. Broekel, Measuring technological complexity: Current approaches and a new measure of structural complexity. arXiv [Preprint] (2018). https://doi.org/10.48550/arXiv.1708.07357 (accessed 23 January 2024).

88. C. Perreault, P. J. Brantingham, S .L. Kuhn, S. Wurz, X. Gao, Measuring the Complexity of Lithic Technology. *Curr Anthropol* **54**(S8), S397-S406.

89. S. H. Ambrose, Coevolution of composite tool technology, constructive memory, and language. *Curr Anthropol* **51**(suppl.1), S135–S147 (2010).

90. S. A. de Beaune, The invention of technology: Prehistory and cognition. *Curr Anthropol* **45**(2), 139–162 (2004).

91. W. H. Oswalt, *An anthropological analysis of food-getting technology* (Wiley-Inter science, 1976).

92. S. Fajardo, P. R. B. Kozowyk, G. H. J. Langejans, Measuring ancient technological complexity and its cognitive implications using Petri nets. arXiv [Preprint] (2023) https://doi.org/10.48550/arXiv.2305.0975 (accessed 23 January 2024).

93. N. Buc, Experimental series and use-wear in bone tools. *J Archaeol Sci* **38**(3), 2011, 546–557.

94. M. Soressi *et al.*, Neandertals made the first specialized bone tools in Europe. *Proc Natl Acad Sci U S A*, **110**(35), 14186–14190 (2013).

95. S. Arrighi, M. Bazzanella, F. Boschin, U. Wierer, How to make and use a bone "spatula": An experimental program based on the Mesolithic osseous assemblage of Galgenbühel/Dos de la Forca (Salurn/Salorno, BZ, Italy). *Quat Int* **423**, 143–165 (2016).

96. Thieme, H., 1999. Altpaläolithische Holzgeräte aus Schöningen, Lkr. Helmstedt: Bedeutsame Funde zur Kulturentwicklung des frühen Menschen. *Germania* **77**(2), 451–487 (1999).

97. B. L. Hardy *et al.*, Direct evidence of Neanderthal fibre technology and its cognitive and behavioral implications. *Sci Rep* **10**, 4889 (2020). https://doi.org/10.1038/s41598-020-61839-w

SI Figures

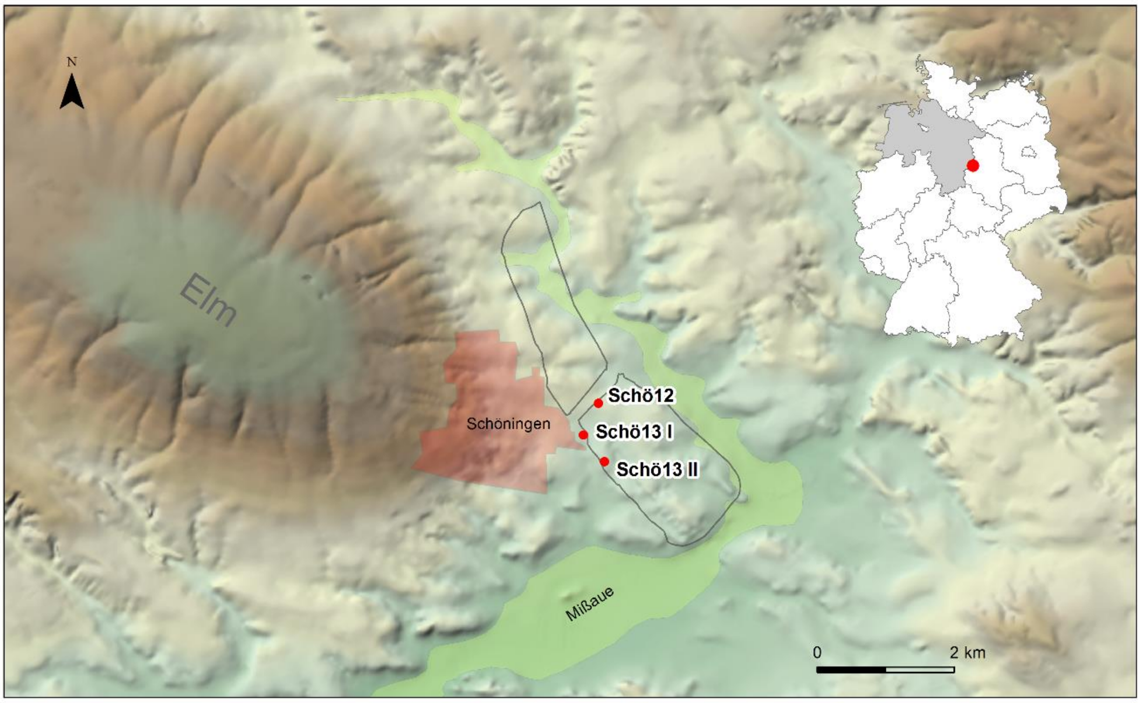

Fig. S1. Map of Schöningen in Lower Saxony, Germany, with Middle Pleistocene sites mentioned in the text (Schö= Schöningen). Note the Elm Mountain west of the Schöningen sites. The former mining area is enclosed with a black line. Map: Published in Journal of Human Evolution 89, 2015, Figure 1, p. 203. Original image created by one of us (Utz Böhner).

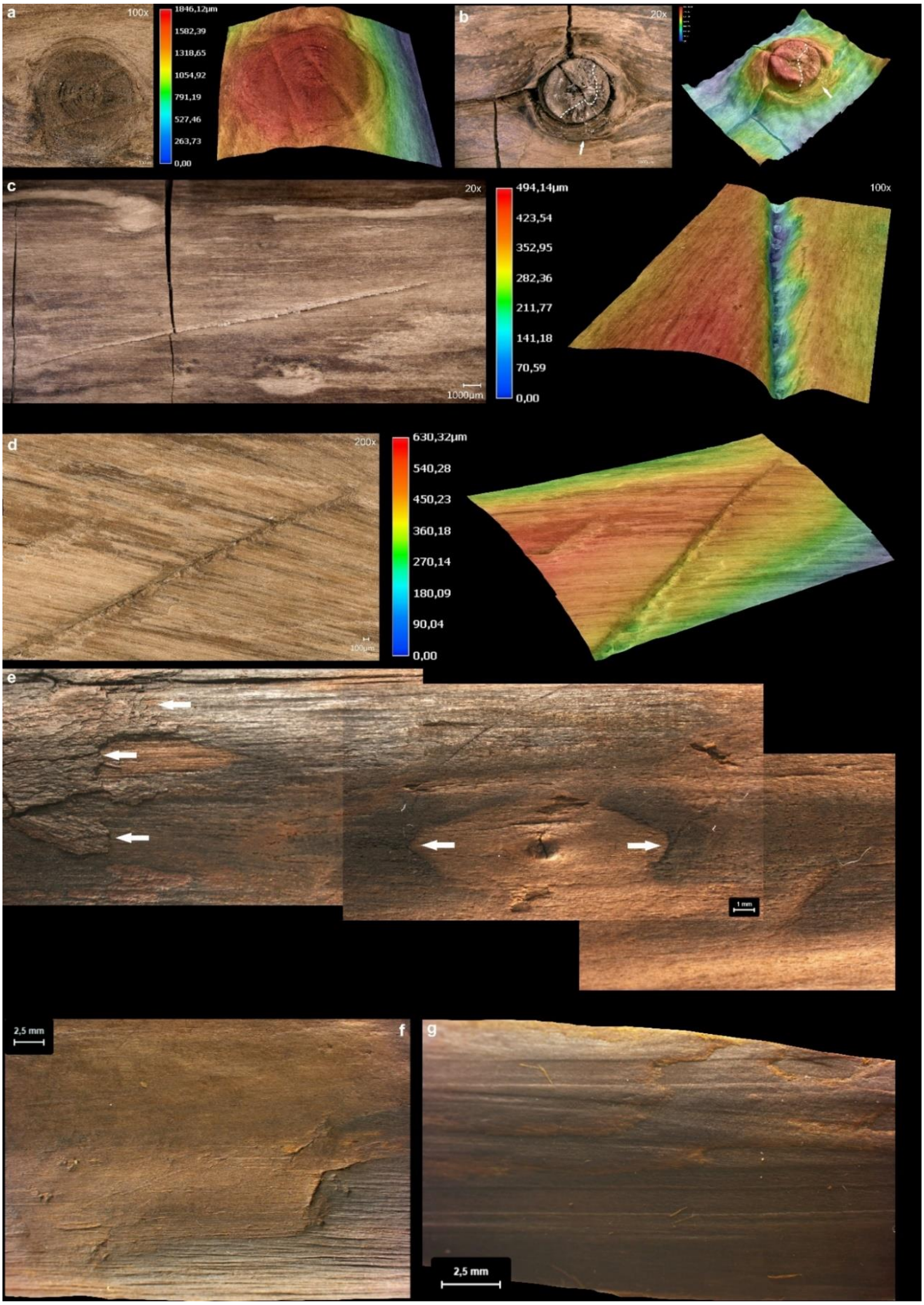

Fig. S2. Various working traces. **a** Cut off knot with scrapping marks going across it. **b** Broken off and partially abraded knot. **c-d** Oblique cut marks. **e** Cambium with debarked areas. Arrows show the working direction. **f**. Close-up view of a smoothed debarked area and cambium area with scraping marks. The stop mark indicates the direction of working. **g** Scraping marks, some parallel, some crossing. The steps indicate the direction of working. Photos: **a-d** Keyence VHX5000, Tim Koddenberg, Göttingen, Jens Lehmann, NLD; **e-g** Leica S9D, Flexacam C3, Jens Lehmann, Dirk Leder, NLD.

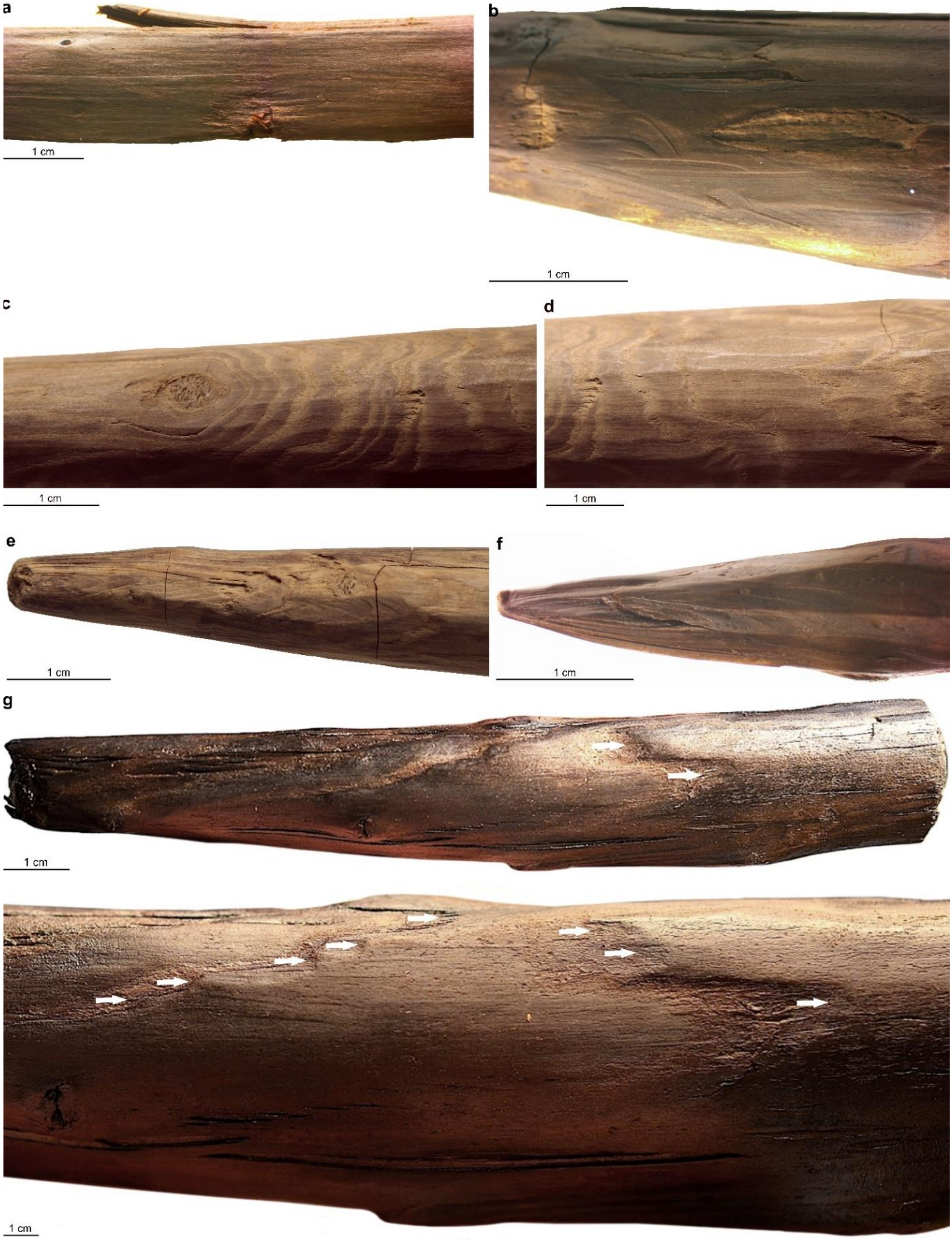

Fig. S3. Various working traces. **a** Scraping marks, some parallel, some crossing. **b** Tool facets with side features and signatures. **c-f** Surface facets on spear points. **g** Reworked point fragment (ID 4236). Stop marks indicate the reworked parts. The originally worked surface is much smoother and was likely abraded. Photos: **a-b** Leica S9D, Flexacam C3, Jens Lehmann, Dirk Leder, NLD. **c-g** Nikon D7000, Jens Lehmann, NLD.

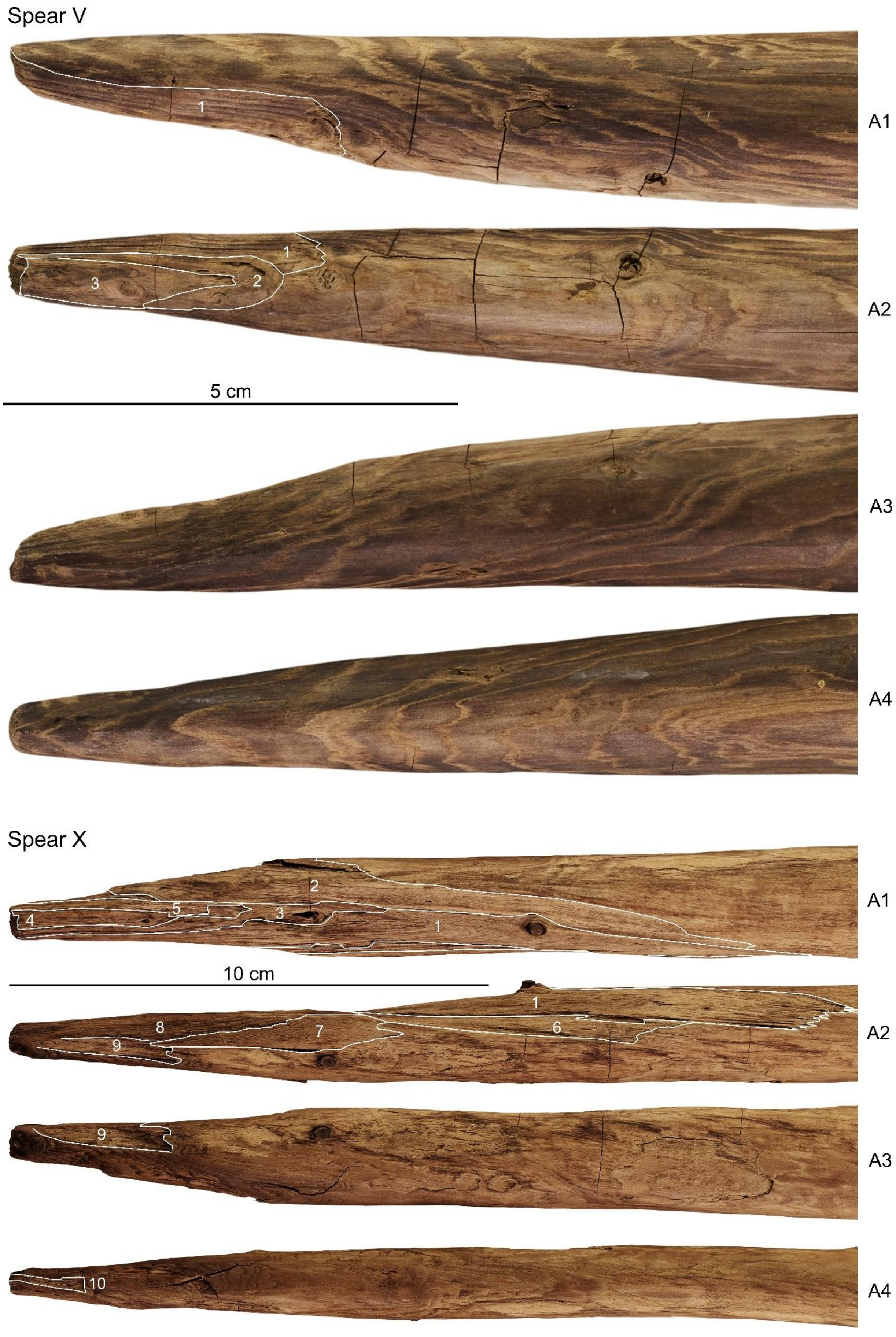

Fig. S4. Reworked front points of Spear V and Spear X in the four standard view A1 to A4. Numbers indicate the succession of removals. Photos: Minkusimages.

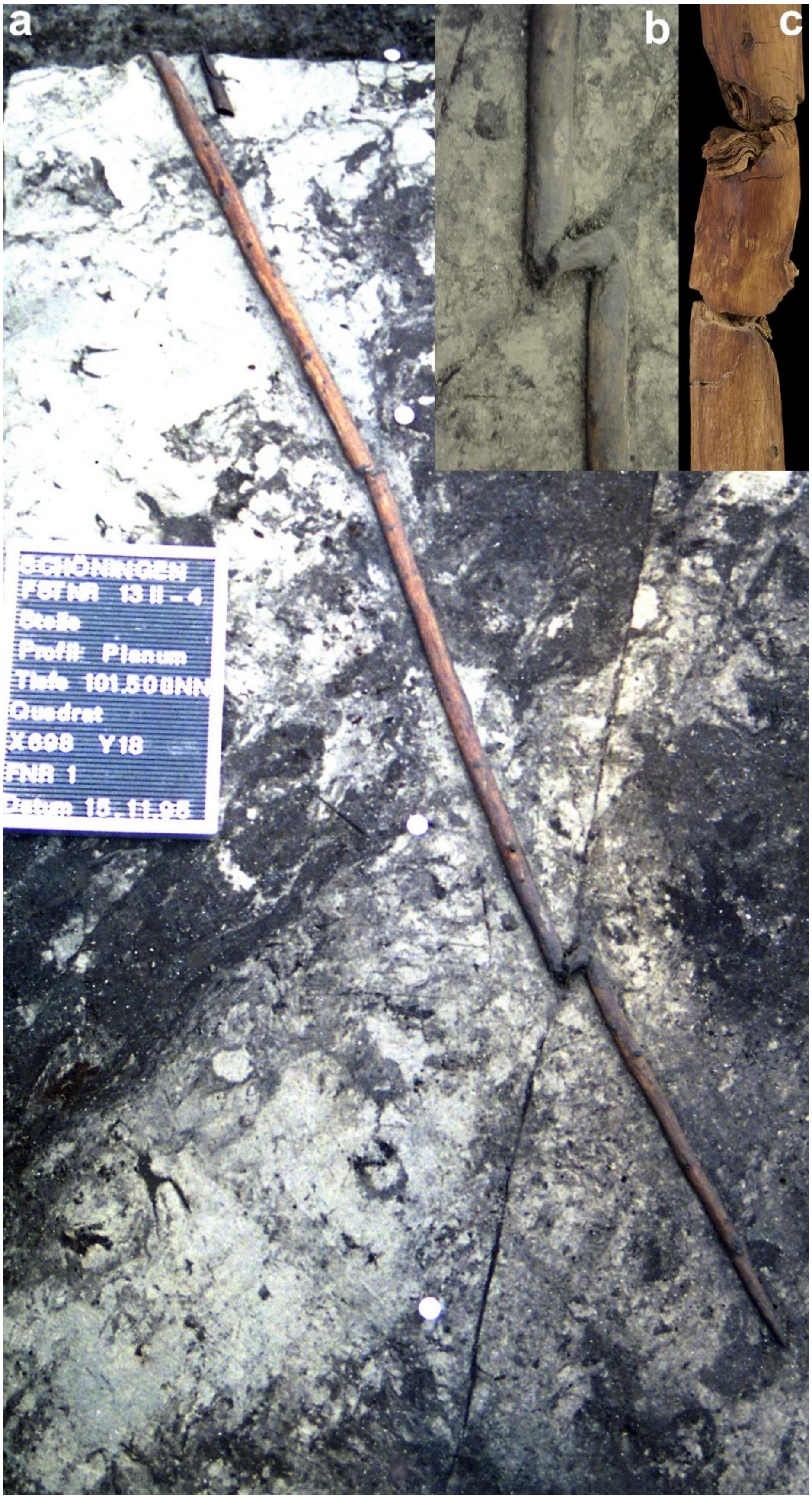

Fig. S5. Taphonomic fracture on Spear III. **a** Spear III during excavation. The taphonomic fracture at 1/3 of the shaft length is caused by moving sediment blocks as indicated by the long crack in the sediment. **b** Detail of the fracture during excavation. **c** Detail of the fracture after conservation. Photos: **a-b** Peter Pfarr, NLD. **c** Minkusimages.

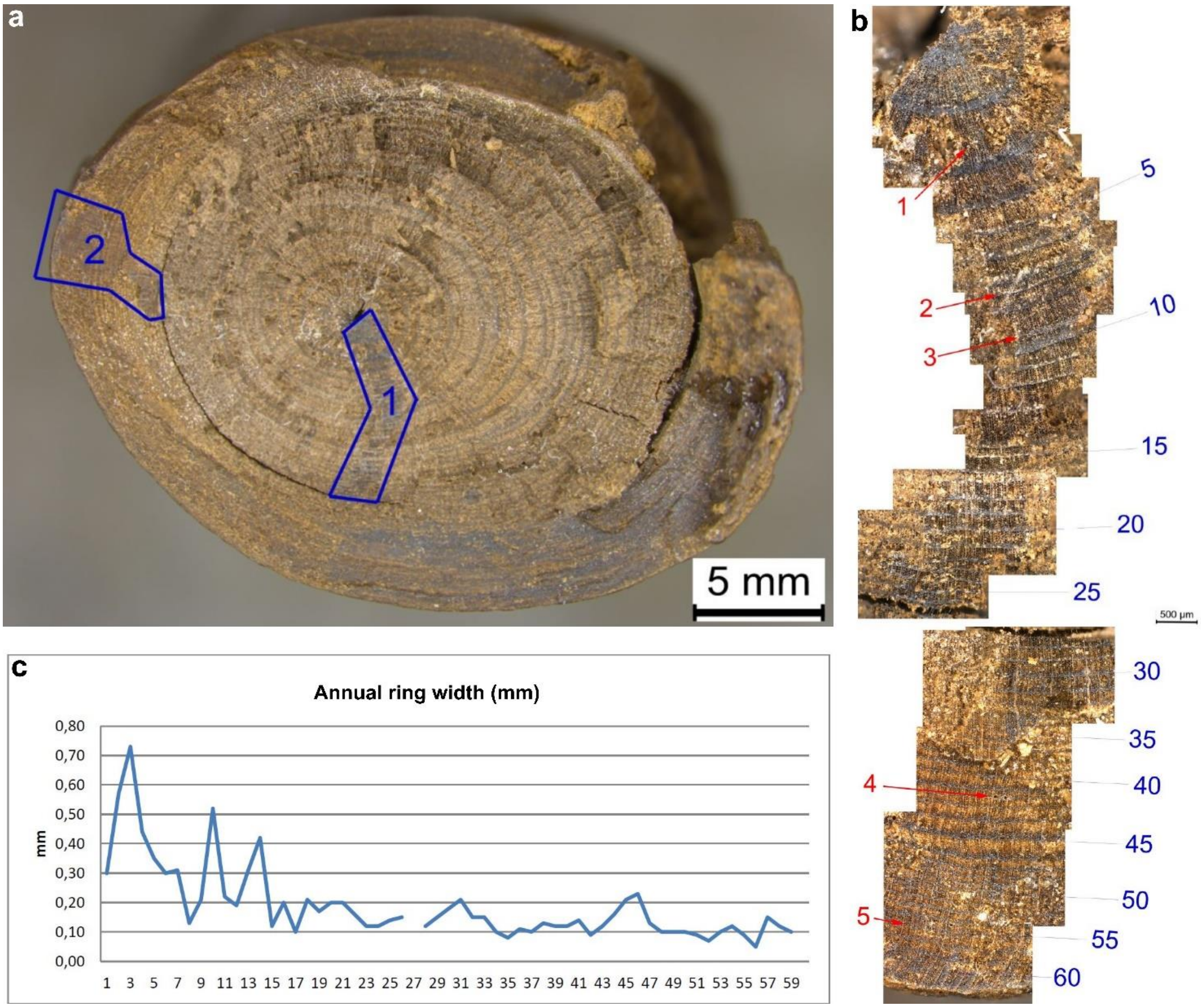

Fig. S6. Annual ring analysis on ID 1779 (double-pointed stick, DPS). **a** Taphonomic transverse break surface, from where micro-cuts were taken. **b** Composite image of different selections of the cross-section micro-cuts (corresponding to Area 1 on the left image). Blue numbers indicate the annual ring. Red arrows highlight features mentioned in the text. **c** Graph showing measurements (mm) of each annual ring. Published in PlosOne 18(7): e0287719. Figure 5. Original image created by one of us (Michael Sietz).

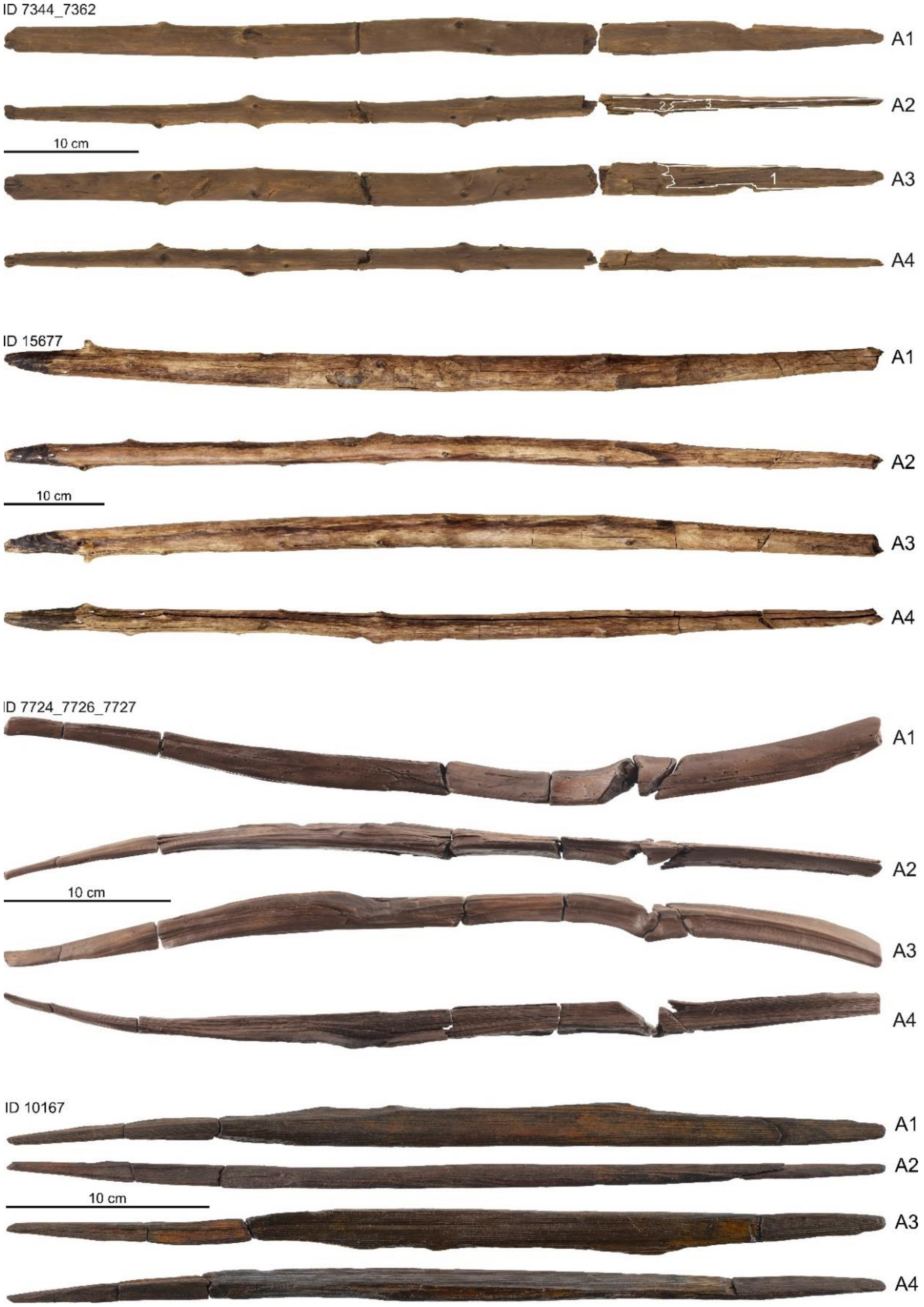

Fig. S7. Reworked points on double-pointed sticks (DPSs) and DPSs on split woods in the four standard view A1 to A4. ID 7344_7362 – The left point is broken off, the right point was longitudinally split in three steps. Numbers indicate the succession of removals. ID 15677 – The left point is reworked altering the point length and profile. The blackened point evidences exposure to fire. White arrows indicate stop marks that result from the reworking step (see Fig. S8). ID 7724_7726_7727 – DPS on split wood with one debarked surfaces, all other surfaces are split and smoothed including the points. ID 10167 – DPS on split wood with all surfaces split and smoothed, including the points. Photos: Christa S. Fuchs, Matthias Vogel, NLD.

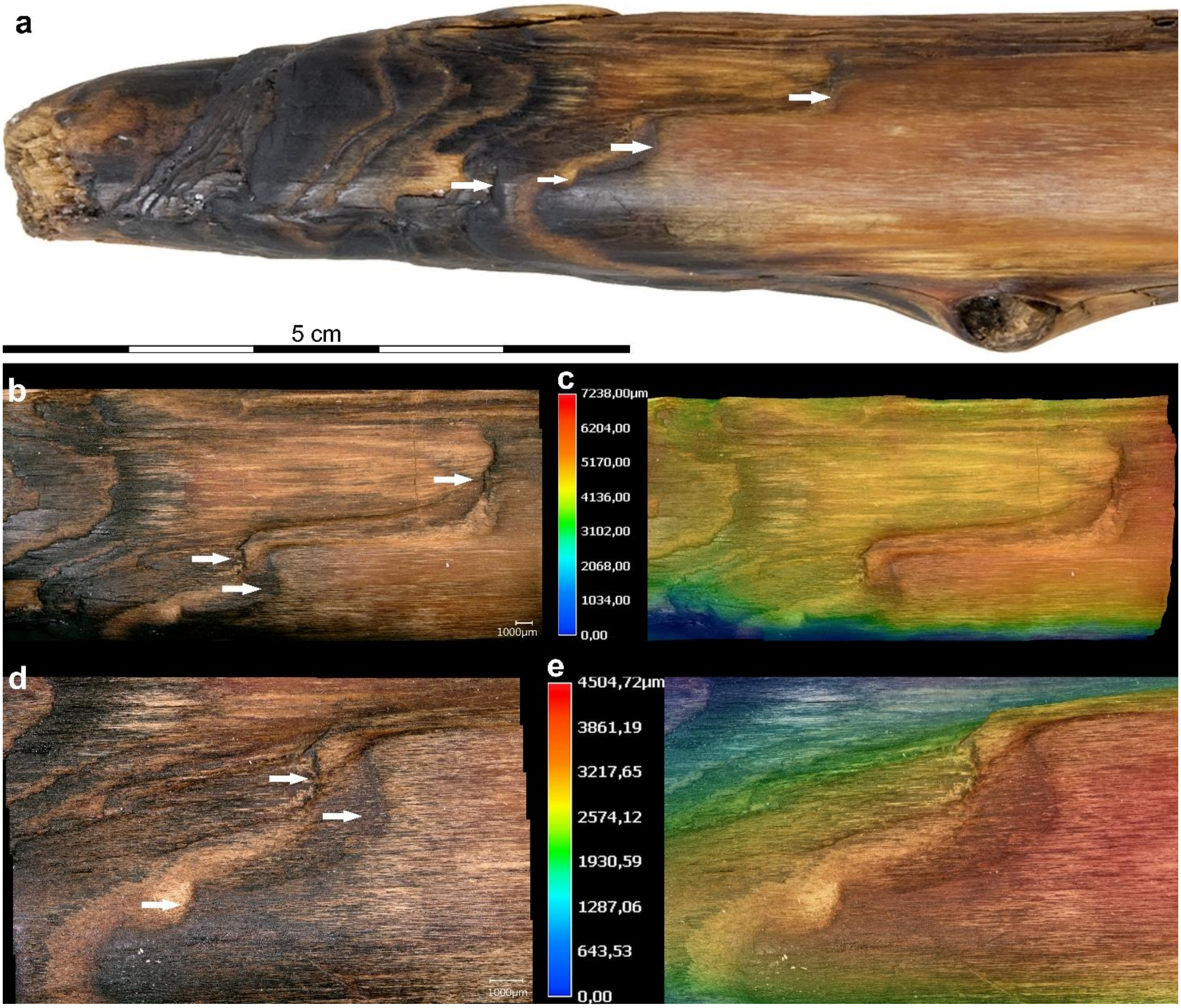

Fig. S8. The reworked point of ID 15677 (double-pointed stick, DPS). White arrows indicate stop marks that result from the reworking step. The black staining covering the point probably results from fire exposure. The very tip is altered taphonomically and shows charring is not very deep. Photos: **a** Nikon D200, Christa S. Fuchs, NLD; **b-d** VHX 5000, Tim Koddenberg, Göttingen, Jens Lehmann, NLD.

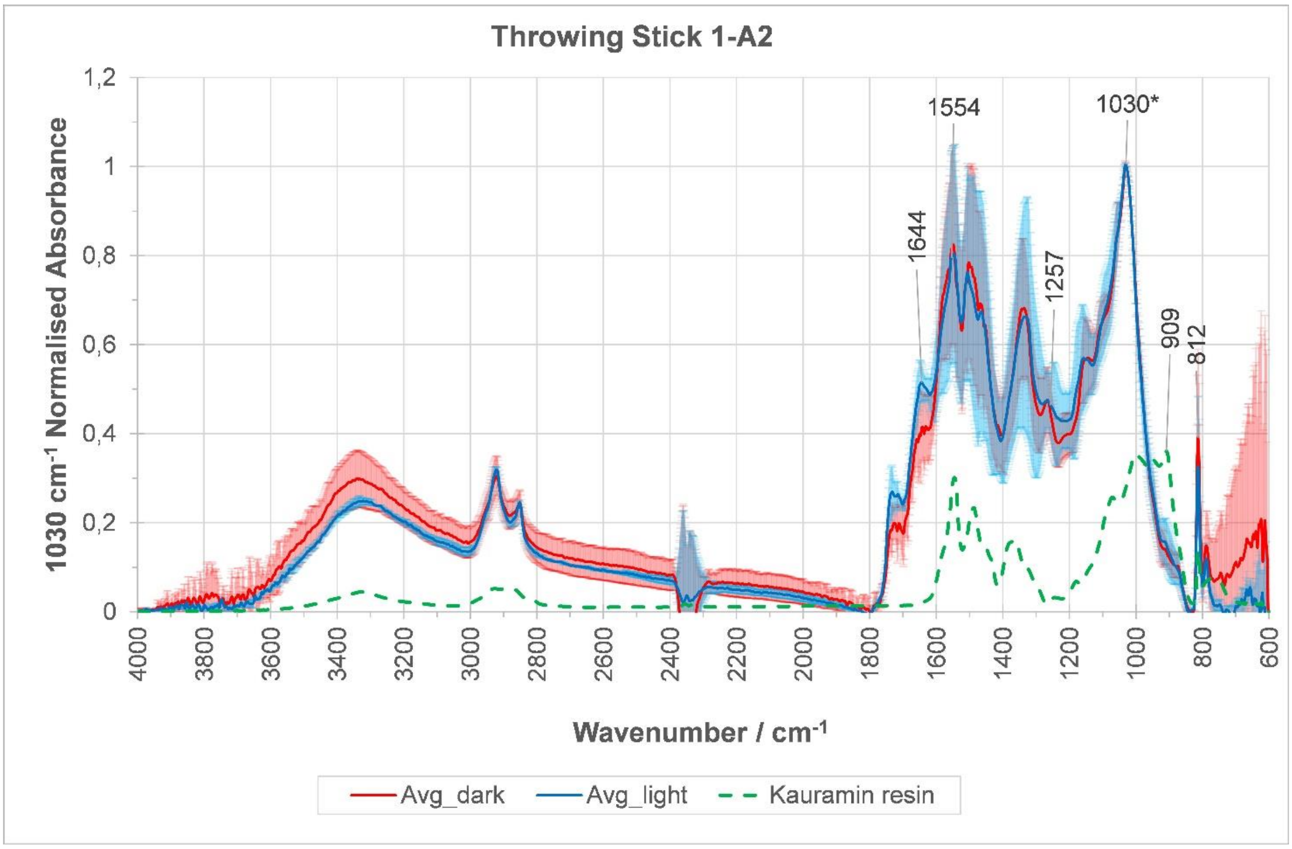

Fig. S9. ATR-IR results for averaged dark areas (Avg_dark) and light areas (Avg_light) of throwing stick (ID 1779) and their respective error bars as standard deviation of absorbances of investigated measurement positions. Additional curve of the neat Kauramin resin that had been used to conserve the artefact. Normalisation to absorbance at 1030 cm-1 except for Kauramin. Published in PlosOne 18(7): e0287719. SI Figure 11. Original image created by a former co-author (Volker Wachtendorf).

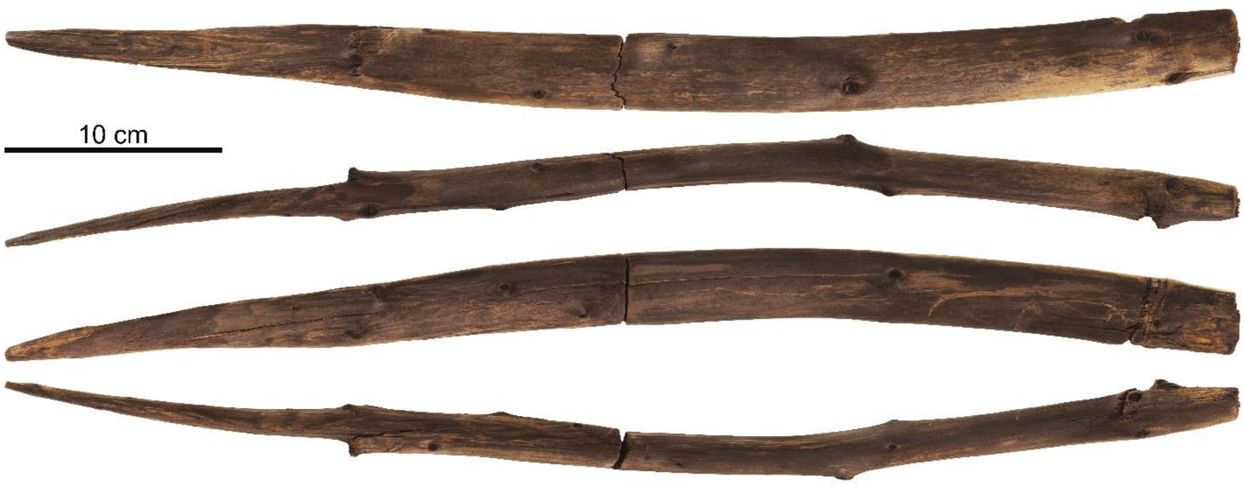

Fig. S10. Point fragment (ID 14852) with a longitudinally split tip coming from the left. The splitting mark ends after 15 cm at a knot and was smoothed thereafter. Photos: Nikon D200, Christa Fuchs, NLD.

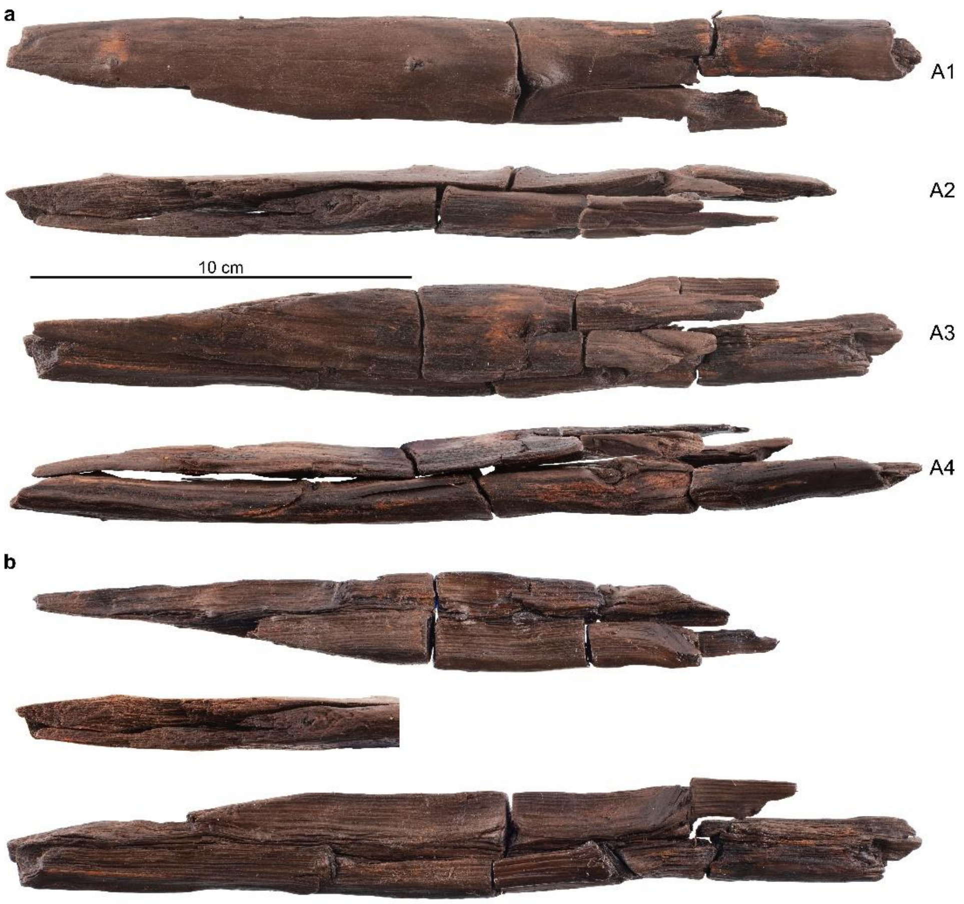

Fig. S11. The diagonally cut shaft fragment ID 8988 was later split in half along the pith. **a** Standard views A1 to A4 of the complete artefact. **b** Split surfaces of the same item. Taphonomic deterioration led to further fragmentation of the item. Photos: Nikon D850, Matthias Vogel, NLD.

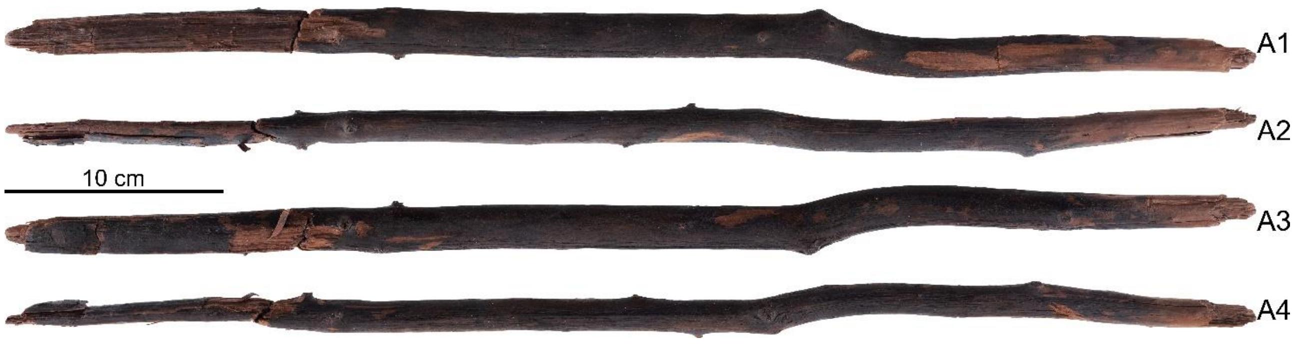

Fig. S12. Shaft fragment ID 7107_7108. The left part of the shaft was tangentially split and the resulting splitting mark smoothed. The purpose of this remains unclear. Photos: Nikon D850, Matthias Vogel, NLD.

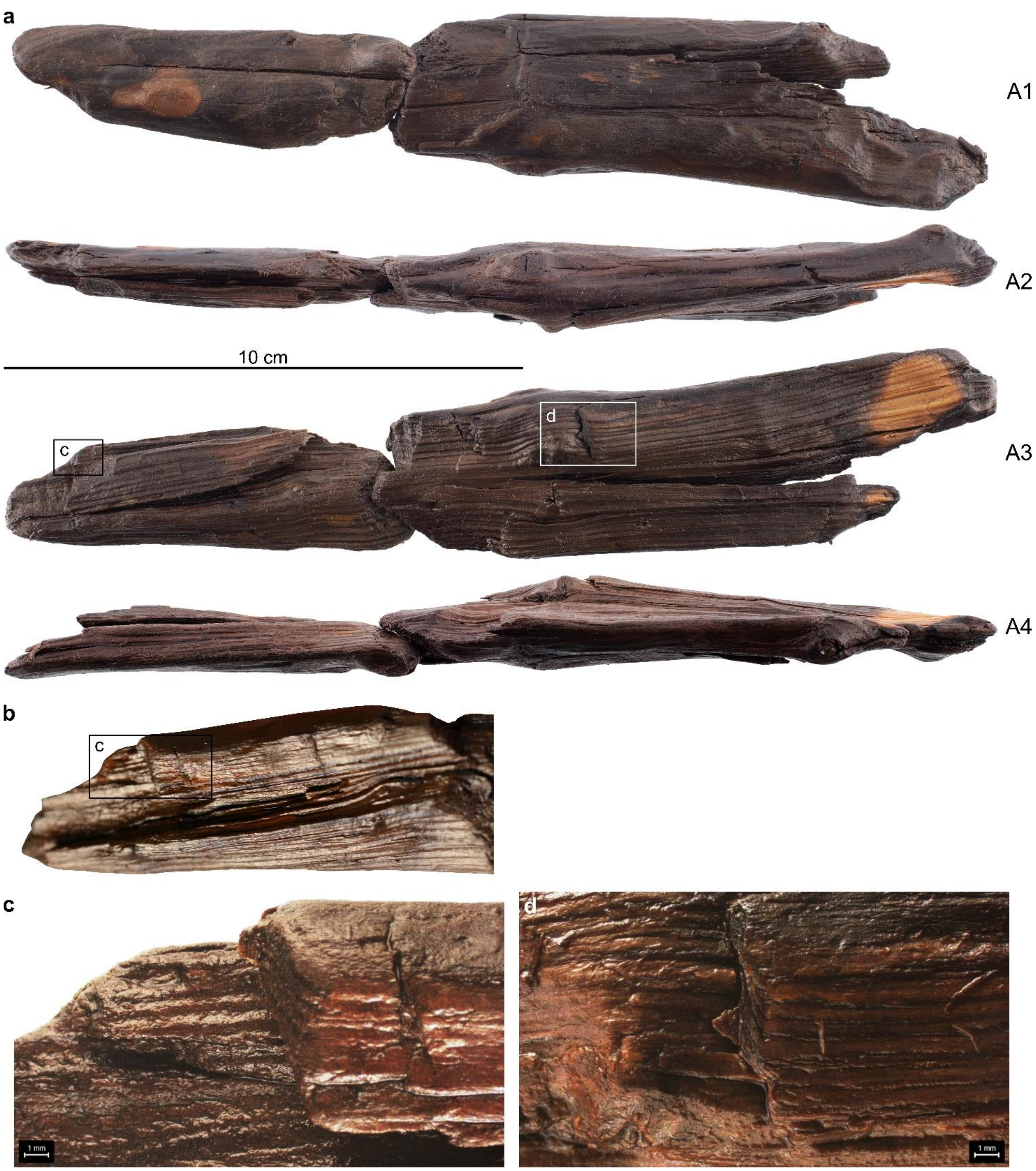

Fig. S13. Shaft fragment (ID 11125) with splinter removals. **a** Standard views A1 to A4. Areas in boxes are detailed in images **c-d**. The splinter removal in box **c** is covered by a wood chip (compare **b**). **b** Left part of the artefact without the wood chip, now showing the area highlighted in **c**. **c-d** Splinters were extracted from the split surface as perpendicular cut marks indicate. Photos: **a** Nikon D850, Matthias Vogel. **b** Nikon D7000, Jens Lehmann. **c-d** Leica S9D, Flexacam C3, Dirk Leder. All NLD.

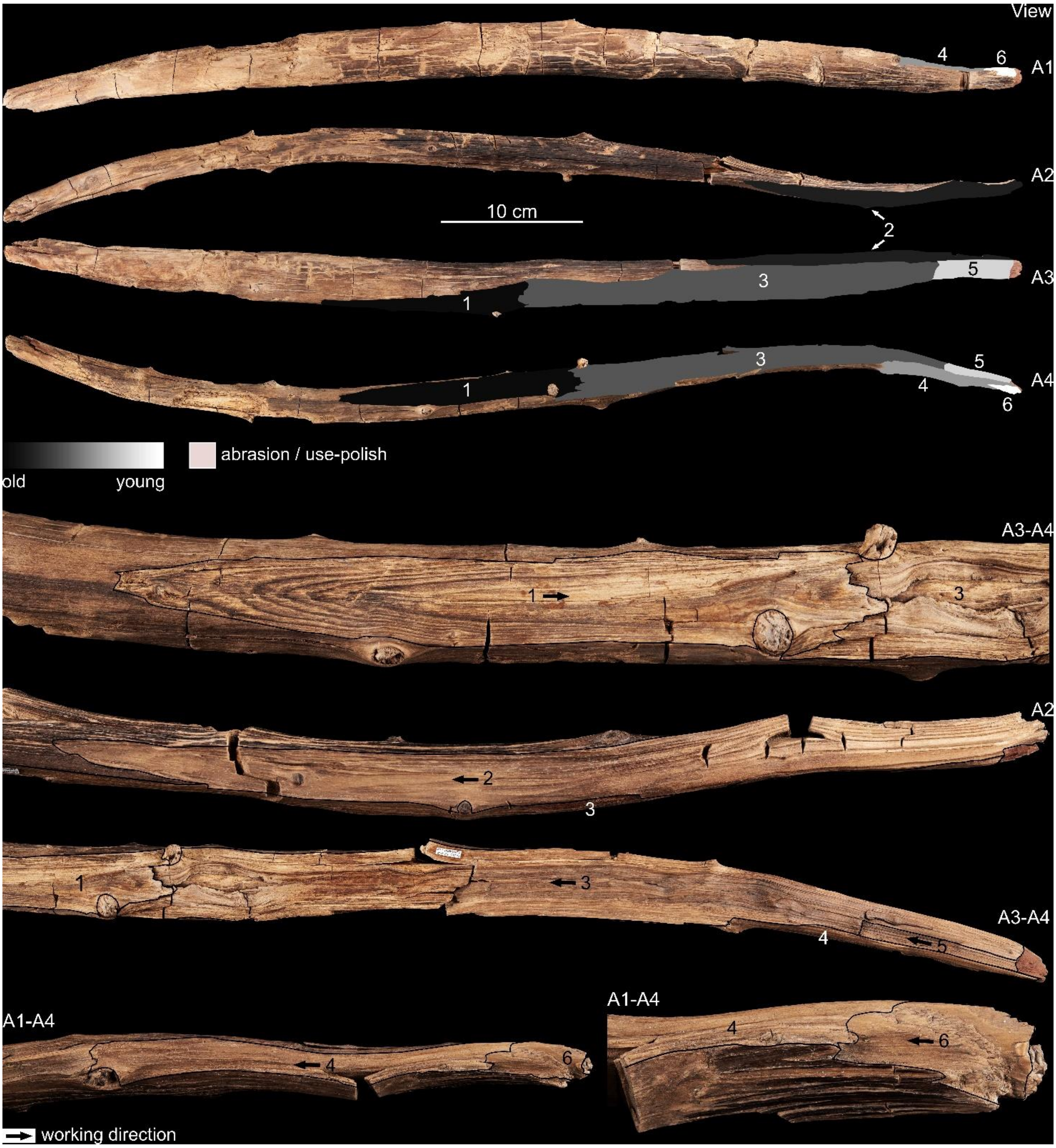

Fig. S14. Shaft fragment with rounded(?) tool end (ID 11383_12886). Six splinters were extracted from the right end of the artefact as splitting marks show. Photos: Nikon D850, Matthias Vogel, NLD.

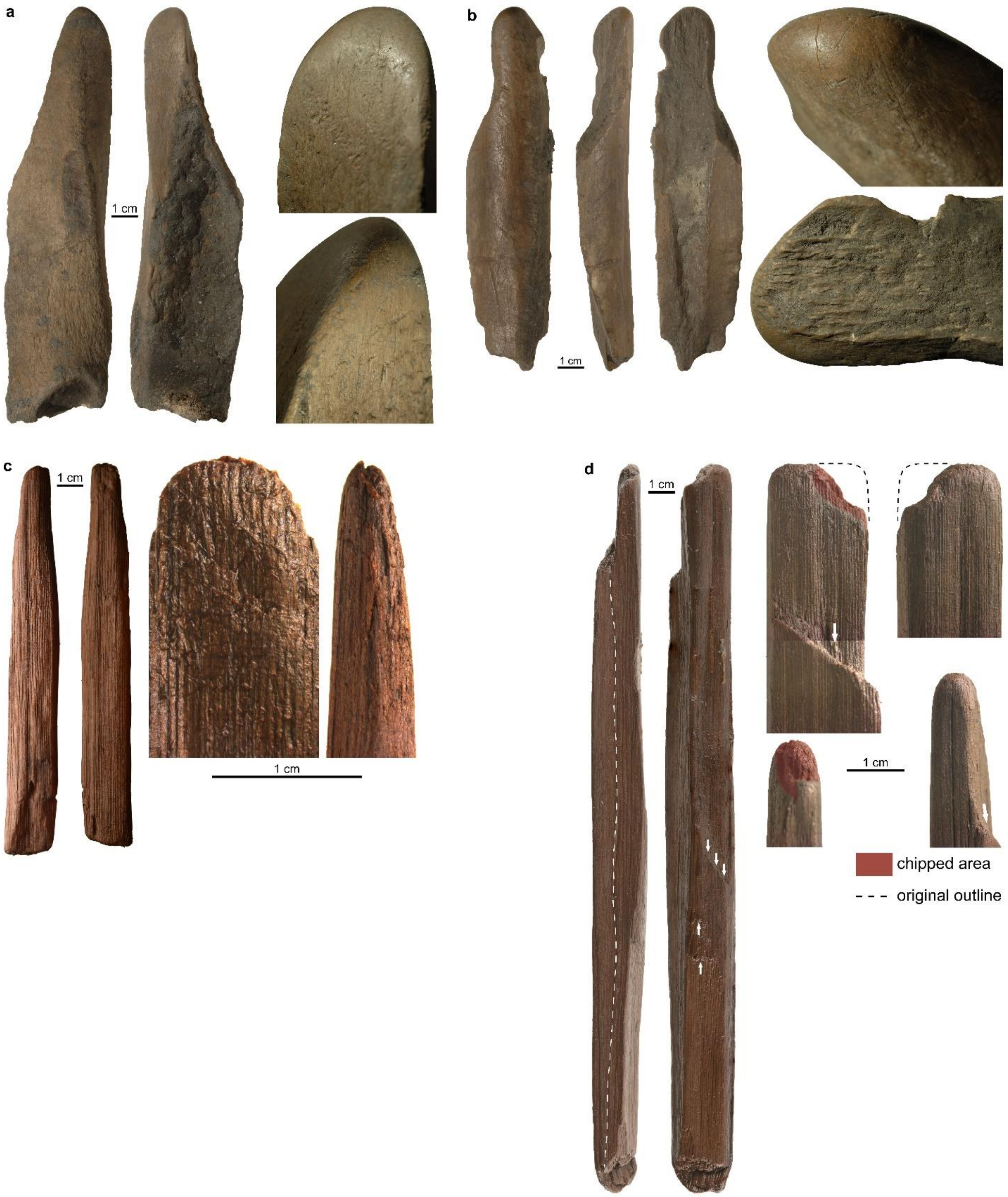

Fig. S15. Comparison between hide smoothers from Schöningen 12 II made from bone (**a-b**) and round-ended tools from Schöningen 13 II-4 made from wood (**c-d**). Photos: **a-b** Nikon D200, Christa Fuchs. **c** Overview photos with Nikon D200, Pascale Richter. **d** Overview photos with Nikon D850, Matthias Vogel. **c-d** Details with Leica S9D, Flexacam C3, Dirk Leder. All NLD.

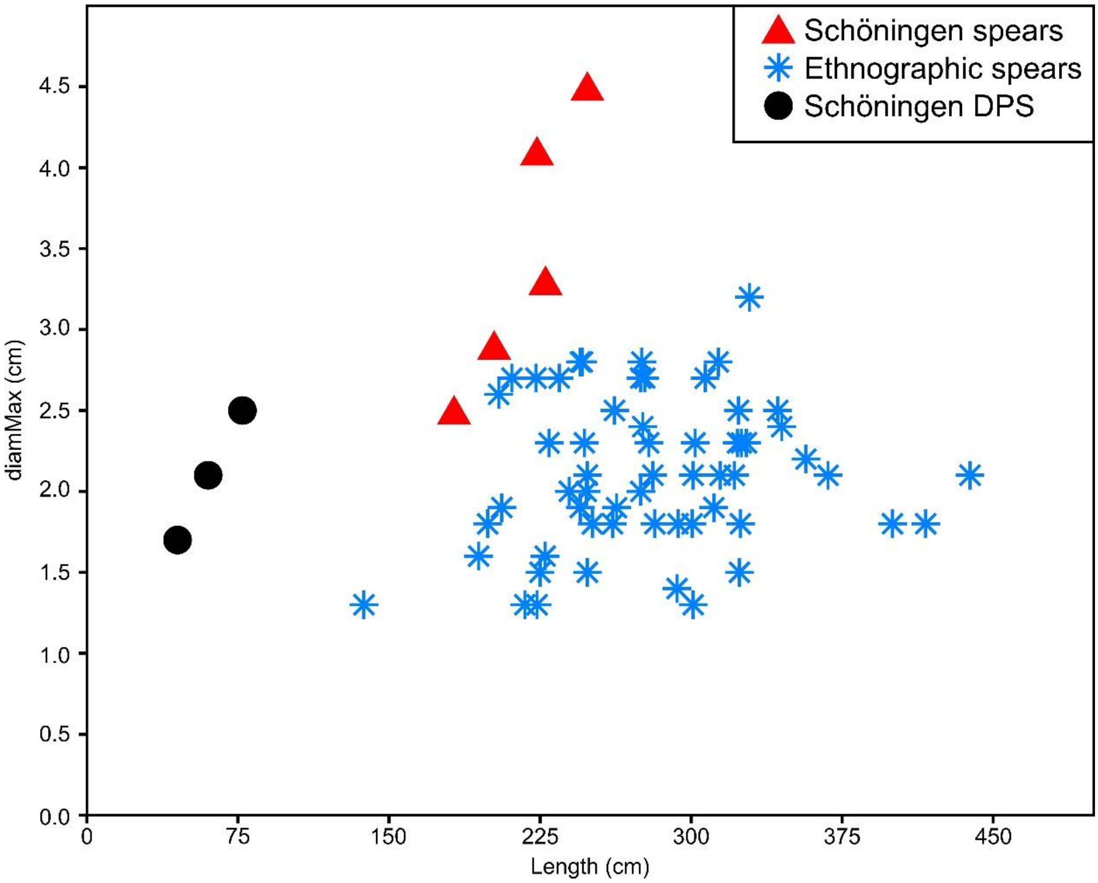

Fig. S16. Morphometric comparison of Schöningen's spears and double-pointed sticks (DPSs) with ethnographic spears. Chart: Annemieke Milks, Reading.

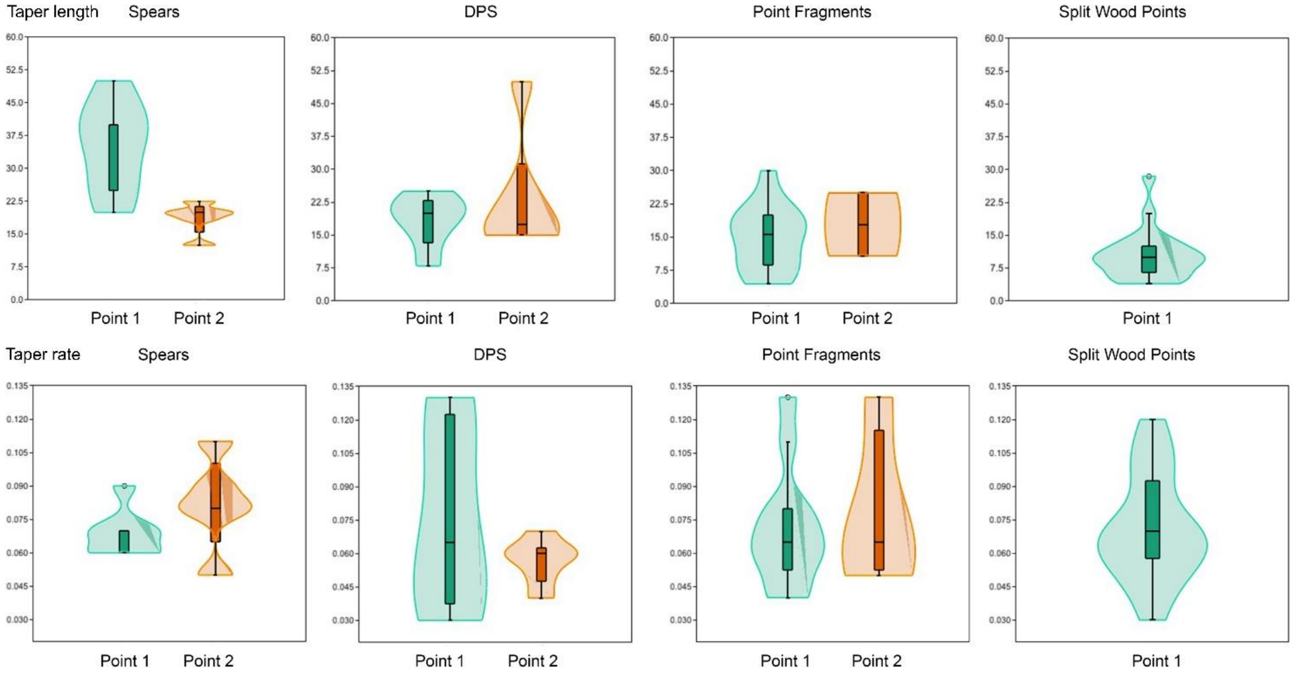

Fig. S17. Violin plots of the various pointed tool classes. Chart: Annemieke Milks, Reading.

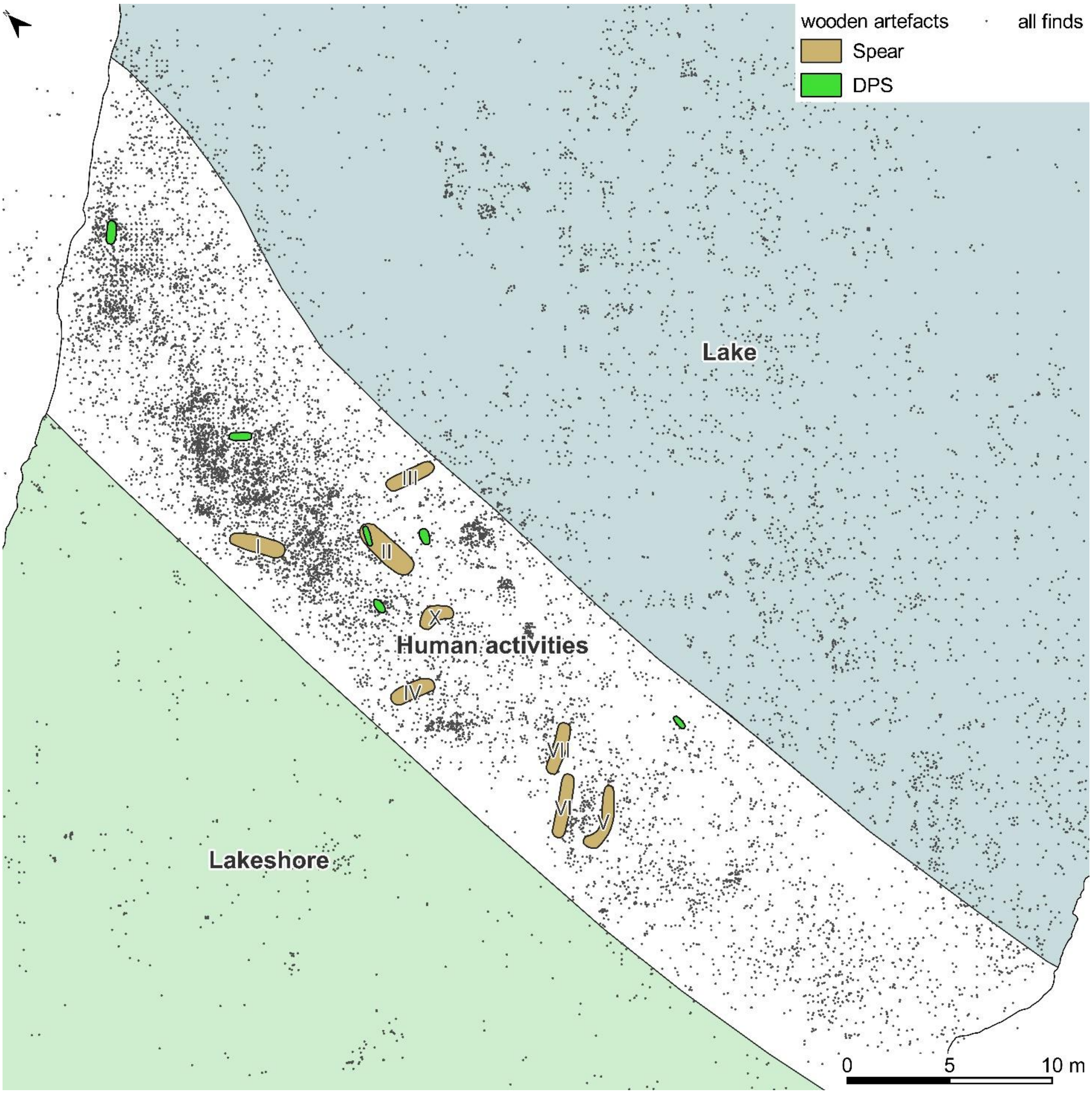

Fig. S18. Spatial distribution of all finds along the former lakeshore at Schöningen 13 II-4. Most finds are concentrated in the zone of human activities. DPS = double-pointed stick. Map: Dirk Leder, NLD.

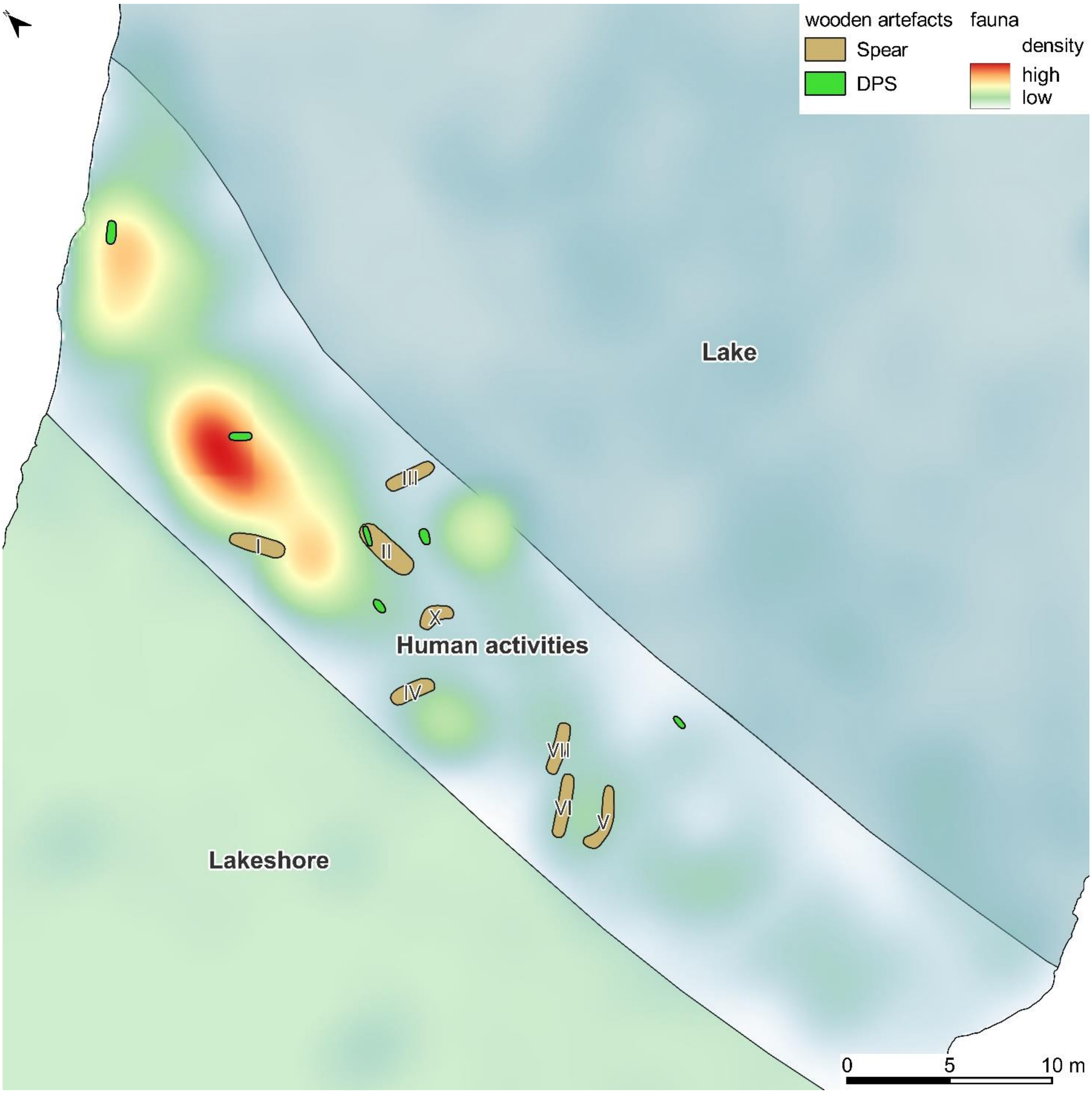

Fig. S19. Spatial distribution of faunal remains and major wooden artefacts along the former lakeshore at Schöningen 13 II-4. Finds are concentrated in the northern part of the human activities zone. Density mapping was done using the Kernel density function. DPS = double-pointed stick. Map: Dirk Leder, NLD.

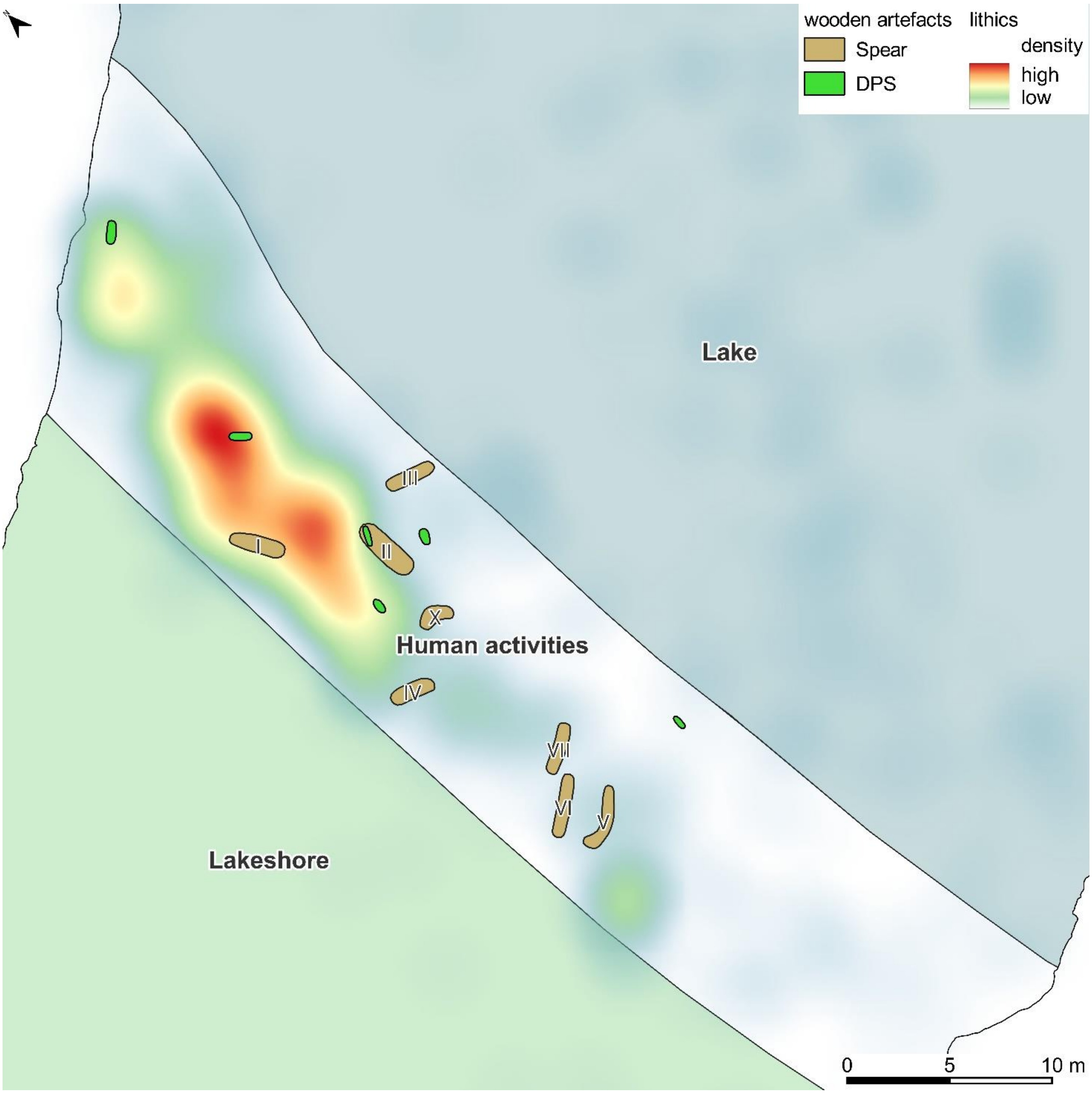

Fig. S20. Spatial distribution of lithic finds and major wooden artefacts along the former lakeshore at Schöningen 13 II-4. Finds are concentrated in the northern part of the human activities zone. Density mapping was done using the Kernel density function. DPS = double-pointed stick. Map: Dirk Leder, NLD.

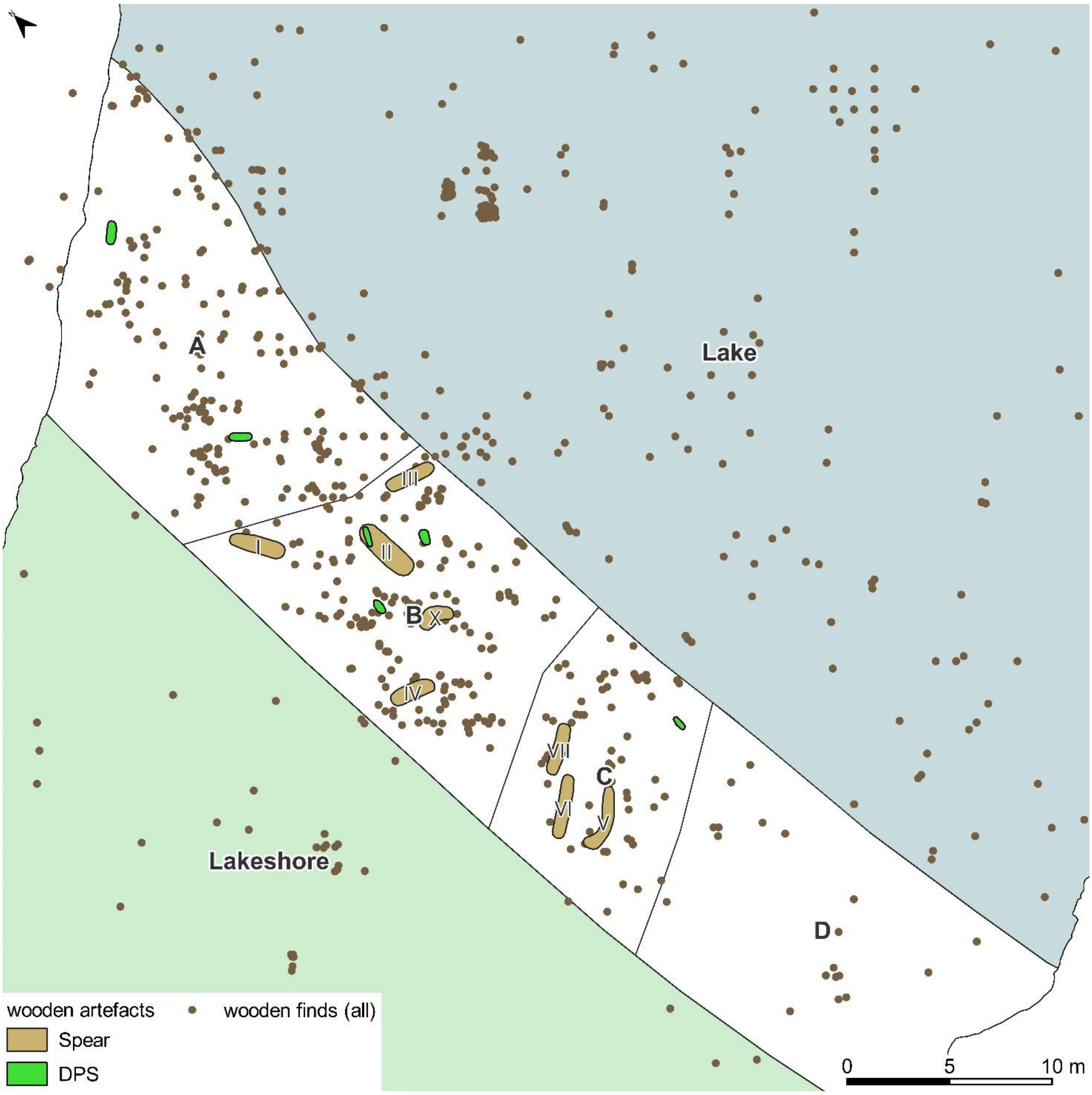

Fig. S21. Spatial distribution of all wood finds and major wooden artefacts along the former lakeshore at Schöningen 13 II-4. Finds include artefacts as well as natural woods. Most finds are concentrated in the zone of human activities and the former lake. DPS = double-pointed stick. Map: Dirk Leder, NLD.

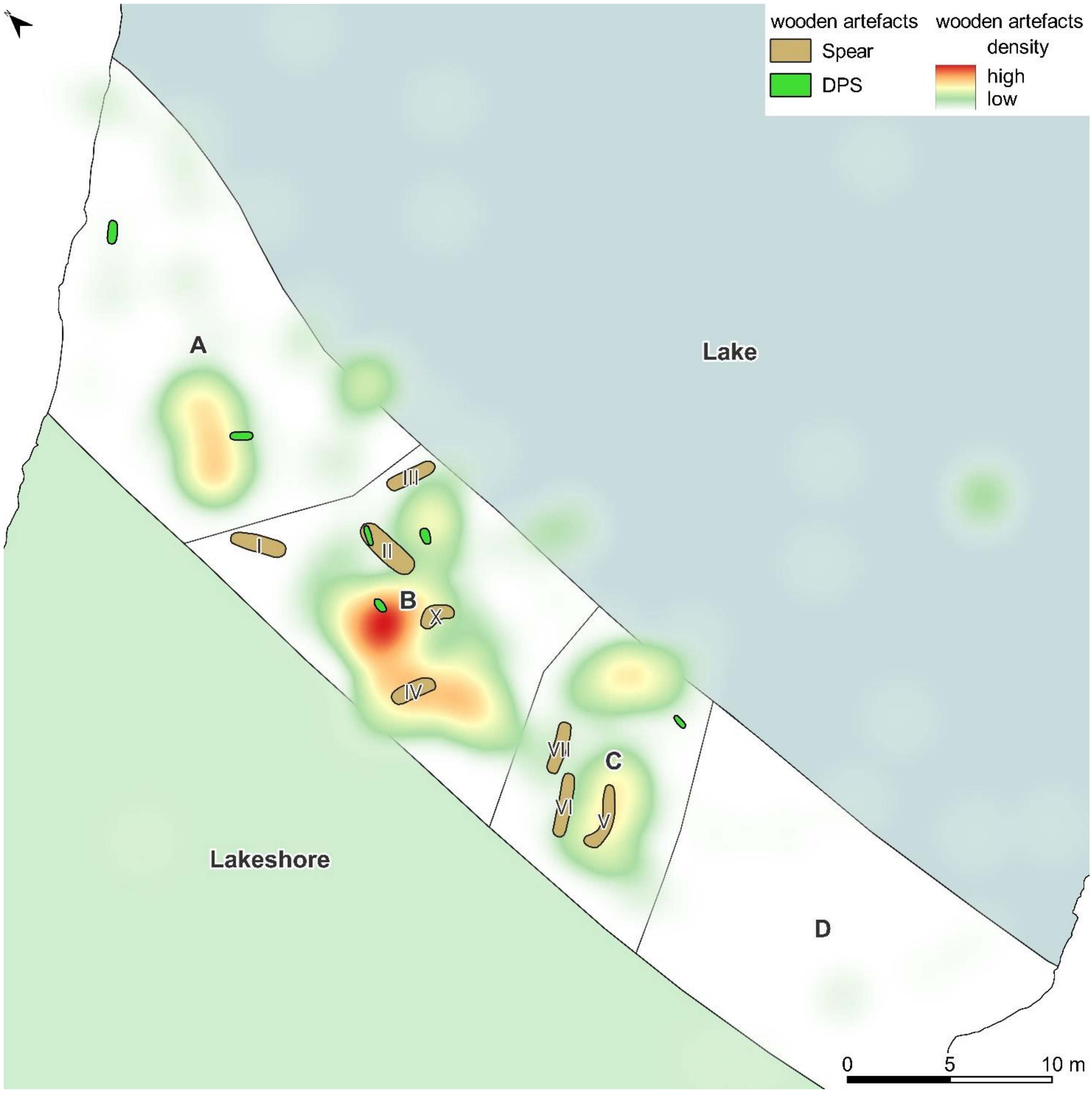

Fig. S22. Spatial distribution of wooden artefacts and major wooden artefacts along the former lakeshore at Schöningen 13 II-4. Finds are concentrated in the central part of the human activities zone and areas north and south of it. Border of sectors A-D are based on artefact density. Density mapping was done using the heatmap function. DPS = double-pointed stick. Map: Dirk Leder, NLD.

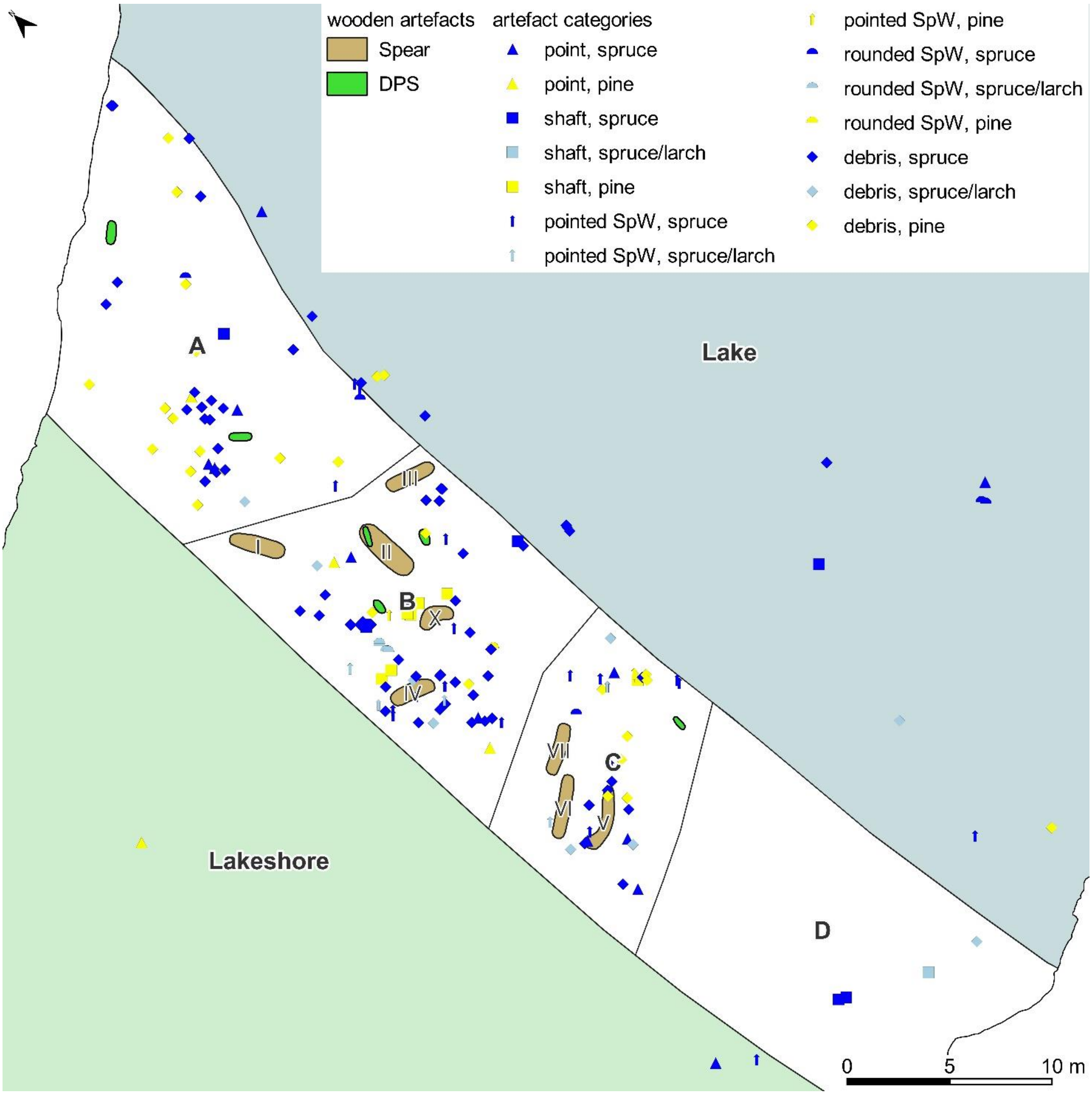

Fig. S23. Spatial distribution of wooden artefacts along the former lakeshore at Schöningen 13 II-4. Most finds are concentrated in the zone of human activities. Sectors A-D according to differing artefact compositions (Si-Text). DPS = double-pointed stick. Spw = split wood. Map: Dirk Leder, NLD.

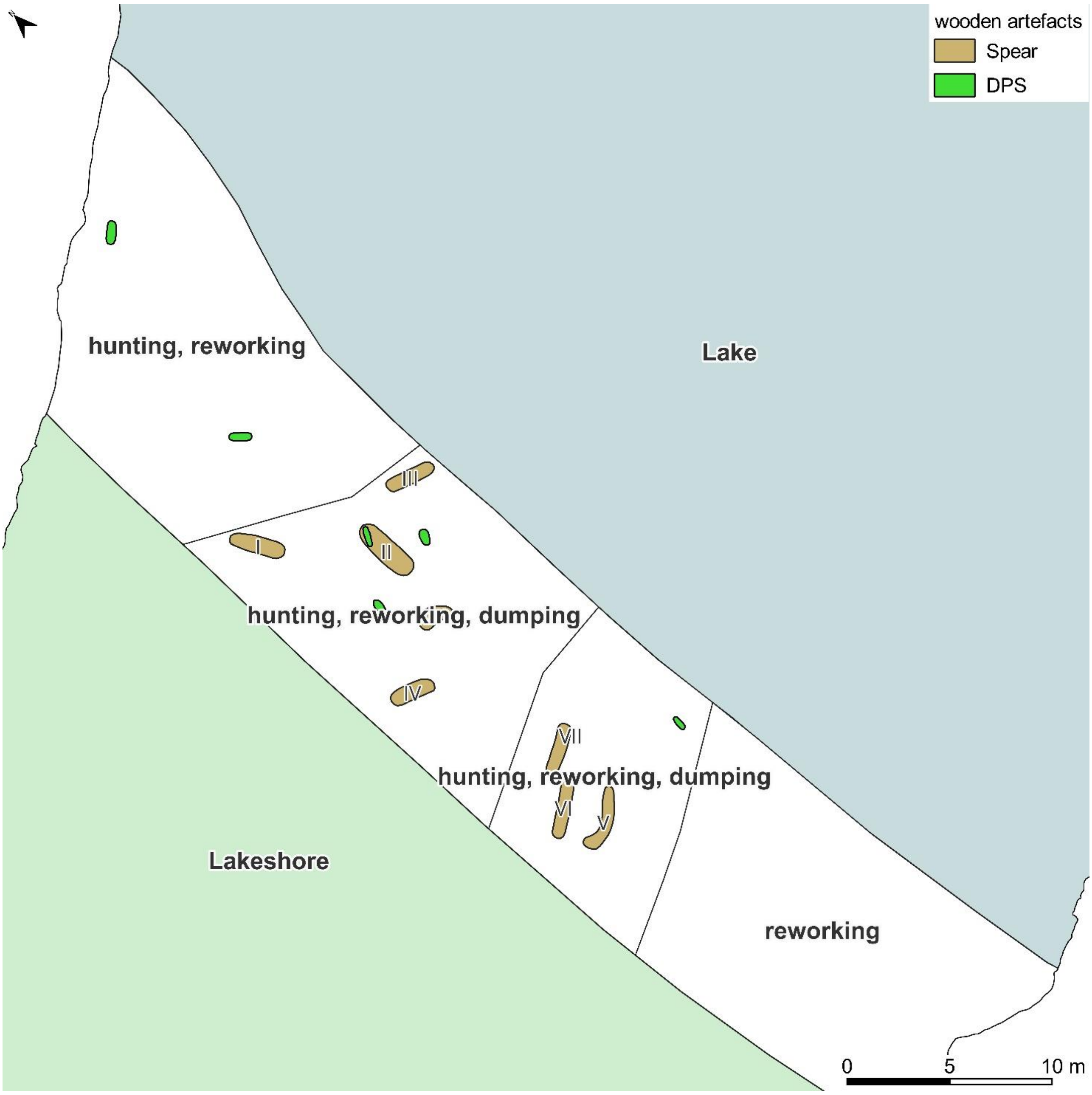

Fig. S24. Reconstructed activity zones (sectors A-D) based on wooden artefact compositions (Si-Text) at the former lakeshore at Schöningen 13 II-4. DPS = double-pointed stick. Map: Dirk Leder, NLD.

SI-Tables

Table S1. Wood assemblage (natural & worked) from Schöningen 13 II-4.

**Wood species**	single finds	bulk finds	total	%
deciduous	89	152	241	32.4
Alnus	*0*	*1*	*1*	0.1
Alnus/Betula	*0*	*1*	*1*	0.1
Betula	*6*	*9*	*15*	2.0
Juniperus	*1*	*2*	*3*	0.4
Populus	*3*	*2*	*5*	0.7
Populus/Salix	*2*	*75*	*77*	10.3
Salix	*76*	*62*	*138*	18.5
Quercus	*1*	*0*	*1*	0.1
coniferous	213	35	248	33.3
Abies alba	*1*	*1*	*2*	0.3
Larix	*3*	*0*	*3*	0.4
Larix/Picea	*15*	*3*	*18*	2.4
Picea	*141*	*18*	*159*	21.4
Pinus	*53*	*13*	*66*	8.9
indet.	63	182	255	34.3
total	365	369	744	100.0

Table S2. Refitted IDs.

**ID**	**category**	**spruce**	**spruce /larch**	**pine**	**tool**	**layer**	**refit distance (m)**
7344, 7362	1	x			DPS	4b/4c	0.3
11383, 12886	1	x			shaft fgm.	4b/4c	0.16
7107, 7108	1	x			shaft fgm.	4b/4c	0.04
7547, 7548	1			x	shaft fgm.?	4c	0.1
7288, 7289, 7304, 7305	1	x	x		round-ended SpW	4b/4c	0.6
7724, 7726, 7727	1			x	round-ended SpW	4c	0.15
7501, 7507	1	x			pointed split wood	4b	0.2
9920, 9922	1			x	pointed split wood	4c	0.02
4690, 5138, 5721, 5748, 5739	1	x			Spear I	4b/4c	0.03
7170_7172_7173	1	x			Spear II	4b/4c	0.3
7611, 7756, 17153	1	x			Spear III	4b/4c	0.03
7691, 7693, 7702, 18235	1	x			Spear IV	4b/4c	0.25
9613, 9668, 9669, 9670	1	x			Spear V	4b/4c	1.5
9202, 9277, 9311	1	x			Spear VI	4b	0.02
15678, 18221	1	x			Spear VII	4b?	0.01
7656, 7852, 7854	1			x	Spear X	4b/4c	0.3

The 16 refitted artefacts consist of 46 IDs. DPS= double-pointed stick. SpW = split wood.

Table S3. Artefact categories by wood species.

category	spruce	spruce/larch	pine	others*	indet.	total	%	sp/l vs pine ratio
1	43	6	13	0	0	62	8.7	*3.8:1*
2	12	3	4	0	0	19	2.7	*3.8:1*
3	69	9	28	0	0	106	14.8	*2.8:1*
4	15	0	14	243	255	527	73.8	*1.1:1*
total	139	18	59	243	255	714	100.0	
% total	19.5	2.5	8.3	34.0	35.7	100.0		

46 IDs refit to 16 artefacts affecting the total number of artefacts (n= 30, see Tables S1, S2). *others include all deciduous species and *Abies alba. Categories 1-4 are defined in the methods sections.*

Table S4. Wood IDs from siltation cycle 4 listed by sub-unit.

Sub-unit	category 1			category 2			category 3			category 4			
	spruce	spruce /larch	pine	spruce	spruce /larch	pine	spruce	spruce /larch	pine	spruce /larch	pine	total	%
4a							1		1			2	0.9
4a/b	2		1					1	3			7	3.2
4b	17	7	3	8	2	3	52	5	18	6	10	131	60.6
4b/c	19	2	4	4			9	2	3	1		44	20.4
4c	3		4	2		1	4		2		2	18	8.3
indet	1			2			2		1	6	2	14	6.5
total	42	9	12	16	2	4	68	8	28	13	14	216	100
%	19.4	4.2	5.6	7.4	0.9	1.9	31.5	3.7	13.0	6.0	6.5	100	

4a organic mud/detritus, 4b organic mud/detritus, 4b/c calcareous organic mud, 4c calcareous mud (lake) after Urban et al. 2023.

Table S5. Wooden artefacts (categories 1-3) by wood species.

artefact	spruce	spruce/larch	pine	total	%	sp/l vs pine ratio
spear	7		1	8	4.3	7:1
double-pointed stick	4	1	1	6	3.2	5:1
point fragment	13		4	17	9.0	3.3:1
shaft fragment*	6		7	13	6.9	0.9:1
pointed split wood	17	5	2	24	12.8	11:1
round ended split wood*	9	1	1	11	5.9	10:1
other fragments	3	2	4	9	4.8	2.3:1
splinters/debris	67	9	24	100	53.2	3.2:1
total	126	18	44	188	100.0	3.3:1

*Contains a shaft fragment (ID 11383_12886) worked into a round-ended split wood (SI-Fig.12).

Table S6. Spear data.

Spear	category	wood	length complete	length (cm)	diamMax (cm)	LMD %	taper-FP	taper-BP	taper length (FP)	taper length (BP)	BP pith location	annual rings	frost bite	summer draught	Phase 0	Phase 1	reworked (FP)	LC	shaft break	discoloration	SD	drying	delam	trampling	ED
I	1	spruce	x	223,5	4,1	31,5	0,06	0,08	50,0	18,5	centre	53	x		CT, IB	ARS, CM, ScM, SuF, TF		front/back	x	front/back	x			x	Cr, SD
II	1	spruce		227,7	3,3	35,1	0,06		40,0			55	x		Ca, CT, IB, FTI	ARS, CM, ScM, SuF, TF			x	front	x	x		x	Cr, SD
III	1	spruce	x	182,3	2,5	20,8	0,07	0,08	25,0	12,5	centre	33	x		CT, IB	ARS, CM, ScM, TF		back		front/back	x			x	BS, Cr
IV	1	pine		118,2	3,2	17,8		0,11		20,0	centre	18			CT	ARS, CM, ScM, SuF		back	x	back	x	x		x	Cr, Str
V	1	spruce	x	202,2	2,9	18,9	0,09	0,05	20,0	22,5	centre	49	x		IB	ARS, CM, ScM, SuF, TF	AS, SpN	front/back	x	front/back	x			x	Co, Cr, Str
VI	1	spruce		248,5	4,5	40,8	0,06	0,09	40,0	20,0	centre	57			CT, kink	ARS, CM, ScM, SuF, TF				front/back	x		x	x	BS, Cr, Str
VII	1	spruce		202,8	3,5	32,1	0,07		40,0			31		x	Ca, FTI	ARS, CM, ScM, SuF, TF		front	x	front	x	x	x		BS, Str
X	1	spruce		137,2	2,3	21,9	0,07		30,0			60				ARS, CM, ScM, SuF, TF	AS, SpN	front	x	?	x			x	Cr, Str
mean				192,8	3,2	27,4	0,07	0,08	35,0	18,7		44,5													
median				202,5	3,2	26,7	0,07	0,08	40,0	20,0		51,0													

Abbreviations: diamMax= maximum diameter; LMD%= location of diamMax measured as distance from the front point and given in percentage of the total length; FP= front point; BP= back point; LC= longitudinal crushing; SD= surface damage; delam= delaminated surface; ED= excavation and conservation damage; all other code after Milks et al. 2022.

Table S7. Double-pointed stick (DPS) data.

ID	category	wood	blank	complete	length	diamMax (cm)	LMD %	taper P1	taper length	taper P2	taper length	Phase 0	Phase 1	reworked	use	LC	discoloration	SD	drying	delam	trampling	ED
1779	1	spruce	branch	x	77.2	2.5	60.9	0.12	22.2	0.04	50.0	CW, Kn	ARS, CM, ScM, SuF, TF		UP?		x	x			x	BS, Cr
7172	1	spruce	trunk		60.2	2.1	71.0	0.04	20.0	0.07	15.0	Kn, Wh	ARS, CM, ScM, SuF		SB?		x	x	x			
10167	1	spruce/larch	split wood	x	45.0	1.7	66.7	0.08	15.0	0.06	15.0		DS	AS, SpW, SpM	UP?	x		x				BS
15677	1	spruce	trunk		87.7	3.0	51.3	0.13	8.0	0.06	25.0	Kn	ARS, CM, ScM, TF	AS, Cha, SpM, SuF	UP, SpN	x	x	x	x	x	x	BS, Cr
7344_7362	1	spruce	trunk		65.3	2.4	50.5	0.03	20.0	0.06	20.0	Kn	ARS, CM, ScM, TF	AS, SpN	BFr; SB	?					x	BS, Co
7724_7726_7727	1	pine	split wood		50.0	1.9	50.0	0.05	25.0	0.05	15.0	Kn (1x)	AS, ARS, CM, DS	SpW, SpM, SuF	UP; SB	x	x					BS
28108*	1	spruce	branch/trunk		64.5	2.9																
mean					64.3	2.4	58.4	0.08	18.4	0.05	23.3											
median					64.5	2.4	56.1	0.06	20.0	0.06	17.5											

*Conard et al. 2020. Abbreviations: diamMax= maximum diameter; LMD%= location of diamMax measured as distance from the front point and given in percentage of the total length; P1= point 1; P2= point 2; LC= longitudinal crushing; SD= surface damage; delam= delaminated surface; ED= excavation and conservation damage; all other code after Milks et al. 2022.

Table S8. Point fragment data.

ID	category	wood	trunk	front/back	tip present	length (cm)	diamMax (cm)	pith location	Taper	Taper length	Phase 0	Phase 1	reworked	LC	tip break	shaft break	discoloration	SD	drying	delam	trampling	notch	ED
3635	1	spruce	?	front/back	x	3.1	1.1				Kn	ARS, SM, TF		x									
3858	1	pine	x	front?		16.2	2.4		0.04	16.2	Kn, Wh	ARS, CM, St			x			x					
4236	1	spruce	x	front		37.5	2.8		0.06	20.0	Kn, Wh	ARS, CaM, CM, ScM, TF	x			x		x		x			
4934	2	spruce	?	front		8.9	1.4		0.08	8.9	Kn	AS, CM, ScM, SuF			x	x		x	x				
5600	1	spruce	?	front	x	8.3	1.1	offset	0.13	4.5	Kn	ARS, CaM, SF		?				x					BS
6923	1	pine	?	front		15.7	1.9		0.05	15.0		ARS, CaM, ScM, TF			x								BS
6935	1	spruce	?	front/back	x	10.7	1.0		0.07	10.7		ARS, ScM, SuF		x		x							
7567	1	spruce	x	back?	x	29.3	2.1		0.05	25.0	Kn	ARS, CaM, SM, TF		x		x		x	x	x	x		
8674	2	pine	?	back?	x	10.8	1.4	centre	0.06	10.80	Kn	CM, SM, SF		?		?							
8689	1	spruce	?	front	x	8.6	1.1	offset	0.04	8.60	Kn	ARS, CM, TF		x		x		x				x	
9388	1	spruce	?	front	x	18.2	1.9	offset	0.08	18.2	Kn	ARS, CaM, SM, TF		?		x		x	x	x			BS
9772	1	spruce	x	front		40.5	2.8		0.06	20.0	Kn, Wh	ARS, CM, ScM				?		x	x	x	x		
9788	1	spruce	?	front/back		8.2	1.3		0.11	5.0	Kn	ARS, ScM, SM			x								BS, Str
12074*	1	spruce	?	front	x	25.0	1.8	offset	0.06	20.0	Kn	ARS, SM, SuF				x	x	x	x	x			BS, CM
14852**	1	spruce	x	front	x	56.7	2.8	offset	0.07	30.0	Kn, WH	ARS, CM, ScM, SuF, TF	AS, SpN	x			?	x	x	x			BS, Cr
15146	1	spruce	?	back	x	11.9	1.9		0.13	10.0	Kn	ARS, CM, TF		x		x		x					
18342	3	pine	x	back	x	34.0	1.9	centre	0.07	10.0	Kn, Wh	BeC, SpN		?		?		x	x	x			
mean						20.2	1.8		0.07	14.6													
median						15.7	1.9		0.07	12.9													

* former Spear IX

** former Spear VIII, reworked point

Abbreviations: front/back= front point or back point; diamMax= maximum diameter; LC= longitudinal crushing; SD= surface damage; delam= delaminated surface; ED= excavation and conservation damage; all other code after Milks et al. 2022.

Table S9. Shaft fragment data.

ID	category	wood	trunk	debarked	split	length (cm)	widthMax (cm)	ThMax (cm)	diam/Max (cm)	pith location	Phase 0	Phase 1	reworked	shaft break	discoloration	SD	drying	delam	trampling	ED
4553	1	spruce	?	x	probably	16.9	2.3	0.8		outside	Kn	ScM, SuF, TAR	split							
7296	1	pine	Tr	x	no	33.8	3.1	2.2	2.7	centre	CT, Kn, Wh, Se	CM, SuF, TF	-	x					x	sampling, BS, Co, Str
7512	1	pine	Tr	x	no	15.4	2.7	1.7	2.2	centre	Kn, Wh	ARS, ScM, TF	-	x		x	x		x	
7562	3	pine	Tr	x	along pith	12.0	2.3	1.0		centre	FTI, Kn	CM?	split?			x	x			BS
7705	3	pine	?	x	along pith	12.0	2.3	1.0		centre	Kn	CM?	split?	?		x	x	x		
7880	3	pine	?	x	along pith	18.0	2.3	1.6		centre	Kn	CM?	split?			x		x		
8988	2	spruce	Tr	x	along pith	19.5	2.2	1.4	1.8	centre	Kn, KH, Se	CM, Str	split, BeC	?		x		x		
9921	1	pine	Tr	x	parallel to pith	19.3	3.1	1.3		outside	Ca, Kn	RAR, ScM, SuF	split	?						
11125	1	spruce	Tr	x	parallel to pith	19.8	3.1	1.8		outside	Kn, KH, Se	CM, Str, ToS	split, splinter extraction, edge AS	?						
11979	3	spruce/larch	?	DS, Ca	no	19.0	2.5	2.5	2.5	centre	CT, Kn	CM?	-	?	Cha?			x		del
11383_12886*	1	spruce	Tr	Ca, DS	no	82.8	3.8	2.5	3.2	centre	Ca, CT, Kn, Se	ARS, CM, ScM, TF	splinter extraction, round end			x	x	x	x	
7107_7108**	1	spruce	?	Ca, DS	no	63.8	2.4	1.6	2.0	centre	Ca, KH, Kn, kink	ChM?, CM, Str, TM	SpM?	x		x		x		
7547_7548	1	pine	Tr	DS, Ca	tangential	32.3	3.5	1.8		centre, inside	CT, Kn, Wh	CM, ScM, SuF, TF	split				x		x	
mean						28.0	2.7	1.6	2.4											
median						19.3	2.5	1.6	2.4											

*shaft reworked into round-ended split wood

**natural kink, spear fragment

Abbreviations: widthMax= maximum width; ThMax= maximum thickness; diamMax= maximum diameter, calculated whenever complete circumference was preserved; SD= surface damage; delam= delaminated surface; ED= excavation and conservation damage; all other code after Milks et al. 2022.

Table S10. Classifications of category 1-3 roundwood artefacts used in calculating the MNI of hunting weapons.

artefact	spruce /larch*	pine	criteria	classification	tool
Spear I	1		>1.1 m; one front point; one back point	spear	spear
Spear II	1		>1.1 m; one front point	spear	spear
Spear III	1		>1.1 m; one front point; one back point	spear	spear
Spear IV		1	>1.1 m; one back point	spear	spear - pine
Spear V	1		>1.1 m; one front point; one back point	spear	spear
Spear VI	1		>1.1 m; one front point	spear	spear
Spear VII	1		>1.1 m; one front point	spear	spear
Spear X	1		>1.1 m; one front point	spear	spear
ID 1779	1		<1.1 m; two ends taper; two points	DPS	DPS
ID 3635	1		<1.1 m; one end tapers; pith unclear	front/back point	spear/DPS
ID 3858		1	<1.1 m; one end tapers; pith offset; pine	front point	spear - pine
ID 4236	1		<1.1 m; one end tapers; pith offset	front point	spear/DPS
ID 4553	1		<1.1 m; no taper	shaft	spear/DPS
ID 4934	1		<1.1 m; one end tapers; pith offset	front point	spear/DPS
ID 5600	1		<1.1 m; one end tapers; pith offset	front point	spear/DPS
ID 6923		1	<1.1 m; one end tapers; pith offset; pine	front point	spear - pine
ID 6935	1		<1.1 m; one end tapers; pith unclear	front/back point	spear/DPS
ID 7107_7108	1		<1.1 m; no taper; cambium	shaft	spear
ID 7172	1		<1.1 m; two ends taper; one point	DPS	DPS
ID 7296		1	<1.1 m; no taper; pine	shaft	spear - pine
ID 7344_7362	1		<1.1 m; two ends taper; no points	DPS	DPS
ID 7512		1	<1.1 m; no taper; pine	shaft	spear - pine
ID 7547_7548		1	<1.1 m; no taper; pine	shaft	spear - pine
ID 7562		1	<1.1 m; no taper; pine	shaft	spear - pine
ID 7567	1		<1.1 m; one end tapers; pith centre	back point	spear
ID 7705		1	<1.1 m; no taper; pine	shaft	spear - pine
ID 7880		1	<1.1 m; no taper; pine	shaft	spear - pine
ID 8674		1	<1.1 m; one end tapers; pith centre; pine	back point	spear - pine
ID 8689	1		<1.1 m; one end tapers; pith offset	front point	spear/DPS
ID 8988	1		<1.1 m; no taper	shaft	spear/DPS
ID 9388	1		<1.1 m; one end tapers; pith offset	front point	spear/DPS
ID 9772	1		<1.1 m; one end tapers; pith offset	front point	spear/DPS
ID 9788	1		<1.1 m; one end tapers; pith unclear	front/back point	spear/DPS
ID 9921		1	<1.1 m; no taper; pine	shaft	spear; pine
ID 11125	1		<1.1 m; no taper	shaft	spear/DPS
ID 11383_12886	1		<1.1 m; no taper; cambium	shaft	spear
ID 11979	1		<1.1 m; no taper	shaft	spear/DPS
ID 12074	1		<1.1 m; one end tapers; pith offset	front point	spear/DPS
ID 14852	1		<1.1 m; one end tapers; pith offset	front point	spear/DPS
ID 15146	1		<1.1 m; one end tapers; pith centre	back point	spear
ID 15677	1		<1.1 m; two ends taper; no points	DPS	DPS
ID 18342		1	<1.1 m; one end tapers; pith centre; pine	back point	spear - pine
**total**	**30**	**12**			

The two double-pointed sticks (DPS) on split woods are not included here. *All items are made from spruce apart from ID 11979 that is made from either spruce or larch.

Table S11. MNI of spruce/larch spears assuming all spruce/larch point fragments belong to spears (SpearMAX).

artefact	front point	back point	front/back?
spear, complete	3	3	
spear, fragmented	4		
point fragment	8	2	3
total	15	5	3
**MNI = 15**			

Table S12. MNI of spruce/larch spears assuming relevant spruce/larch point fragments belong to double-pointed sticks (DPSMax).

artefact	front point	back point	front/back?
spear, complete	3	3	
spear, fragmented	4		
point fragment		2	
total	7	5	0
**MNI = 7**			

Table S13. MNI of spruce/larch double-pointed sticks (DPSs) assuming relevant spruce/larch point fragments belong to DPSs (DPSMax).

artefact	n=	points missing	point fgms. remaining	reconstructed DPS
DPS, complete	1	0	0	1
DPS, fragmented	3	5	0	3
point fragment*	11	-	6	3
total	-	-	-	7
**MNI = 7**				

*8 front points, 3 front or back points

Table S14. MNI of spruce/larch and pine shaft fragments.

shaft frgms.	total length (cm)	spruce spear MNI	spear/DPS MNI	pine spear MNI
spruce/larch with cambium	146.6	1		
spruce/larch debarked	75.2		1	
pine	142.8			1
**total**	364.6	1	1	1

DPS= double-pointed stick.

Table S15. MNI of pine spears considering point fragment made from pinewood.

artefact	front point	back point	front/back?
spear, fragmented		1	
point fragmnent	2	2	
total	2	3	0
**MNI = 3**			

Table S16. MNI of hunting weapons considering SpearMax and DPSMax scenarios.

artefact	SpearMax	DPSMax
spear, spruce/larch	15	7
spear, pine	3	3
DPS, roundwood	4	7
DPS, SpW	2	2
total	24	19

DPS= double-pointed stick. SpW = split wood.

Table S17. Pointed split wood data.

ID	category	Wood	trunk	debarked	split	length (cm)	width Max (cm)	ThMax (cm)	pith location	tip present	Tip area mm^2	Taper width	Taper thickness	taper diam	taper length	Phase 0	Phase 1	reworked	Abrasion	LC	discoloration	SD	drying	delam	notch	trampling	FUI/FTI	ED
6826	3	spruce/larch		x	possibly	17.5	1.2	0.4	absent	x	12	0.06	0.01	0.04	12.5			SpW/SP, SpN	tip									BS
7031	1	spruce			yes	20.3	1.4	0.7	absent	x	21	0.04	0.03	0.03	15.0			SpW, AS, St	surface			x						BS
7243	1	spruce	x	x	yes	28.5	2.7	1.2	centre	x	40	0.10	0.02	0.06	28.5	Kn	ScM, ARS	SpW, AS, SpM, ScM, St	surface	x								
7245	1	spruce			yes	10.5	1.7	0.9	absent	x	32	0.09	0.13	0.11	10.0			SpW, AS, SuF	surface									
7320	3	pine			possibly	9.5	1.5	0.5	absent	x	16	0.10	0.05	0.07	9.0			SpW/SP	tip	x					x			
7503	3	spruce/larch			possibly	15.7	1.5	0.6	absent									SpW/SP, AS, SuF, SpN	surface									
7832	2	spruce			yes	15.9	1.4	0.7	absent	x	18	0.09	0.03	0.06	12.5			SpW, AS, St	tip	x		x						
7878	1	spruce		x	yes	15.8	1.1	0.5	absent	x	8	0.12	0.07	0.10	7.5	Kn	ScM, St, ARS	SpW, AS, SuF	tip			x						
7898	3	spruce			possibly	6.5	0.6	0.4	absent									SpW/SP	surface									
8070	1	spruce/larch		x	yes	13.5	1.8	0.7	absent	x	28	0.10	0.03	0.07	12.0		ScM, ARS	SpW, AS, ScM, SuF	surface			x						
8097	3	spruce			possibly	4.6	1.0	0.3	absent	x	18	0.13	0.00	0.07	4.0			SpW/SP, AS	tip									
9109	2	spruce/larch			yes	11.8	1.2	0.7	absent	x	18	0.07	0.05	0.06	10.0			SpW, AS, SM, St	tip			x						BS
9368	3	spruce		x	possibly	11.4	1.1	0.6	absent	x								SpW/SP, SM, Str	surface	x					x			
9424	1	spruce			yes	15.5	1.3	0.7	absent									SpW, AS, SM, SuF	surface									sample
9775	2	spruce/larch			yes	5.9	1.4	0.6	absent	x	35	0.17	0.07	0.12	5.5			SpW, AS, St	surface		Cha							CM, Cr
9776	1	spruce			yes	8.5	1.5	0.5	absent	x	15	0.12	0.03	0.07	8.0			SpW, AS, SuF	surface		Cha							
10170	2	spruce		x	yes	36.0	1.9	1.0	absent								ScM, St	SpW, AS, SpM, St	surface									BS
10172	1	spruce		x	yes	13.3	1.2	0.7	absent	x	18	0.13	0.04	0.09	10.0		ScM, St	SpW, AS, CaM, SuF	tip			x				x		
12061	2	spruce			yes	17.2	1.4	0.8	absent	x	15	0.07	0.06	0.06	14.0			SpW, AS, CM, St	surface			x						
14903*	1	spruce	x	x	yes	34.7	2.6	1.2	centre	x	32	0.08	0.06	0.07	10.0	Kn, Wh	AS, SM, St	SpW, SpM, SuF, Po	tip				x	x		x	x	BS
18244	2	spruce		x	yes	8.1	1.0	0.5	absent								ScM, SM	SpW, AS, SpN	surface			x						
18304	2	spruce			yes	4.8	0.8	0.3	absent	x	12	0.10	0.00	0.05	4.5			SpW, AS, St, Str	surface									
7501_7507	1	spruce			yes	23.1	1.5	0.5	absent	x	21	0.16	0.04	0.10	5.0			SpW, AS, BeC, ScM. TF	surface	x								BS, CM

9920_9922**	1	pine	?	x	yes	29.6	1.9	1.9	offset	x	63	0.04	0.06	0.05	20.0	Kn	AS, SM, ToS, St	SpM, AS, CaM, Po	surface	x		x				x
mean						15.8	1.4	0.7			23.4	0.10	0.04	0.07	11.0											
median						14.5	1.4	0.7			18.0	0.10	0.04	0.07	10.0											

*14903, split roundwood with similar metrics as the point fragment (ID 14852) nearby; 2.7 cm wide and 1.2 cm thick.
**9920_9222, spear point re-worked by splitting; 2 cm wide and 2 cm thick
*,** both upper limit among pointed split woods
Abbreviations: widthMax= maximum width; ThMax= maximum thickness; tip area mm² calculated at 1 cm distance from the very tip; taper diam= taper diameter calculated from taper width and taper thickness; LC= longitudinal crushing; SD= surface damage; delam= delaminated surface; FUI/FTI= fungal infestation/feeding traces of insects; ED= excavation and conservation damage; all other code after Milks et al. 2022.

Table S18. Round-ended split wood data.

ID	wood	category	trunk	debarked	split	length (cm)	widthMax (cm)	ThMax (cm)	pith location	tool end shape	Tip area mm²	2nd taper	Phase 0	Phase 1	reworked	Use	LC	shaft break	discoloration	SD	drying	delam	trampling	ED
3198	spruce	1	x	x	x	56.1	3.3	2.0	absent	straight	102	x	Kn, 18+ rings	AS, ARS, ScM, SM	SpW, ScM, SuF, TF, SpM	SpN at tip, UP								
7244	spruce	1	x	x	x	23.5	2.1	1.3	along pith	round	100		Kn	AS, ScM, Str, TF	SpW, AS	SpN at tip, UP					x		x	BeC
7287	spruce	1	x	x	x	18.8	2.0	0.7	along pith	round	65		Kn	AS, ScM, SM, St	SpW, SpM, ScM, AS	UP				x		x		
8411	pine	2		x	x	25.5	1.7	0.7	absent	round	48	x	Kn	AS, ARS	SpW, AS, SuF, St	SpN at tip, UP	x				x			
9349	spruce	1		x	x	19.0	2.5	0.8	absent	straight	140		Kn	AS, ScM, St	SpW, SpM, ARS	Str, UP		BFr	orange flecks	x		x		
9605	spruce	1			x	14.5	1.9	0.7	absent	round + straight	65				SpW, SpM, SuF, AS	SpN at tip, UP	x	cut?		x				
12071	spruce	1		x	x	30.0	2.6	1.1	absent	round	176	x	KH	AS, St	SpW, SpM, AS	SpN, UP, Re			orange at tip		x	x		
12072	spruce	2	x	x	x	27.0	2.7	2.1	absent	round	105	x	Kn	AS, ARS	SpW, SpM, AS, St	SpN at tip, Str, UP			orange flecks	x	x	x		
12073	spruce	2			x	29.1	2.9	0.7	absent	round	60				SpW, SpM, SuF, TF, AS	UP			orange flecks		x	x		
7288, 7289, 7304, 7305	spruce/larch	1	?		x	54.6	2.7	1.7	absent	round	144				SpW, SpM. CM, SM, AS	SpN at tip, UP		x		x	x	x		BS
11383, 12886	spruce	1	x	x	x	82.8	3.8	2.5	along pith	straight ?	150		Kn	AS, ARS, CM, ScM, TF	SpW, SpM. ARS, AS	SpN, UP				x	x	x	x	sample, BS, Del
mean						34.6	2.6	1.3			105.0													
median						27.0	2.6	1.1			102.0													

Abbreviations: widthMax= maximum width; ThMax= maximum thickness; tip area mm² calculated at 1 cm distance from the very tip; LC= longitudinal crushing; SD= surface damage; delam= delaminated surface; ED= excavation and conservation damage; all other code after Milks et al. 2022.

Table S19. Debris data.

ID	wood	category	type	description	splitting	debarked	no. finds	length	width	thickness	Phase 0	Phase 1	Phase 2	SB	discoloration	SD	drying	delam	trampling	ED
1049	pine	3	debris/splinter	split wood/splinter	possibly		4	3.5			Kn									
1105	spruce	3	debris/splinter	split wood/splinter	probably	x	1	5.4	1.7	0.4			UP?							Str
1621	spruce	3	debris/splinter	split wood/splinter	possibly	x	1	10.5	1.5	0.4	Kn									
1903	spruce	3	debris/splinter	split wood/splinter	possibly		1	4.5	1.2	0.3										
1904	spruce	3	debris/splinter	split wood/splinter	probably	x	1	13.7	1.9	0.7		TAR	AS, taper, BeC/BFr							
2407	pine	3	debris/splinter	split wood/splinter	probably	x	1	10.8	1.0	0.7										BS, Str, Co
2472	pine	1	debris/splinter	split wood/splinter	possibly	x	1	9.3	2.1	0.5	KH	ScM, TF, SuF								
2772	pine	3	debris/splinter	split wood/splinter	possibly	x	1	6.1	1.6	0.4										
3027	spruce	3	debris/splinter	split wood/splinter	probably	x	1	7.2	1.1	0.3					x					
3159	pine	3	debris/splinter	split wood/splinter	possibly	x	1	4.0	1.2	0.3					x					
3275	pine	3	debris/splinter	split wood/splinter	probably	x	1	8.3	1.9	0.5							x			
3335	spruce	3	debris/splinter	split wood/splinter	probably	x	1	7.2	1.5	1.4	Kn	ARS								Str
3437	pine	3	debris/splinter	split wood/splinter	probably	x	1	13.1	2.5	1.0						x	x	x		
3441	pine	3	debris/splinter	split wood/splinter	possibly	x	1	13.1	2.5	1.4							x	x		
3501	pine	3	debris/splinter	split wood/splinter	possibly	x	1	6.7	1.6	1.0										BS
3502	spruce	3	debris/splinter	split wood/splinter	probably	x	1	7.3	1.4	0.3										
3572	pine	3	debris/splinter	split wood/splinter	probably		1	9.1	1.8	1.0										
3804	spruce	1	debris/splinter	split wood/splinter	possibly	x	1	9.9	1.9	0.5		CM, TAR, ScM	SpM	x		x				
3829	spruce	3	debris/splinter	split wood/splinter	possibly	x	1	7.1	1.3	0.4	KH	ScM?						x		
3857	spruce	3	debris/splinter	split wood/splinter	possibly	x	1	6.3	1.5	0.9	Kn									BS
3957	pine	3	debris/splinter	split wood/splinter	probably		1	12.0	1.5	0.9										BS
4061	spruce	3	debris/splinter	split wood/splinter	probably		1	24.0	2.3	1.2										BS, Str
4229	spruce	3	debris/splinter	split wood/splinter	possibly		2	3.6	1.1	0.2										
4240	spruce	3	debris/splinter	split wood/splinter	possibly	x	1	11.4	2.5	1.2										
4277	pine	3	debris/splinter	split wood/splinter	possibly	x	2	4.1	2.1	0.6										BS
4301	spruce	3	Fragment	flake	no	x	1	11.5	1.2	0.7								x		
4474	spruce	3	debris/splinter	split wood/splinter	probably		1	9.1	1.3	0.3			taper, AS							BS
4767	spruce/larch	1	Fragment	branch	no	x	1	24.8	1.5	1.0	Kn	CM, TAR, Str	SpM, SpN	x						BS
5883	pine	3	debris/splinter	split wood/splinter	possibly		2	5.4	1.2	0.6										
6019	spruce	3	debris/splinter	split wood/splinter	possibly	x	3	5.8	0.9	0.4										BS

6344		1	debris/splinter	split wood/splinter	probably	x	1	6.4	1.2	0.4		TAR, ScM, SM							
6348	spruce	3	debris/splinter	split wood/splinter	possibly	x	1	4.5	1.2	0.3		ScM?							
6421	spruce	3	debris/splinter	split wood/splinter	possibly		2	2.1	1.0	0.1									
6433	spruce	3	debris/splinter	split wood/splinter	probably		1	5.5	0.9	0.2			UP?						BS
6458	spruce/larch	1	debris/splinter	split wood/splinter	yes	x	1	6.2	1.0	0.3		TAR, ScM			x	x	x		BS
6849	spruce	3	debris/splinter	split wood/splinter	probably		1	4.8	0.8	0.3			taper						
6850	spruce	3	debris/splinter	split wood/splinter	probably		1	8.7	1.6	0.3			taper						
7100	spruce	3	debris/splinter	split wood/splinter	probably		1	2.5	0.6	0.3									BS
7101	spruce	3	debris/splinter	split wood/splinter	probably	x	1	5.3	1.0	1.1	Kn								
7103	pine	3	debris/splinter	split wood/splinter	possibly		1	7.1	1.0	0.3							x		
7116	spruce	3	debris/splinter	split wood/splinter	possibly		1	5.1	0.9	0.3				x					
7246	spruce	2	debris/splinter	split wood/splinter	yes		1	10.5	1.5	1.0			AS, SuF	x		x	x	x	
7475	pine	1	debris/splinter	split wood/splinter	yes	x	1	8.7	1.2	0.5	KH	RAR, ScM	UP?			x			
7506	spruce	3	debris/splinter	split wood/splinter	probably		1	12.2	1.7	0.3			As/UP?						
7514	spruce	3	debris/splinter	split wood/splinter	probably	x	4	6.8											Co
7524	spruce	3	debris/splinter	split wood/splinter	probably		1	11.1	1.4	0.5					x				
7560	spruce	3	debris/splinter	split wood/splinter	probably		1	8.7	0.7	0.4					x				BS
7563	spruce/larch	3	debris/splinter	split wood/splinter	probably		1	10.5	2.3	0.7									BS
7672	spruce/larch	2	debris/splinter	split wood/splinter	yes		1	10.2	0.8	0.5	Kn		ScM, UP		x				BS
7725	pine	2	debris/splinter	split wood/splinter	possibly	x	1	11.0	2.1	0.7	KH	RAR, SM, SuF							BS
7755	spruce	3	debris/splinter	split wood/splinter	possibly	x	1	16.2	1.7	0.5					x				
7823	spruce	3	debris/splinter	split wood/splinter	probably		3	23.2	2.3	0.8									
7825	spruce/larch	3	debris/splinter	split wood/splinter	possibly		2	5.3	1.2	0.5			AS						
7850	spruce	3	debris/splinter	split wood/splinter	probably		1	7.3	1.1	0.4				x					BS
7882	spruce	3	debris/splinter	split wood/splinter	possibly		1	8.4	1.6	0.5									
7909	spruce	3	debris/splinter	split wood/splinter	possibly		1	9.5	1.2	0.6			taper, SpN						
7911	spruce	3	debris/splinter	split wood/splinter	possibly		1	7.3	1.5	0.7									
7918	spruce	3	debris/splinter	split wood/splinter	probably	x	1	4.0	1.1	0.7									
7942	pine	3	debris/splinter	split wood/splinter	possibly	x	1	7.7	2.1	0.9		ARS							BS
8074	spruce	3	debris/splinter	split wood/splinter	probably		1	8.0	1.6	0.3									
8075	spruce	3	debris/splinter	split wood/splinter	probably		1	16.5	0.9	0.8				x					BS
8086	spruce	3	debris/splinter	split wood/splinter	possibly	x	1	4.8	1.1	0.6		ARS							

8087	spruce	3	debris/splinter	split wood/splinter	possibly		1	3.0	0.6	0.3								
8091	spruce	3	debris/splinter	split wood/splinter	possibly		1	3.4	0.5	0.1								
8115	spruce	3	debris/splinter	split wood/splinter	probably		1	14.0	2.1	0.9								
8145	spruce	3	debris/splinter	split wood/splinter	probably		1	3.9	1.3	0.3								BS
8369	spruce	3	debris/splinter	split wood/splinter	possibly	x	1	6.2	1.0	0.4		ARS, St	taper, UP?					
8385	spruce	3	debris/splinter	split wood/splinter	probably		1	11.3	2.5	0.4								
8398	pine	1	Fragment	flake	no	x	1	6.5	2.2	0.3		CaM, ScM, SuF						
8415	spruce	3	debris/splinter	split wood/splinter	possibly		1	4.4	0.8	0.4								
8680	spruce	3	debris/splinter	split wood/splinter	possibly	x	1	5.5	0.5	0.2								BS
8693	spruce	3	debris/splinter	split wood/splinter	probably		1	6.2	0.6	0.3								BS
8700	spruce	3	debris/splinter	split wood/splinter	probably		1	5.0	0.6	0.4								BS
8989	spruce	2	debris/splinter	split wood/splinter	yes	x	1	13.3	1.2	0.4		ScM						
9155	spruce/larch	3	debris/splinter	split wood/splinter	probably	x	2	22.5	1.3	0.6								
9194	spruce	3	debris/splinter	split wood/splinter	probably		1	10.5	1.5	0.9								BS
9199	spruce	3	debris/splinter	split wood/splinter	probably		1	6.1	1.7	0.5								BS
9200	spruce	3	debris/splinter	split wood/splinter	probably		1	7.9	1.4	0.5			taper, UP, SpN					BS
9391	spruce	3	debris/splinter	split wood/splinter	probably		3	20.9	1.2	0.4								
9515	spruce	3	debris/splinter	split wood/splinter	probably		1	3.4	1.2	0.4								
9620	spruce/larch	3	debris/splinter	split wood/splinter	probably		1	8.5	1.4	0.5								BS
9675	pine	3	debris/splinter	split wood/splinter	possibly	x	1	5.8	1.5	0.6								
9724	spruce	3	debris/splinter	split wood/splinter	probably		1	8.0	0.7	0.4			taper					
9736	spruce	3	debris/splinter	split wood/splinter	probably		1	21.0	1.8	0.5								BS
9774	pine	1	Fragment	branch	no	x	1	19.2	1.5	0.9	Kn, CT	ScM	UP	x	x		x	
9790	spruce/larch	3	debris/splinter	split wood/splinter	probably		1	13.8	1.1	0.7								BS
9856	spruce	3	debris/splinter	split wood/splinter	possibly		1	4.7	0.7	0.5	Kn							
9857	pine	3	debris/splinter	split wood/splinter	probably	x	1	11.7	1.6	0.8								
9892	spruce	2	debris/splinter	split wood/splinter	probably	x	1	4.1	1.2	0.2	Kn	Str, TAR	BeC			x	x	
9915	pine	3	debris/splinter	split wood/splinter	probably		1	7.5	1.7	0.6			AS/UP?					BS
9917	pine	3	debris/splinter	split wood/splinter	probably		1	11.1	1.7	0.8					x			
10032	spruce	3	debris/splinter	split wood/splinter	possibly		2	4.1	1.5	0.1	KH							
10033	pine	2	debris/splinter	split wood/splinter	yes	x	1	9.6	2.3	1.4	Kn	CM, RAR, St		x				BS
10034	pine	3	debris/splinter	split wood/splinter	probably		1	6.3	1.8	0.8	Kn		SpN, AS					BS

11316	spruce	3	debris/splinter	split wood/splinter	probably	x	1	13.1	2.0	1.0									
11584	spruce	3	debris/splinter	split wood/splinter	possibly	x	1	5.1	1.0	0.5			taper						
11714	spruce/larch	3	debris/splinter	split wood/splinter	probably		1	11.4	1.7	1.7			taper, UP?	x	x				DC, Del
12113	spruce/larch	1	debris/splinter	branch + split wood/splinter	re-working	x	1	31.0	2.5	2.2	Kn	CM, TF, TAR	SpW, SpN	x		x		x	Del
12294	pine	3	debris/splinter	split wood/splinter	probably	x	1	5.7	1.1	0.8								x	
15151	spruce	3	debris/splinter	split wood/splinter	possibly		1	12.3	1.9	0.8			taper, AS/UP?						
18320	pine	3	debris/splinter	split wood/splinter	probably	x	1	3.0	1.4	0.3									
18322	spruce	3	debris/splinter	split wood/splinter	possibly		1	5.9	1.2	0.2				x					
18560	spruce	3	debris/splinter	split wood/splinter	probably	x	1	14.5	2.3	0.8	Kn					x			
21035	spruce	3	debris/splinter	split wood/splinter	possibly	x	1	7.4	1.0	0.6	Kn								
21038	spruce	3	debris/splinter	split wood/splinter	possibly	x	1	4.2	1.0	0.4									BS
21039	spruce	3	debris/splinter	split wood/splinter	possibly		1	8.8	1.3	0.4	Kn								
21056	pine	3	debris/splinter	split wood/splinter	possibly	x	1	11.3	0.9	0.5		SuF?					x		
18517/1-2	pine	3	debris/splinter	split wood/splinter	probably	x	1	11.3	0.9	0.5									BS
21036/2-3	spruce	3	Fragment	branch	no	x	2	15.0			Kn								BS
mean								9.1	1.4	0.6									
median								7.7	1.4	0.5									

Abbreviations: SB= shaft break; SD= surface damage; delam= delaminated surface; ED= excavation and conservation damage; all other code after Milks et al. 2022.

Table S20. Descriptive statistics of spears.

	n	min	max	median	mean	sd	p25	p75	kurtosis	skewness	CV (%)
Length (cm)	5	182.30	248.50	223.50	216.84	25.40	192.25	238.10	-0.47	-0.28	12
diamMax	8	2.30	4.50	3.30	3.30	0.75	2.60	3.95	-0.59	0.37	23
LMD %	6	18.90	40.80	26.70	28.20	8.90	20.33	36.53	-1.86	0.40	32
Rate of taper P1 (Tp)	7	0.06	0.09	0.07	0.07	0.01	0.06	0.07	2.70	1.52	16
Taper length P1 (cm)	7	20.00	50.00	40.00	35.00	10.40	25.00	40.00	-0.88	-0.16	30
Rate of taper P2 (Tp)	5	0.05	0.11	0.08	0.08	0.02	0.07	0.10	1.44	-0.42	26
Taper length P2 (cm)	5	12.50	22.50	20.00	18.70	3.75	15.50	21.25	2.68	-1.40	20
Annual Rings	8	18	60	51.00	44.50	15.20	31.50	56.50	-0.76	-0.82	34

Length values are calculated only for complete and nearly complete specimens. Complete and nearly complete spears include Spears I, II, III, V and VI. diamMax= maximum diameter; LMD%= location of diamMax measured as distance from the front point and given in percentage of the total length. P1= front point. P2 = back point. Tp= taper, for formula see Materials and Methods in the main text. For raw data, see Table S24.

Table S21. Descriptive statistics of double-pointed sticks (DPSs).

	n	min	max	median	mean	sd	p25	p75	kurtosis	skewness	CV (%)
Length (cm)	3	45.00	77.20	60.20	60.80	16.11	45.00	77.20	-2.33	0.17	26
diamMax (cm)	7	1.70	3.00	2.40	2.40	0.49	1.90	2.90	-1.39	0.06	21
LMD %	6	50.00	71.00	56.30	58.50	9.07	50.38	67.78	-2.06	0.45	16
Rate of taper P1 (Tp)	6	0.03	0.13	0.07	0.08	0.04	0.04	0.12	-2.05	-0.43	56
Taper length P1 (cm)	6	8.00	25.00	20.00	18.40	6.05	13.25	22.90	0.10	-1.08	33
	6	0.04	0.07	0.06	0.06	0.01	0.05	0.06	0.59	-0.67	18
Taper length P2 (cm)	6	15.00	50.00	17.50	23.30	13.66	15.00	31.30	4.20	2.02	59

Length values are calculated only for complete and nearly complete specimens. Complete and nearly complete examples include IDs 1779, 7172, and 10167. diamMax= maximum diameter; LMD%= location of diamMax measured as distance from the front point and given in percentage of the total length. P1= point 1. P2 = point 2. For raw data, see Table S24.

Table S22. Descriptive statistics of pointed split wood tools.

	n	min	max	median	mean	sd	p25	p75	kurtosis	skewness	CV (%)
Length (cm)	24	4.60	36.00	14.50	15.80	8.90	8.80	19.60	0.21	0.95	57
Rate of point taper (Tp)	18	0.03	0.37	0.07	0.09	0.07	0.06	0.10	13.80	3.50	84
Point taper length (cm)	21	4.00	28.50	10.00	10.50	5.70	6.50	12.50	4.20	1.70	54

Tp= taper, for formula see Materials and Methods in the main text. For raw data, see Table S24.

Table S23. Technological complexity data.

	Lower Pleistocene						Middle Pleistocene							
chaîne opératoire **(procedural units*)**													*retooling*	
													discard implement	
													tool use	
													mount implement	
													apply adhesive to shaft	composite tool
													heat to retain elasticity	
													collect adhesive from rock	adhesive (birch tar)
													control temperature	
											retooling		add rock for condensation	
											discard implement		add birch bark	birch bark
											tool use		control temperature	
											binding fibres		ignite fire	
											mounting implement	composite tool	raw material transport	
											tool retouch		raw material selection	fire wood
											flake production		raw material transport	
											core initiation		bark peeling	
									discard	*discard*	raw material transport		raw material selection	birch bark
									tool use	*tool use*	raw material selection	lithic implement	tool retouch	
								discard	surface abrasion	surface abrasion	twisting fibres		flake production	
								tool use	shaping point 1	shaping back point	splitting fibres		core initiation	
								transport	pith offset	pith offset	raw material transport		raw material transport	
						discard	*discard*	edge abrasion	shaping point 1	shaping front point	raw material selection	string	raw material selection	lithic implement
					discard	*tool use*	*tool use*	surface abrasion	shaping shaft	shaping shaft	abrasion		abrasion	
			discard	*discard*	*tool use*	edge retouch	edge abrasion	shaping tool end	flattening branches	flattening branches	socket		socket	
			tool use	*tool use*	tool retouch	thinning	surface abrasion	shaping shaft	debarking	debarking	shaping		shaping	
		discard	shaping point	shaping tool end	flake production	shaping	shaping point	transport	transport	transport	debarking		debarking	
	discard	*tool use*	shaping shaft	shaping shaft	core initiation	transport	shaping shaft	splitting log	initial branch removal	initial branch removal	transport		transport	

	tool use<br><br>raw material selection	tool retouch<br><br>raw material selection	splitting bone<br>raw material selection	splitting bone<br><br>raw material selection	raw material transport<br>raw material selection	rough out<br><br>raw material selection	splitting log/tool<br>raw material selection	tree felling<br><br>raw material selection	tree felling<br><br>raw material selection	tree felling<br><br>raw material selection	tree/branch chopping<br>raw material selection	wooden handle	tree/branch chopping<br><br>raw material selection	wooden handle
**tools used**		smaller stone - retouch<br>hammer stone - production	hammer stone (shaping)<br>wedge (splitting)	hammer stone (shaping)<br><br>wedge (splitting)	smaller stone - retouch<br>hammer stone - production	smaller stone/antler - retouch<br>medium stone/antler - thinning<br>hammer stone - production	stone/reed/bone (abrasion) scraper<br><br>knife<br><br>wedge (splitting)	stone/reed/bone (abrasion) scraper<br><br>knife<br><br>wedge (splitting)<br><br>heavy-duty tool	stone/reed/bone (abrasion) scraper<br><br>knife<br><br>heavy-duty tool	stone/reed/bone (abrasion) scraper<br><br>knife<br><br>heavy-duty tool	smaller stone (retouch)<br><br>hammerstone (lithic)<br><br>stone/reed (abrasion)<br><br>knife (fibre, wood)<br><br>heavy-duty tool (wood)		bark peeler<br><br>smaller stone (retouch)<br><br>hammerstone (lithic)<br><br>rock (condensation)<br><br>stone/reed (abrasion)<br><br>knife (fibre, wood, bark peeling)<br><br>heavy-duty tool (wood)	
**materials used**	stone<br><br>bone	stone	stone<br><br>bone	stone<br><br>bone	stone	antler<br><br>stone	others?<br><br>stone<br><br>wood	others?<br><br>stone<br><br>wood	others?<br><br>stone<br><br>wood	others?<br><br>stone<br><br>wood	fibres<br><br>stone<br><br>wood		adhesive<br><br>birch bark<br><br>fuels (sticks)<br><br>fibres<br><br>stone<br><br>wood	
**product**	bone retoucher	lithic tool<br><br>(on natural chunk)	pointed bone tool	bone fur smoother	lithic flake tool<br><br>(from core)	handaxe<br><br>(thinning)	Round-ended split wood	pointed split wood**	double-pointed stick (DPS)	wooden spears	hafted tool<br><br>(with string)		hafted tool<br><br>(with adhesive)<br><br>method rock condensation	
**References**	Julien et al. 2015	Rocca 2016	Buc 2011	Julien et al. 2015; Arrighi et al 2016	Rocca 2016	Haidle 2012, p. 250	this study	this study	this study	this study	Thieme 2007; Lombard & Haidle 2012		Wragg Sykes 2015, Haidle 2012, Schmidt et al. 2023	

*procedural units after Perrault et al. 2013 (see SI-Text). **assuming off-site procurement, see main text.

Table S24. Raw data used in Tables S20-22.

ID	Length (cm)	Max Diameter (cm)	Group	Max Diameter Location (%)	taper point 1 (Tp)	taper point 2 (Tp)	taper length (cm)	taper length (cm)
Spear I	223.5	4.1	Spear	31.5	0.06	0.08	50	18.5
Spear II	227.7	3.3	Spear	35.1	0.06	NA	40	NA
Spear III	182.3	2.5	Spear	20.8	0.07	0.08	25	12.5
Spear IV	NA	3.2	Spear	NA	NA	0.11	NA	20
Spear V	202.2	2.9	Spear	18.9	0.09	0.05	20	22.5
Spear VI	248.5	4.5	Spear	40.8	0.06	0.09	40	20
Spear VII	NA	3.5	Spear	NA	0.07	NA	40	NA
Spear X	NA	2.3	Spear	21.9	0.07	NA	30	NA
DPS 1779	77.2	2.5	DPS	60.9	0.12	0.04	22.2	50
DPS 7172	60.2	2.1	DPS	71	0.04	0.07	20	15
DPS 10167	45	1.7	DPS	66.7	0.08	0.06	15	15
DPS 15677	NA	3	DPS	51.7	0.13	0.06	8	25
DPS 7344_7362	NA	2.4	DPS	50.5	0.03	0.06	20	20
DPS 7724_7726_7727	NA	1.9	DPS	50	0.05	0.05	25	15
DPS 28108	NA	2.9	DPS	NA	NA	NA	NA	NA
Frag 3635	NA	NA	Point Fragment	NA	NA	NA	NA	NA
Frag 3858	NA	NA	Point Fragment	NA	0.04	NA	16.2	NA
Frag 4236	NA	NA	Point Fragment	NA	0.06	NA	20	NA
Frag 4934	NA	NA	Point Fragment	NA	0.08	NA	8.9	NA
Frag 5600	NA	NA	Point Fragment	NA	0.13	NA	4.5	NA
Frag 6923	NA	NA	Point Fragment	NA	0.05	NA	15	NA
Frag 6935	NA	NA	Point Fragment	NA	0.07	NA	10.7	NA
Frag 7567	NA	NA	Point Fragment	NA	NA	0.05	NA	25
Frag 8674	NA	NA	Point Fragment	NA	NA	0.06	NA	10.8
Frag 8689	NA	NA	Point Fragment	NA	0.04	NA	8.6	NA
Frag 9388	NA	NA	Point Fragment	NA	0.08	NA	18.2	NA
Frag 9772	NA	NA	Point Fragment	NA	0.06	NA	20	NA
Frag 9788	NA	NA	Point Fragment	NA	0.11	NA	5	NA

Frag 12074 Former Spear IX	NA	1.8	Point Fragment	NA	0.06	NA	20	NA
Frag 14852 Former Spear VIII	NA	2.8	Point Fragment	NA	0.07	NA	30	NA
Frag 15146	NA	NA	Point Fragment	NA	NA	0.13	NA	10
Frag 18342	NA	NA	Point Fragment	NA	NA	0.07	NA	10
pSpW 6826	17.5	NA	pointed SpW	NA	0.04	NA	12.5	NA
pSpW 7031	20.3	NA	pointed SpW	NA	0.03	NA	15	NA
pSpW 7243	28.5	NA	pointed SpW	NA	0.06	NA	28.5	NA
pSpW 7245	10.5	NA	pointed SpW	NA	0.11	NA	10	NA
pSpW 7320	9.5	NA	pointed SpW	NA	0.07	NA	9	NA
pSpW 7503	15.7	NA	pointed SpW	NA	NA	NA	7.5	NA
pSpW 7832	15.9	NA	pointed SpW	NA	0.06	NA	12.5	NA
pSpW 7878	15.8	NA	pointed SpW	NA	0.1	NA	7.5	NA
pSpW 7898	6.5	NA	pointed SpW	NA	NA	NA	5	NA
pSpW 8070	13.5	NA	pointed SpW	NA	0.07	NA	12	NA
pSpW 8097	4.6	NA	pointed SpW	NA	0.07	NA	4	NA
pSpW 9109	11.8	NA	pointed SpW	NA	0.06	NA	10	NA
pSpW 9368	11.4	NA	pointed SpW	NA	NA	NA	10	NA
pSpW 9424	15.5	NA	pointed SpW	NA	NA	NA	NA	NA
pSpW 9775	5.9	NA	pointed SpW	NA	0.12	NA	5.5	NA
pSpW 9776	8.5	NA	pointed SpW	NA	0.07	NA	8	NA
pSpW 10170	36	NA	pointed SpW	NA	NA	NA	NA	NA
pSpW 10172	13.3	NA	pointed SpW	NA	0.09	NA	10	NA
pSpW 12061	17.2	NA	pointed SpW	NA	0.06	NA	14	NA
pSpW 14903	34.7	NA	pointed SpW	NA	0.07	NA	10	NA
pSpW 18244	8.1	NA	pointed SpW	NA	NA	NA	NA	NA
pSpW 18304	4.8	NA	pointed SpW	NA	0.05	NA	4.5	NA
pSpW 7501_7507	23.1	NA	pointed SpW	NA	0.1	NA	5	NA
pSpW 9920_9922	29.6	NA	pointed SpW	NA	0.05	NA	20	NA
NN18962	325	2.3	Ethno Spear	58	NA	NA	NA	NA
NN18960	279	2.3	Ethno Spear	20	NA	NA	NA	NA
NN18952	276	2.4	Ethno Spear	25	NA	NA	NA	NA
NN18950	282	1.8	Ethno Spear	55	NA	NA	NA	NA

NN18958	301	2.1	Ethno Spear	30	NA	NA	NA	NA
34,82	302	2.3	Ethno Spear	29	NA	NA	NA	NA
NN18963	321.5	2.1	Ethno Spear	26	NA	NA	NA	NA
NN18964	311.3	1.9	Ethno Spear	33	NA	NA	NA	NA
NN18961	314.5	2.1	Ethno Spear	32	NA	NA	NA	NA
8.3.50/1	275.5	2.8	Ethno Spear	33	NA	NA	NA	NA
NN18954	263	1.9	Ethno Spear	25	NA	NA	NA	NA
6,179	199	1.8	Ethno Spear	48	NA	NA	NA	NA
6,181	248	2	Ethno Spear	40	NA	NA	NA	NA
NN18903	225	1.5	Ethno Spear	27	NA	NA	NA	NA
NN18949	248.5	2.1	Ethno Spear	59	NA	NA	NA	NA
NN18880	234.5	2.7	Ethno Spear	23	NA	NA	NA	NA
NN18902	223.5	1.3	Ethno Spear	32	NA	NA	NA	NA
NN18970	206	1.9	Ethno Spear	27	NA	NA	NA	NA
NN18888	217.5	1.3	Ethno Spear	9	NA	NA	NA	NA
1917,118	245	1.9	Ethno Spear	23	NA	NA	NA	NA
30.455A	343	2.5	Ethno Spear	45	NA	NA	NA	NA
Z 42280	137.5	1.3	Ethno Spear	50	NA	NA	NA	NA
1947,1	247	2.3	Ethno Spear	24	NA	NA	NA	NA
1919.5.2	262	2.5	Ethno Spear	54	NA	NA	NA	NA
Z 6377	211	2.7	Ethno Spear	57	NA	NA	NA	NA
TEMP.001	281	2.1	Ethno Spear	36	NA	NA	NA	NA
TEMP.001	227.5	1.6	Ethno Spear	58	NA	NA	NA	NA
z 28540	323	2.3	Ethno Spear	25	NA	NA	NA	NA
A39396	223	2.7	Ethno Spear	61	NA	NA	NA	NA
A21570	277	2.7	Ethno Spear	50	NA	NA	NA	NA
A54511	229.5	2.3	Ethno Spear	66	NA	NA	NA	NA
A35145	261	1.8	Ethno Spear	38	NA	NA	NA	NA
A34864	248.5	1.5	Ethno Spear	40	NA	NA	NA	NA
A54516	246	2.8	Ethno Spear	38	NA	NA	NA	NA
A31115	204.5	2.6	Ethno Spear	47	NA	NA	NA	NA
A5173/39	329	3.2	Ethno Spear	61	NA	NA	NA	NA
E81037	313.5	2.8	Ethno Spear	47	NA	NA	NA	NA
E81038	307	2.7	Ethno Spear	52	NA	NA	NA	NA

E.11501	293.5	1.8	Ethno Spear	39	NA	NA	NA	NA
E.11500	251	1.8	Ethno Spear	50	NA	NA	NA	NA
E.11499	300.5	1.8	Ethno Spear	28	NA	NA	NA	NA
E.5381	357	2.2	Ethno Spear	39	NA	NA	NA	NA
E.6167	345	2.4	Ethno Spear	54	NA	NA	NA	NA
E.11359	323.5	2.5	Ethno Spear	44	NA	NA	NA	NA
E.12923	245	2.8	Ethno Spear	25	NA	NA	NA	NA
E.10755	324.5	1.8	Ethno Spear	19	NA	NA	NA	NA
E.10756	324	1.5	Ethno Spear	6	NA	NA	NA	NA
E.9374	275	2.7	Ethno Spear	25	NA	NA	NA	NA
E.31759	239.5	2	Ethno Spear	66	NA	NA	NA	NA
E.1520	275	2	Ethno Spear	41	NA	NA	NA	NA
E.31756	327.5	2.3	Ethno Spear	31	NA	NA	NA	NA
E.20093	194.5	1.6	Ethno Spear	6	NA	NA	NA	NA
M.2721	368	2.1	Ethno Spear	13	NA	NA	NA	NA
M.2724	293	1.4	Ethno Spear	12	NA	NA	NA	NA
M.2723	301	1.3	Ethno Spear	16	NA	NA	NA	NA
M.2722	416.5	1.8	Ethno Spear	4	NA	NA	NA	NA
M.5902	438.5	2.1	Ethno Spear	13	NA	NA	NA	NA
M.1207	400	1.8	Ethno Spear	16	NA	NA	NA	NA
